# Effects of a Lifestyle Intervention to Prevent Deterioration in Glycemic Status Among South Asian Women With Recent Gestational Diabetes: A Randomized Clinical Trial

**DOI:** 10.1001/jamanetworkopen.2022.0773

**Published:** 2022-03-02

**Authors:** Nikhil Tandon, Yashdeep Gupta, Deksha Kapoor, Josyula K. Lakshmi, Devarsetty Praveen, Amritendu Bhattacharya, Laurent Billot, Aliya Naheed, Asita de Silva, Ishita Gupta, Noshin Farzana, Renu John, Saumiyah Ajanthan, Hema Divakar, Neerja Bhatla, Ankush Desai, Arunasalam Pathmeswaran, Dorairaj Prabhakaran, Rohina Joshi, Stephen Jan, Helena Teede, Sophia Zoungas, Anushka Patel

**Affiliations:** 1 Department of Endocrinology and Metabolism, All India Institute of Medical Sciences, New Delhi, India; 2 George Institute for Global Health, New Delhi, India; 3 Faculty of Medicine and Health, University of New South Wales, Sydney, Australia; 4 Prasanna School of Public Health, Manipal Academy of Higher Education, Manipal, India; 5 George Institute for Global Health, Hyderabad, India; 6 Initiative for Noncommunicable Diseases, Health Systems and Population Studies Division, International Centre for Diarrhoeal Disease Research (ICDDR, B), Dhaka, Bangladesh; 7 Faculty of Medicine, University of Kelaniya, Kelaniya, Sri Lanka; 8 Centre for Chronic Disease Control, New Delhi, India; 9 Remedium One, Colombo, Sri Lanka; 10 Divakars Speciality Hospital, Bengaluru, India; 11 Department of Obstetrics and Gynaecology, All India Institute of Medical Sciences, New Delhi, India; 12 Department of Medicine, Goa Medical College, Goa, India; 13 Department of Public Health, Faculty of Medicine, University of Kelaniya, Sri Lanka; 14 Public Health Foundation of India, New Delhi, India; 15 London School of Hygiene and Tropical Medicine, London, United Kingdom; 16 Monash Centre for Health Research and Implementation, School of Public Health and Preventive Medicine, Monash University, Melbourne, Australia; 17 School of Public Health and Preventive Medicine, Monash University, Melbourne, Australia

## Abstract

**Importance:**

Women with recent gestational diabetes (GDM) have increased risk of developing type 2 diabetes.

**Objective:**

To investigate whether a resource-appropriate and context-appropriate lifestyle intervention could prevent glycemic deterioration among women with recent GDM in South Asia.

**Design, Setting, and Participants:**

This randomized, participant-unblinded controlled trial investigated a 12-month lifestyle intervention vs usual care at 19 urban hospitals in India, Sri Lanka, and Bangladesh. Participants included women with recent diagnosis of GDM who did not have type 2 diabetes at an oral glucose tolerance test (OGTT) 3 to 18 months postpartum. They were enrolled from November 2017 to January 2020, and follow-up ended in January 2021. Data were analyzed from April to July 2021.

**Interventions:**

A 12-month lifestyle intervention focused on diet and physical activity involving group and individual sessions, as well as remote engagement, adapted to local context and resources. This was compared with usual care.

**Main Outcomes and Measures:**

The primary outcome was worsening category of glycemia based on OGTT using American Diabetes Association criteria: (1) normal glucose tolerance to prediabetes (ie, impaired fasting glucose or impaired glucose tolerance) or type 2 diabetes or (2) prediabetes to type 2 diabetes. The primary analysis consisted of a survival analysis of time to change in glycemic status at or prior to the final patient visit, which occurred at varying times after 12 months for each patient. Secondary outcomes included new-onset type 2 diabetes and change in body weight.

**Results:**

A total of 1823 women (baseline mean [SD] age, 30.9 [4.9] years and mean [SD] body mass index, 26.6 [4.6]) underwent OGTT at a median (IQR) 6.5 (4.8-8.2) months postpartum. After excluding 160 women (8.8%) with type 2 diabetes, 2 women (0.1%) who met other exclusion criteria, and 49 women (2.7%) who did not consent or were uncontactable, 1612 women were randomized. Subsequently, 11 randomized participants were identified as ineligible and excluded from the primary analysis, leaving 1601 women randomized (800 women randomized to the intervention group and 801 women randomized to usual care). These included 600 women (37.5%) with prediabetes and 1001 women (62.5%) with normoglycemia. Among participants randomized to the intervention, 644 women (80.5%) received all program content, although COVID-19 lockdowns impacted the delivery model (ie, among 644 participants who engaged in all group sessions, 476 women [73.9%] received some or all content through individual engagement, and 315 women [48.9%] received some or all content remotely). After a median (IQR) 14.1 (11.4-20.1) months of follow-up, 1308 participants (81.2%) had primary outcome data. The intervention, compared with usual care, did not reduce worsening glycemic status (204 women [25.5%] vs 217 women [27.1%]; hazard ratio, 0.92; [95% CI, 0.76–1.12]; *P* = .42) or improve any secondary outcome.

**Conclusions and Relevance:**

This study found that a large proportion of women in South Asian urban settings developed dysglycemia soon after a GDM-affected pregnancy and that a lifestyle intervention, modified owing to the COVID-19 pandemic, did not prevent subsequent glycemic deterioration. These findings suggest that alternate or additional approaches are needed, especially among high-risk individuals.

**Trial Registration:**

Clinical Trials Registry of India Identifier: CTRI/2017/06/008744; Sri Lanka Clinical Trials Registry Identifier: SLCTR/2017/001; and ClinicalTrials.gov Identifier: NCT03305939

## Key Points

**Question:**

Can an evidence-based lifestyle intervention, adapted to local context, prevent glycemic deterioration among women with recent gestational diabetes (GDM) in South Asia?

**Findings:**

In this randomized clinical trial of 1612 women with recent GDM, 25.5% of participants allocated to a lifestyle intervention and 27.1% of participants allocated to usual care experienced worsening glycemia at a median of 14.1 months of follow-up. The intervention did not prevent glycemic deterioration, including development of diabetes.

**Meaning:**

This study found that a lifestyle intervention was not effective in reducing glycemic deterioration, which occurred in a substantial proportion of women with recent GDM within 2 years of childbirth.

## Teaser

This randomized clinical trial examines a lifestyle intervention vs usual care for prevention of glycemic deterioration among South Asian women with recent gestational diabetes.

## Introduction

Gestational diabetes (GDM) prevalence is increasing globally and particularly in South Asia.^1^ Women with GDM are at increased risk of developing type 2 diabetes.^2,3,4,5^ Parous women with prediabetes have an increased risk of developing type 2 diabetes if they have a history of GDM compared with those without prior GDM.^6^

Randomized trials have found that lifestyle interventions can delay or prevent type 2 diabetes among individuals with prediabetes.^7^ Findings from a subgroup analysis of 350 participants in the Diabetes Prevention Program (DPP) study suggested that a lifestyle intervention was similarly effective among women with dysglycemia and previous GDM compared with parous women with dysglycemia but no history of GDM, with an approximately 50% reduction in the incidence of type 2 diabetes over 3 years compared with placebo.^6^ However, participants were randomized at a mean interval of 12 years after their index pregnancy. Systematic reviews of lifestyle intervention trials focused on women with GDM, mostly earlier postpartum, had mixed results for a range of surrogate outcomes derived from small studies, few of which had a low risk of bias.^8,9,10,11,12^ Furthermore, most interventions have been tested in high-income countries, often with intensive programs delivered by highly trained staff.^8^ Whether a pragmatic, scalable, and sustainable lifestyle intervention delivered by staff currently available within South Asian health systems can produce benefits is unknown.

This study, the Lifestyle Intervention in Gestational Diabetes (LIVING) study, is a randomized implementation trial of a pragmatic (ie, addressing the real-world effectiveness of a clinically relevant lifestyle intervention in a diverse population of study participants) lifestyle intervention program among South Asian women with a recent GDM-affected pregnancy.^13^ The aim was to investigate whether such an intervention would prevent deterioration in glycemic status among women who had not already developed type 2 diabetes.

## Methods

This randomized clinical trial reports outcomes of a participant-unblinded, parallel-group individual randomized trial in 19 urban hospitals in India, Sri Lanka, and Bangladesh (trial protocol in Supplement 1). It is reported following the Consolidated Standards of Reporting Trials Extension (CONSORT Extension) reporting guideline. Ethics review committees of the All India Institute of Medical Sciences (India), ICDDR, B (Bangladesh), University of Kelaniya (Sri Lanka), and University of Sydney (Australia), and individual hospitals where required approved the study. All participants gave written informed consent.

### Study Population

Study participants were women diagnosed with GDM within the previous 18 months with an oral glucose tolerance test (OGTT) at from 24 to 34 weeks’ gestation based on the International Association of the Diabetes and Pregnancy Study Groups (IADPSG) criteria. Given that routine GDM screening at some centers occurred earlier than 24 weeks’ gestation, additional criteria were accepted (eMethods 1 in Supplement 2).

Potential participants had an OGTT from 3 months to 18 months postpartum. Exclusion criteria were type 2 diabetes based on OGTT, a travel time to the hospital of more than 2 hours, unavailability of a household mobile telephone, use of steroids during pregnancy (other than for fetal lung maturation), and likelihood of moving to a new residence within 3 years.

### Randomization

Participants were allocated by central computer using randomly permuted blocks of size 4 and 6 and stratified by study center and index pregnancy insulin use. They were randomized at a ratio of 1:1 to a 12-month lifestyle intervention or usual care. Central study staff were blinded to participant allocation, but this was not possible for site personnel or participants. Laboratories were unaware of participant allocation, and reports were verified against database entries by a central blinded observer.

### Interventions

**Figure 1.**
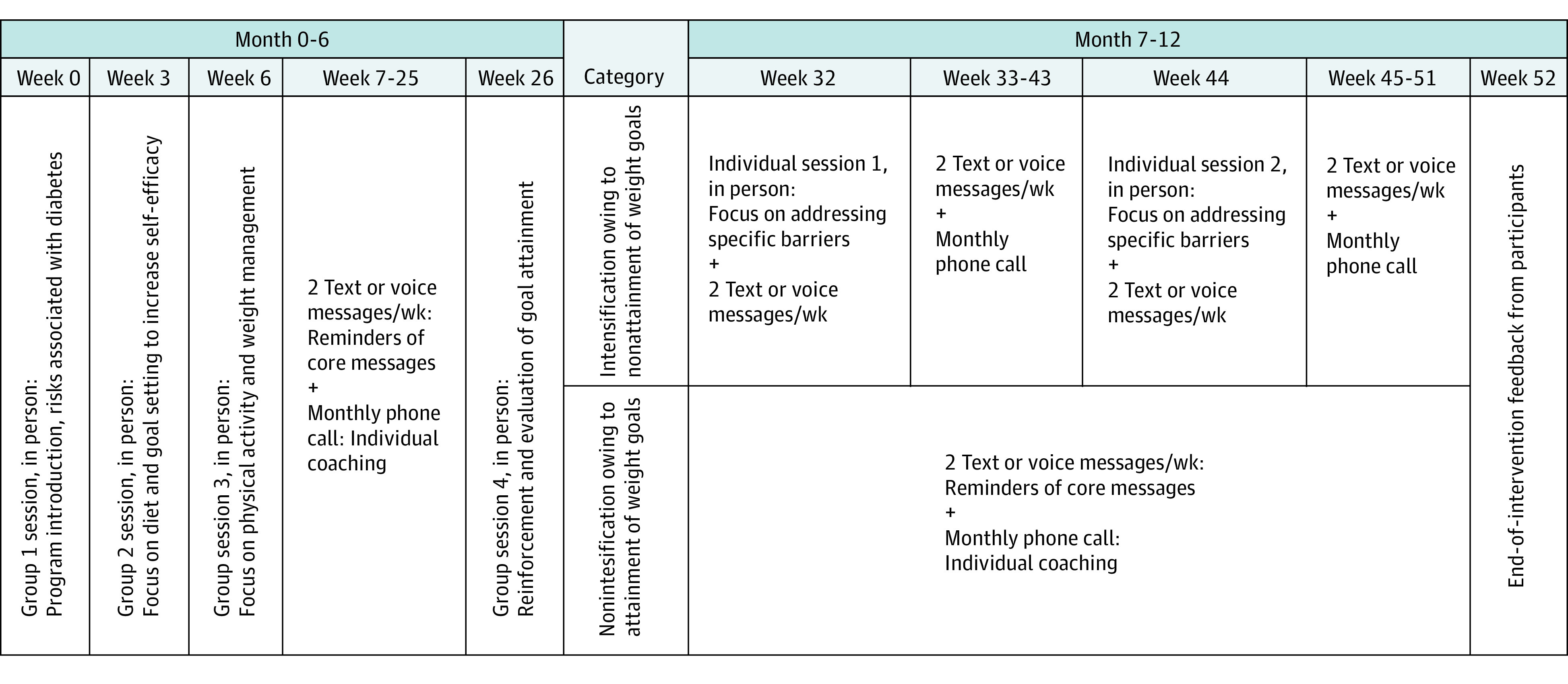
Planned Intervention There were 84 text or voice messages and 9 phone calls from facilitators planned for each participant.

Participants randomized to the lifestyle intervention were invited to participate in a 12-month program based on prior approaches associated with preventing weight gain in post-GDM populations^14,15^ and modified through mixed-methods formative research in each country.^16^ The planned program (Figure 1; eMethods 2 in Supplement 2; trial protocol in Supplement 1) included four 90-minute face-to-face group sessions over 6 months followed by 2 face-to-face individual sessions for individuals who were persistently overweight or had gained more than 2% of baseline body weight. At group sessions, facilitators aimed to provide core messages and lead activities with a focus on diet and physical activity. Intervention group participants were planned to receive detailed written program content, as well as a total of 84 prerecorded voice or text messages over a 42-week period and monthly follow-up calls after completion of group sessions. Facilitators were equivalent to existing roles within each health system, which varied by country (ie, counselors with a sociology background in Bangladesh, nurses in Sri Lanka, and nurse auxiliaries in India) and were provided with specific training for program delivery. No attempt was made to influence treatment of participants receiving usual care.

### Study Procedures

Follow-up visits were conducted every 6 months after baseline assessment. Using local laboratories, an OGTT was performed at each annual visit and the end of follow-up. Glycosylated hemoglobin (HbA_1c_) was measured at interim 6-month visits, followed by an OGTT if the HbA_1c_ was 6.5% (48 mmol HbA_1c_/mol Hb) or greater, and at the end of follow-up. Data on blood pressure, anthropometry, diet, and physical activity were collected at each visit. Blood pressure was measured using an automated sphygmomanometer (Omron JPN1), with a mean of 2 readings after 5 minutes of rest recorded. Body weight was measured using a digital scale (Omron HN286) with the individual wearing light clothing, while waist circumference was measured halfway between the lowest rib palpable in the midaxillary line and the top of the iliac crest. Dietary intake information was collected using local adaptations of the Intake24 dietary recall system (Food Standards Scotland, Newcastle University, and the University of Cambridge),^17^ and physical activity levels were assessed using the Modified Global Physical Activity Questionnaire.^18^ Planned termination of follow-up occurred with a verified nonstudy diagnosis of type 2 diabetes or if a participant became pregnant.

### Primary Outcome

The primary outcome was deterioration in glycemia, consisting of development of type 2 diabetes or progression from normal glucose tolerance (NGT) to prediabetes (ie, impaired fasting glucose [IFG] or impaired glucose tolerance [IGT]). Glycemic category was defined using American Diabetes Association criteria,^19^ based on fasting and 2-hour plasma glucose levels from the OGTT: NGT: less than 100 mg/dL (to convert to millimoles per liter, multiply by 0.0555) fasting and less than 140 mg/dL at 2 hours; IFG: 100 to 125 mg/dL fasting and less than 140 mg/dL at 2 hours; IGT: less than 100 mg/dL fasting and 140 to 199 mg/dL at 2 hours; IFG and IGT: 100 to 125 mg/dL fasting and 140 to 199 mg/dL at 2 hours; and type 2 diabetes: 126 mg/dL or more fasting or 200 mg/dL or more at 2 hours. For a prespecified sensitivity analysis additional, follow-up measures of glycemia (HbA_1_c or fasting plasma glucose) (eFigure 1 in Supplement 2) were used when OGTT results were unavailable.

### Secondary and Other Outcomes

Prespecified secondary outcomes were progression to type 2 diabetes and mean changes in fasting plasma glucose, systolic blood pressure, body weight, waist circumference, physical activity levels, and caloric intake. Other prespecified outcomes were mean changes in heart rate, diastolic blood pressure, hip circumference, daily moderate activity, daily sedentary activity, sleep duration, and intake of specific dietary components. Review after database lock revealed infeasible values for overall physical activity levels, which could not be reliably reported. After post hoc application of feasibility ranges, data among 1406 study participants contributed to analysis on daily moderate activity (195 individuals excluded [12.2%]) and data among 1495 participants contributed to analysis on daily sedentary activity and sleep duration (106 individuals excluded [6.6%]).

### Statistical Analysis

A sample size of 1414 women was estimated to provide 90% power (2-sided α = .05) to detect a 35% relative decrease in worsening glycemic status, assuming a 20% cumulative incidence in the control group at 24 months (conservative assumptions projected from DPP^6^) and 20% missing outcome data. The initial power calculation was based on a χ^2^ test comparing proportions at 24 months; however, recruitment required more time than originally anticipated, resulting in a range of follow-up durations; thus, the primary analysis consisted of a survival analysis of time to change in glycemic status at or prior to the final patient visit, which occurred at varying times after 12 months for each patient. Keeping the original assumptions constant, a survival analysis using a log-rank test with 14 months of median effective follow-up had approximately 80% power.

In accordance with the statistical analysis plan (Supplement 1),^20^ all analyses were based on the intention-to-treat principle, with exclusion of participants subsequently found to be ineligible. A sensitivity analysis for the primary outcome included these participants. The effects of the intervention on the primary end point were estimated from a Cox proportional hazard model, with randomized group and use of insulin during index pregnancy as fixed effects and study center as a random effect. The Cox model assumed a constant hazard ratio (HR) over the follow-up, with the HR corresponding to the mean difference in risk of event between study groups over the study period. Adjusted analyses were performed by adding age, baseline glycemic category, body mass index (BMI; calculated as weight in kilograms divided by height in meters squared) category, and time since GDM-affected pregnancy to the model. For participants with repeated-outcome events during follow-up, survival time to the first relevant end point was used. Participants with no event were censored at the date of last OGTT. A similar approach was taken for the secondary outcome of type 2 diabetes. For other secondary outcomes, which were continuous variables, repeated-measure linear mixed models were used to assess differences between groups over time. Model fixed effects included the randomized group, visit and group by visit interaction, use of insulin during index pregnancy, and baseline value of the outcome. Random intercepts by participant and study center were added to the model.

Prespecified sensitivity analyses included using HbA_1c_or fasting plasma glucose when OGTT results were not available. Additionally, Poisson regression was used in place of Cox models.

Separate estimates for treatment effects on the primary outcome were obtained among prespecified subgroups of participants defined by the following baseline characteristics: age (ie, >median vs ≤median), baseline glycemic status (ie, normoglycemia vs prediabetes), gravida (ie, 1 vs >1 pregnancies), country, BMI (ie, underweight [ie, <18.5], normal [ie, 18.5-24.9], overweight [ie, 25-29.9], or obese [≥30), insulin use during index pregnancy, and time since GDM-affected pregnancy (ie, >median vs ≤median). Estimates were obtained by adding the corresponding subgroup variable to the Cox model as a fixed effect together with its interaction with the randomized group. Heterogeneity of treatment effect was quantified by the *P* value associated with the interaction term.

No imputation for missing data was done. Statistical significance is based on a 2-sided type I error rate of 5%. A Holm-Bonferroni adjustment was used to control the family-wise error rate across secondary outcomes.^21^ All analyses were conducted using SAS Enterprise Guide statistical software version 7.15 (SAS Institute). Data were analyzed from April to July 2021.

## Results

### Study Participants

**Figure 2.**
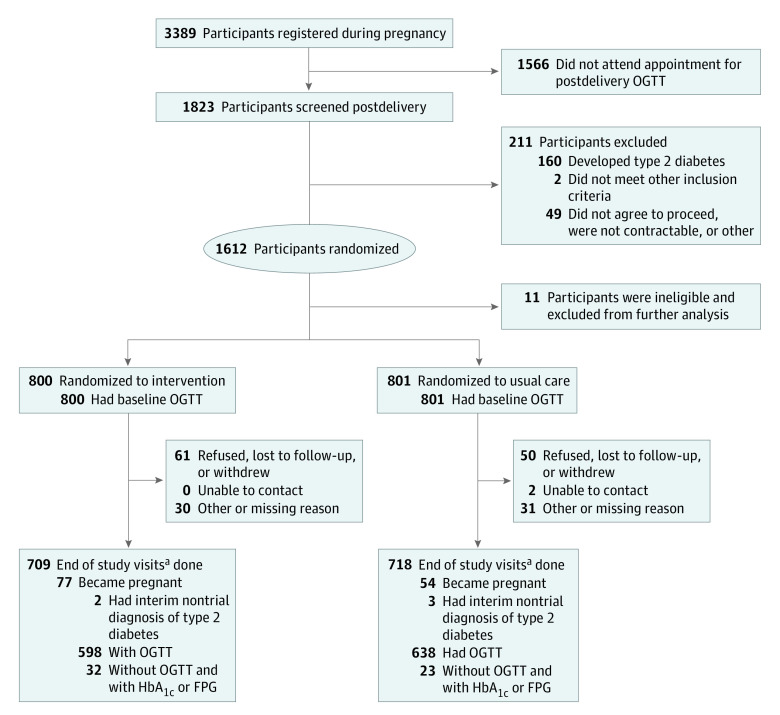
Participant Flowchart End-of-study visits were conducted at least 12 months postrandomization between July 1, 2020, and January 31, 2021. FPG indicates fasting plasma glucose; HbA_1c_, hemoglobin A_1c_; OGTT, oral glucose tolerance test.

Of 3389 registered patients with GDM, 1823 individuals had an OGTT at a median (IQR) of 6.5 (4.8-8.2) months postpartum (Figure 2; eFigure 2 in Supplement 2). Of these, 160 individuals (8.8%) had type 2 diabetes, 51 individuals were excluded for other reasons (2 women [0.1%] met other exclusion criteria, and 49 women [2.7%] did not consent or were uncontactable), 1612 individuals were randomized between November 2017 and January 2020. Additionally, of 1823 individuals with an OGTT, 621 individuals (34.1%) had prediabetes. Eleven randomized participants were subsequently identified as ineligible and excluded from the primary analysis, leaving 1601 women (800 women in the intervention group and 801 women in the usual care group). GDM had been diagnosed by IADPSGS criteria in 1421 of 1612 randomized participants (88.2%).

Baseline characteristics were similar between randomized groups (Table 1), with some variation by country (eTable 1 in Supplement 2). The mean (SD) age was 30.9 (4.9) years, and mean (SD) BMI was 26.6 (4.6); 234 participants (14.6%) required insulin during index pregnancy. At baseline, 1001 women (62.5%) had normoglycemia and 600 women (37.5%) had prediabetes (including 240 women with IFG [15.0%], 188 women with IGT [11.7%], and 172 women with IFG and IGT [10.7%]).

**Table 1.** Baseline Characteristics

Characteristic	Participants, No. (%)^a^	
	Intervention (n = 800)	Usual care (n = 801)
Age, mean (SD), y	30.7 (4.8)	31.0 (5.0)
Country		
Bangladesh	187 (23.4)	184 (23.0)
India	390 (48.8)	390 (48.7)
Sri Lanka	223 (27.9)	227 (28.3)
Formal education, mean (SD), y	12.7 (3.5)	12.8 (3.6)
Currently employed	144 (18.0)	159 (19.9)
Gravida, median (IQR)	2 (1-3)	2 (1-3)
Time since delivery, mean (SD), mo	6.8 (2.8)	6.9 (2.9)
Prior nonindex pregnancy history of gestational diabetes	64 (8.0)	57 (7.1)
Insulin use during index pregnancy	120 (15.0)	114 (14.2)
Family history in first-degree relatives		
Of diabetes	386 (48.3)	389 (48.6)
Of hypertension	303 (37.9)	307 (38.3)
Self-reported use		
Tobacco	0/798 (0)	0/799 (0)
Alcohol	41/799 (5.1)	33/798 (4.1)
Body weight, mean (SD), kg	63.1 (12.2)	63.8 (11.8)
BMI		
Mean (SD)	26.5 (4.6)	26.6 (4.7)
Category		
<18.5	20 (2.5)	24 (3.0)
18.5-24.9	301 (37.6)	277 (34.6)
25.0-29.9	310 (38.8)	321 (40.1)
≥30.0	169 (21.1)	178 (22.3)
Waist circumference, mean (SD), cm	89.3 (11.8)	89.9 (12.1)
HbA_1c_, median (IQR), %	5.4 (5.1-5.8)	5.5 (5.2-5.8)
Fasting plasma glucose, mean (SD), mg/dL	92.7 (10.6)	94.0 (11.2)
Glycemic status		
Normoglycemia	527 (65.9)	474 (59.2)
IFG	109 (13.6)	131 (16.4)
IGT	91 (11.4)	97 (12.1)
IFG and IGT	73 (9.1)	99 (12.4)
Blood pressure, mean (SD), mm Hg		
Systolic	112.6 (11.2)	112.7 (11.2)
Diastolic	74.4 (9.1)	74.7 (9.2)
Total intake, mean (SD)		
Calorie, Kcal/d	1643 (542)	1664 (548)
Carbohydrate, g/d	268.3 (91.8)	269.6 (92.0)
Protein, g/d	58.1 (26.0)	58.2 (33.2)
Fat, g/d	41.7 (22.6)	43.5 (24.6)
Fiber, g/d	13.8 (9.7)	14.1 (10.5)
Sodium, g/d	7.0 (3.8)	7.0 (3.8)
Moderate physical activity, mean (SD), minutes/d	257 (138)	250 (133)
Sedentary activity, mean (SD), min/d	218 (224)	220 (228)
Sleep duration, mean (SD), min/d	399 (80)	403 (82)

Abbreviations: BMI, body mass index (calculated as weight in kilograms divided by height in meters squared); HbA_1c_, hemoglobin A_1c_; IFG, impaired fasting glucose; IGT, impaired glucose tolerance.

^a^ Data were missing data for 1 participant in usual care for anthropometric measurements, 1 participant in intervention and 2 participants in usual care for blood pressure, 7 participants in intervention and 2 participants in usual care for dietary data, and 41 participants in intervention and 42 participants in usual care for moderate physical activity.

### Intervention Fidelity

Of 800 participants randomized to the lifestyle intervention, 717 women (89.6%) received at least some content and 644 women (80.5%) received all content, delivered as originally planned or through an alternate mode (eFigure 3 in Supplement 2). Intervention fidelity was affected by slow initial recruitment (delaying formation of sufficiently large groups to commence) and subsequently by COVID-19 lockdowns. Consequently, among 644 participants who engaged in all group sessions, 476 women (73.9%) received some or all content through individual engagement and 315 women (48.9%) received some or all content remotely rather than in person. Prior to COVID-19 lockdowns, 139 intervention group participants who had completed group sessions (42.3%) were offered intensification sessions owing to nonachievement of weight goals, and 129 of these individuals (92.8%) received at least 1 such session. After lockdowns, because of difficulty in reliably assessing body weight, intensification sessions were offered to all intervention group participants, and 311 individuals (98.4%) received at least 1 such session.

### Primary Outcome

**Figure 3.**
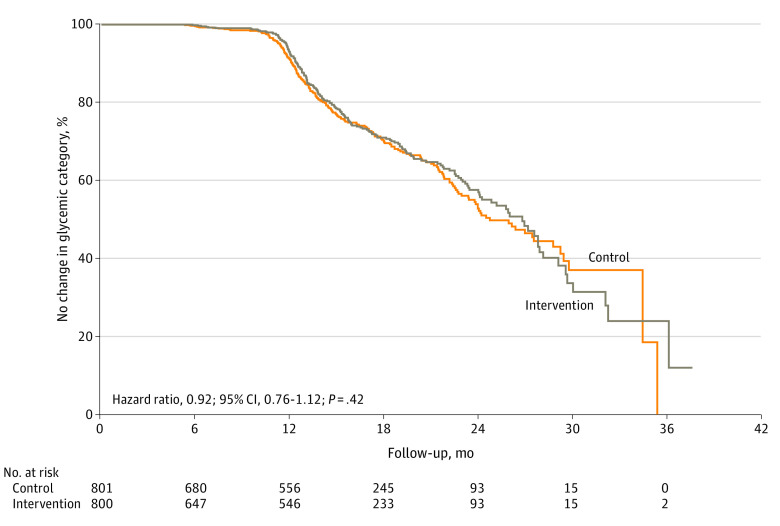
Kaplan-Meier Plot for Primary Outcome The primary analysis consisted of a survival analysis of time to change in glycemic status at or prior to the final patient visit, which occurred at varying times after 12 months for each patient. Proportions shown in the plot are a function of the number of patients experiencing an event by a certain time in the subset of patients who were still at risk at that time (ie, after excluding patients who had already experienced an event or been censored). The curve in the usual care group decreases to 0% by 36 months, indicating that by 36 months, all patients had been censored or experienced an event (ie, no patient was still at risk).

Median (IQR) follow-up was 14.0 (11.4-19.8) months for the intervention group and 14.3 (11.5-20.3) months for the usual care group. Participant disposition by visit is outlined in eTable 2 in Supplement 2. A total of 1308 participants (81.7%) had an end-of-study follow-up OGTT or at least 1 follow-up OGTT, which increased to 1438 individuals (89.8%) when HbA_1c_ or fasting plasma glucose were also considered. Worsening of glycemic status occurred in 421 participants: 204 individuals (25.5%) in the lifestyle intervention group and 217 individuals (27.1%) in the usual care group, and there was no statistically significant difference in risk (HR, 0.92 [95% CI, 0.76-1.12]; *P* = .42) (Figure 3). In sensitivity analyses using Poisson models, including all randomized participants, and using alternate measures of glycemia among individuals without an OGTT, there was still no statistically significant difference in risk (eTable 3 in Supplement 2).

### Secondary Outcomes

There were no statistically significant differences between randomized groups in any secondary outcomes (Table 2; eFigure 4 in Supplement 2). Type 2 diabetes developed in 154 individuals (74 participants [9.3%] in the intervention group and 80 individuals [10.0%] in the usual care group; hazard ratio, 0.89 [95% CI, 0.69-1.23]; *P* = .48) (eFigure 5 in Supplement 2). Type 2 diabetes developed among 122 of 600 individuals with prediabetes at baseline (20.3%) and 32 of 1001 individuals with baseline normoglycemia (3.2%). Body weight increased in both groups by 0.4 kg despite a decrease in caloric intake. There were small decreases overall in moderate physical activity levels and sedentary behavior, with no statistically significant between-group differences. Sleep duration, fasting plasma glucose, and blood pressure increased in both groups, without any statistically significant between-group differences. There were no statistically significant between-group differences for other prespecified outcomes (eTable 4 in Supplement 2). Ultimately, given that *P* values for all outcomes were nonsignificant, no adjustment for multiple comparisons was made.

**Table 2.** Effects of Intervention on Secondary Outcomes

Outcome^a^	Participants, No.^b^	Mean (SE)		HR or mean difference (95% CI)	*P* value^c^
		Intervention	Usual care		
Development of type 2 diabetes, No. (%)	1601	74 (9.3)	80 (10.0)	0.89 (0.65 to 1.23)	.48
Changes in outcome measures					
FPG, mg/dL	1327	8.6 (1.6)	7.4 (1.5)	1.1 (−1.5 to 3.8)	.41
Body weight, kg	1404	0.4 (0.2)	0.4 (0.2)	0.0 (−0.5 to 0.5)	.93
Waist circumference, cm	1395	0.0 (0.5)	−0.3 (0.5)	0.4 (−0.5 to 1.2)	.43
SBP, mm Hg	1401	1.9 (0.6)	1.9 (0.5)	0.0 (−1.0 to 1.1)	.95
Caloric intake, Kcal/d	1419	−275 (67)	−238 (66)	−37 (−90 to 16)	.17

Abbreviations: FPG, fasting plasma glucose; HR, hazard ratio; SBP, systolic blood pressure.

SI conversion factors: To convert glucose to millimoles per liter, multiply by 0.0555.

^a^ Cox model was used to analyze time to development of type 2 diabetes. Other models consisted of longitudinal linear mixed models including available data collected during follow-up.

^b^ Indicates number of participants contributing to analysis.

^c^ Holm-Bonferroni method was not applied given that no *P* values were statistically significant.

### Primary Outcome in Prespecified Subgroups

The effects of the lifestyle intervention on the primary outcome of worsening glycemic status were broadly consistent across prespecified subgroups. There was no statistically significant heterogeneity of intervention effect by subgroup (eFigure 6 in Supplement 2).

## Discussion

In this randomized clinical trial, a 12-month pragmatic lifestyle intervention designed for relevance to local context, preferences, and resources did not prevent deterioration in glycemic status among women with recent GDM from urban centers in South Asia. Compared with usual care, the intervention did not influence changes in body weight, fasting plasma glucose, or other outcomes. The mode of intervention delivery was impacted by COVID-19 pandemic restrictions.

The major strengths of this study include the large sample size, particularly relative to the existing evidence base, and inclusion of participants from diverse settings. The trial design had broad inclusion and limited exclusion criteria, with good statistical power to identify moderate intervention effects on clinically relevant outcomes. By some margin, this is the largest trial to date that has reported the effects of lifestyle interventions among women with recent GDM, to our knowledge. A 2018 systematic review^10^ identified 15 randomized clinical trials of lifestyle interventions post-GDM, of which 8 studies (with 180 events) were included in meta-analysis of the effects on diabetes incidence. This did not show a clear reduction in diabetes incidence with lifestyle intervention (relative risk, 0.75 [95% CI, 0.55-1.03]) but suggested that interventions delivered within 12 months of childbirth may be more effective. A more recent meta-analysis included 11 studies (with 199 events among 1926 participants) by expanding search criteria to include Chinese-language journals. It found a relative risk of 0.66 (95% CI, 0.51-0.85) for development of diabetes with lifestyle interventions compared with usual care, all of which commenced within 3 years postpartum.^22^ With 154 incident diabetes events, our study would contribute 43% of all outcomes if combined with previous trials. However, important differences between trials, including eligibility criteria, risk profile of the study population, and nature of the intervention, are likely to limit interpretability of summary data.

Secondary data from the DPP study^23^ showed an association between weight loss and diabetes prevention, although this was among a cohort of women recruited many years after their GDM-affected pregnancy. Prior systematic reviews^10^ also reported that lifestyle interventions post-GDM were associated with decreases in body weight by approximately 1 kg. In the current study, a mean increase in body weight was observed in both groups, despite decreases in caloric intake. This may have been counterbalanced by decreased physical activity based on the limited data on which this could be assessed. The LIVING intervention was based on previous approaches that were associated with decreases in body weight^14,15^ but was modified through a careful process of formative research in participating countries to be scalable in the context of available resources and patient preferences.^16^ This may have resulted in an intervention that was simply inadequate, particularly in terms of the personnel involved, given that staff expertise was identified as an implementation characteristic associated with the greatest postpartum weight decrease in a 2019 systematic review.^24^ COVID-19 restrictions also impacted the mode of intervention delivery in our study, with a substantial proportion of face-to-face group sessions modified to remotely -delivered individual sessions. Loss of in-person group interaction dynamics may have impacted effectiveness.^25^ Furthermore, lockdown restrictions may have decreased opportunities to increase physical activity and adapt diet.^26^ A mixed-methods process evaluation^27^ may provide more insights into the potential reasons for the lack of intervention effectiveness.

An important finding in our study was the high incidence of early postpartum dysglycemia, with 8.8% and 34.1% presenting for a prerandomization OGTT having type 2 diabetes and prediabetes, respectively, at a median of 6.9 months postpartum. Of individuals with prediabetes at baseline, 20.3% developed type 2 diabetes during follow-up, compared with 3.2% among those with baseline normoglycemia, identifying a particularly high-risk group. Given such high levels of risk, study outcomes suggest a compelling need to further investigate approaches to prevention, including pharmacotherapy. While existing data for pharmacotherapy in this context are promising, these are currently limited and have been insufficient to influence practice.^28,29^

### Limitations

This study has several limitations, including the participant-unblinded design. However laboratory request forms did not include information on participant allocation, and an independent observer verified all outcome data in the case record form against original laboratory results. Biased self-reporting on diet and physical activity may have affected interpretation of these outcomes. Intervention facilitators were not involved in trial follow-up, and study officers were not involved in intervention delivery. While a central laboratory was not used, randomization was stratified by center; thus, systematic differences among laboratories may not have introduced bias. The trial was limited to hospitals in urban settings, and the findings may not be broadly generalizable even within South Asia, especially to nonurban areas in these countries. Some data derived from the questionnaire on physical activity were found to be unreliable, raising questions about validity of the instrument in this population and these settings.^30^ Additionally, COVID-19 restrictions impacted outcome ascertainment among some participants, although not differentially between groups and not to the extent that study power was compromised.

## Conclusions

This study found that a pragmatic lifestyle intervention did not prevent glycemic deterioration among women with recent GDM at urban centers in South Asia. Most women who were assessed developed a deterioration of glycemia during follow-up before or after randomization, and those with prediabetes represented a particularly high-risk group for the development of diabetes. These findings suggest that additional strategies, including preventive drug therapies, should be considered for further research in this group.

## Notes

Supplement 1.
Trial Protocol

Supplement 2. **eMethods 1.** Methods for Diagnosis of Gestational Diabetes **eMethods 2.** Detailed Description of Planned Lifestyle Intervention Program **eTable 1.** Baseline Characteristics by Country **eTable 2.** Participant Disposition by Visit **eTable 3.** Sensitivity Analyses for Primary Outcome **eTable 4.** Effects of Intervention on Additional Outcomes **eFigure 1.** Primary Outcome Determination for Sensitivity Analysis **eFigure 2.** Postdelivery Glycemic Status of Registered Participants **eFigure 3.** Intervention Fidelity **eFigure 4.** Changes In Continuous Secondary Outcomes Over Time **eFigure 5.** Kaplan-Meier Plot for Development of Type 2 Diabetes **eFigure 6.** Prespecified Subgroup Analyses for Primary Outcome

Supplement 3.
Group Authors

Supplement 4.
Data Sharing Statement
