# Supplementary material for: Effects of a Lifestyle Intervention to Prevent Deterioration in Glycemic Status Among South Asian Women With Recent Gestational Diabetes: A Randomized Clinical Trial
Source: JAMA Netw Open. 2022 Mar 2;5(3):e220773. doi: 10.1001/jamanetworkopen.2022.0773 (PMC8892226; doi:10.1001/jamanetworkopen.2022.0773)
Supplement: Supplement 1. — Trial Protocol [file jamanetwopen-e220773-s001.pdf]

Lifestyle **I**nter**V**ention **IN** **G**estational Diabetes (LIVING)

A lifestyle intervention program for the prevention of type 2 diabetes mellitus among South Asian women with gestational diabetes mellitus

Version 6.0 dated May 13, 2020

PRIVILEGED AND CONFIDENTIAL

Not for distribution beyond intended function

Table of Contents

1. Administrative information 4
1.1 Trial/Study Registration 4
1.2 Funding 4
1.3 Roles and Responsibilities 4
1.3.1 Study Sponsors 4
1.3.2 Steering Committee 4
1.3.3 Central Coordinating Centre at AIIMS 5
1.3.4 India (North) Regional Coordinating Centre 5
1.3.5 India (South) Regional Coordinating Centre 5
1.3.6 Bangladesh Coordinating Centre 5
1.3.7 Sri Lanka Coordinating Centre 5
1.3.8 Statistics and Data Management Centre 6
1.4 Glossary of abbreviations and terms 7
1.5 Protocol Synopsis 8
2. Introduction 10
2.1 Background and Rationale 10
2.2 Aim 14
2.3 Study Design 14
3. Methods: Participants, interventions, outcomes, and evaluation 14
3.1 Study Setting 14
3.2 Eligibility Criteria 14
3.3 Study arms 17
3.3.1 Intervention 17
3.3.2 Control 19
3.4 Outcomes 19
3.5 Sample size 19
3.6 Statistical methods 20
3.7 Process evaluation 20
3.8 Economic evaluation 20
4. Methods: Assignment of interventions 21
4.1 Allocation 21
4.2 Blinding 21
5. Methods: Data collection, management, and analysis 21
5.1 Data collection methods 21
5.1.1 Pre-registration visit (24-34 weeks gestation) 22
5.1.2 Registration visit (3-18 months post-partum) 22
5.1.3 Randomisation visit (Week 0) 22
5.1.4 Six month visits (Month 6, 12, 18, 24, 30, and 36) 23
5.1.5 EOS visit (End of study) 23
5.2 Data Management 23
5.3 Outcome Adjudication 23
6. Methods: Monitoring and Safety 23
6.1 Serious Adverse Events 23
6.2 Monitoring 24
7. Ethics and confidentiality 24
7.1 Research Ethics Approval 24
7.2 Consent 25
7.3 Confidentiality 25
8. Administrative section 25
8.1 Steering Committee 25
8.2 Insurance 25
8.3 Record retention 25
8.4 Ownership, Disclosure of Data, and Publication 25
8.5 Funding 26

9.REFERENCES 26
10. ANNEXURES 28
10.1 Annexure 1 – Formative stage protocol V1.3 28
10.2 Annexure 2 - PISCF for Stage 1 consent – Main trial V2.0 34
10.3 Annexure 3 – PISCF for Stage 2 consent – Main trial V4.0 42
10.4 Annexure 4 - PISCF for Formative Stage V1.3 51
10.5 Annexure 5 – Protocol for Process Evaluation V 1.0 72
10.6 Annexure 6 - Protocol Signature Page 100

1. ADMINISTRATIVE INFORMATION

1.1 TRIAL/STUDY REGISTRATION

The study is registered on the Clinical Trials Registry of India (http://ctri.nic.in) and the Sri Lanka Clinical Trials Registry (http://slctr.lk).

1.2 FUNDING

The study is funded by a National Health and Medical Research Council of Australia (NHMRC) Global Alliance for Chronic Diseases (GACD) Grant (APP1093171) and Indian Council of Medical Research (ICMR) GACD Grant. The NHMRC and ICMR do not / will not have any role in the study design, management, data analysis and interpretation, writing of the report, or the decision to submit the report for publication.

1.3 Roles and Responsibilities

1.3.1 Study Sponsors

The George Institute for Global Health<br>Level 5, 1 King street Newtown NSW, Australia 2042<br>PO Box M201<br>Missenden Road<br>NSW 2050<br>Australia	Department of Endocrinology and Metabolism<br>Biotechnology Block<br>All India Institute of Medical Sciences<br>Ansari Nagar, New Delhi - 110029
Co-sponsor as Administrating Institution of funding from NHMRC	Co-sponsor as Administrating Institution of funding from ICMR

1.3.2 Steering Committee

Professor Nikhil Tandon<br>(PI and co-Chair)<br>All India Institute of Medical Sciences<br>New Delhi, India	Professor Anushka Patel<br>(PI and co-Chair)<br>The George Institute for Global Health<br>Sydney, Australia
Dr. Dewan Alam<br>Centre for Global Health Research, Toronto, Canada	Professor Neerja Bhatla<br>All India Institute of Medical Sciences, New Delhi, India
Associate Professor Laurent Billot<br>The George Institute for Global Health, Sydney, Australia	Dr. Ankush Desai<br>Goa Medical College & Hospital<br>Goa, India
Professor Asita de Silva<br>University of Kelaniya<br>Sri Lanka	Dr. Hema Divakar<br>Federation of Obstetrics and Gynaecology Societies of India
Dr. Sunil Fernando<br>University of Kelaniya<br>Colombo, Sri Lanka	Professor Stephen Jan<br>The George Institute for Global Health<br>Sydney, Australia
Associate Professor Rohina Joshi<br>The George Institute for Global Health, Sydney, Australia	
Dr. Aliya Naheed<br>icddr,b<br>Dhaka, Bangladesh	Professor Arunasalam Pathmeswaran<br>University of Kelaniya<br>Sri Lanka
Professor D. Prabhakaran<br>Centre for Chronic Disease Control<br>New Delhi, India	Dr. D. Praveen<br>George Institute of Global Health<br>Hyderabad, India
Professor Helena Teede<br>Monash University	Professor Sophia Zoungas<br>Monash University,

Melbourne, Australia	Melbourne, Australia

1.3.3 Central Coordinating Centre at AIIMS

Professor Nikhil Tandon<br>All India Institute of Medical Sciences<br>New Delhi, India
Dr. Yashdeep Gupta<br>All India Institute of Medical Sciences<br>New Delhi, India
Ms. Deksha Kapoor<br>All India Institute of Medical Sciences<br>New Delhi, India
Ms. Anu Kaushik<br>All India Institute of Medical Sciences<br>New Delhi, India
Ms. Neha Sethi<br>All India Institute of Medical Sciences<br>New Delhi, India

1.3.4 India (North) Regional Coordinating Centre

Professor D. Prabhakaran<br>Centre for Chronic Disease Control<br>New Delhi, India	Dr. Ishita Rawal<br>Centre for Chronic Disease Control<br>New Delhi, India
Ms. Ishita Agarwal<br>Centre for Chronic Disease Control<br>New Delhi, India	

1.3.5 India (South) Regional Coordinating Centre

Dr. D. Praveen<br>The George Institute for Global Health<br>Hyderabad, India	Dr. Josyula K. Lakshmi<br>The George Institute for Global Health<br>Hyderabad, India
Dr. Renu John<br>The George Institute for Global Health<br>Hyderabad, India	Dr. Purnima Rao Jevaji,<br>The George Institute for Global Health<br>Hyderabad, India

1.3.6 Bangladesh Coordinating Centre

Dr. Aliya Naheed<br>icddr,b<br>Dhaka, Bangladesh
Dr. Noshin Farzana<br>icddr,b<br>Dhaka, Bangladesh
Mr. Nantu Chakma<br>icddr,b<br>Dhaka, Bangladesh

1.3.7 Sri Lanka Coordinating Centre

Professor Asita de Silva<br>University of Kelaniya<br>Sri Lanka	Professor Arunasalam Pathmeswaran<br>University of Kelaniya<br>Sri Lanka
Mr. Thanushanthan. J<br>Project Lead<br>Sri Lanka	Mr. Shehan Gnanapragasam<br>Assistant Project Lead<br>Sri Lanka
Ms. Saumiyah Ajanthan	

Research Coordinator<br>Sri Lanka	

1.3.8 Statistics and Data Management Centre

Associate Professor Laurent Billot<br>The George Institute for Global Health<br>Sydney, Australia	Mr Paul Donnelly<br>George Clinical<br>Sydney, Australia

1.3.9 Consultant - Project Management

Ms. Helen Monaghan<br>Director, Global Project Operations,<br>Centre for Operational and Research Excellence,<br>The George Institute for Global Health,<br>Sydney, Australia

1.4 GLOSSARY OF ABBREVIATIONS AND TERMS

ADA	American Diabetes Association
D-CLIP	Diabetes Community Lifestyle Improvement Program
FPG	Fasting Plasma Glucose
GDM	Gestational Diabetes Mellitus
HeLP-her	Healthy Lifestyle Program for her
IADPSG	International Association of Diabetes and Pregnancy Study Groups
IDDP	Indian Diabetes Prevention Program
IGT	Impaired Glucose Tolerance
IFG	Impaired Fasting Glucose
NGT	Normal Glucose Tolerance
OGTT	Oral Glucose Tolerance Test
QALY	Quality Adjusted Life Year
RCT	Randomised Controlled Trial
SAE	Serious Adverse Event
T2DM	Type 2 Diabetes Mellitus
USDPP	United States Diabetes Prevention Program

1.5 PROTOCOL SYNOPSIS

**Title**	A lifestyle intervention program for the prevention of type 2 diabetes mellitus among South Asian women with gestational diabetes mellitus.
**Rationale**	Women with gestational diabetes mellitus (GDM) are at high risk of subsequent development of type 2 diabetes mellitus (T2DM), particularly in South Asia, where the prevalence of GDM is rising sharply. It is uncertain whether a behavioural modification program tailored towards young women changing risk-related behaviour will prevent or delay the onset of T2DM.
**Design**	An open-label parallel group pragmatic individual randomised controlled trial with blinded primary endpoint adjudication. The randomised controlled trial will be preceded by an intervention development and optimisation phase.
**Intervention**	Low intensity lifestyle modification program
**Number of participants**	1414 women with prior GDM from a total of 24 hospitals in Bangladesh, India and Sri Lanka.
**Duration**	60 months
**Outcomes**	*Primary outcomes:*<br>Proportion of women with a change of glycaemic category, at or prior to final visit:<br>• Normal glucose tolerance to impaired fasting glucose (IFG) or impaired glucose tolerance (IGT) or T2DM;<br>• IFG or IGT to T2DM<br><br>*Secondary outcomes:*<br>Body weight, waist circumference, systolic blood pressure, fasting blood glucose, physical activity level and diet
**Inclusion criteria**	Absence of T2DM at 3 to 18 months post-partum OGTT
**Exclusion criteria**	• Confirmed case of Type 2 Diabetes<br>• Travel time to hospital more than 2 hours (unless individual circumstances will not impede hospital attendance for study visits)<br>• Lack of access to a mobile telephone<br>• Use of steroids during pregnancy other than for lung maturation of the baby<br>• Likelihood of moving residence in the next 3 years
**Study schedule**	Jan 2016-Jan 2021
**Statistical considerations**	Sample size calculation – 1414 women with GDM from 24 hospitals (~60/hospital) will provide approximately 90% power (2α=0.05) to detect a relative risk of ≤0.65, assuming the control group cumulative incidence of change in glycaemic category will be at least 20% (median follow-up 24 months), and allowing for 20% drop out from trial (data) follow up. A key secondary outcome is body weight; inclusion of 1414 women will provide 90% power to detect a difference of 1.80kg (assuming mean body weight of 64.2kg [sd 10.4]) in the control group.
**Data management**	Data management will be the responsibility of The George Institute for Global Health. An electronic Case Record Form and an appropriate electronic data management system will be utilised to capture and store study data.

Figure 1. Study Schema

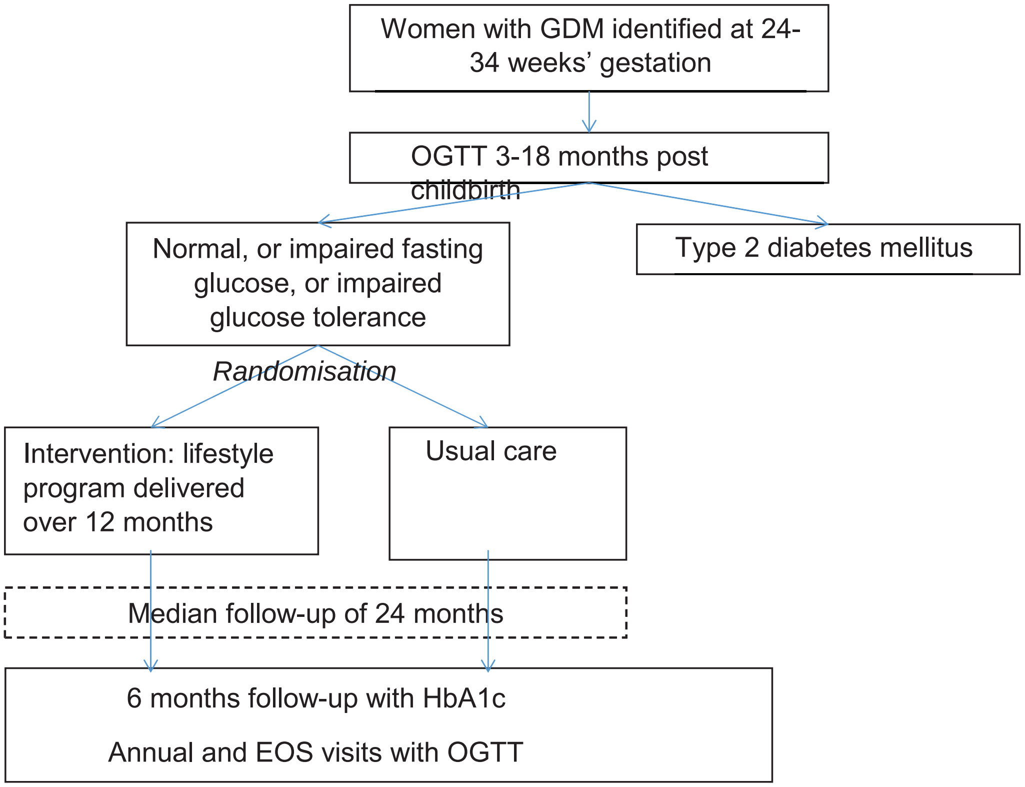

2. INTRODUCTION

2.1 BACKGROUND AND RATIONALE

Prevalence of GDM and conversion to T2DM in South Asian women
Around 25% of the 25 million pregnancies among Indian women each year are associated with gestational diabetes mellitus (GDM), and this proportion appears to be rapidly increasing over time [1-5]. In one report, somewhat lower prevalence rates for GDM for Sri Lanka (13%) and Bangladesh (10%) have been described [5]. Other data indicate higher GDM prevalence in Sri Lanka [6], and while relevant data are limited in Bangladesh, population trends of type 2 diabetes mellitus (T2DM) in urban communities are very similar to those in India [7]. Prior GDM is an established risk factor for the future development of T2DM, with a large systematic review showing an estimated 7-fold greater risk in women with GDM compared to those with normoglycaemic pregnancies [8]. In the US Diabetes Prevention Program (DPP), women with prior GDM had a substantially higher incidence of T2DM compared with other pre-diabetic parous women (15.2 vs. 8.9 per 100 person-years) [9]. Indian data suggest that 35-40% of women with GDM will develop T2DM within 5 years of delivery [3], more than double the rate observed in populations of largely white ethnicity [8].

Recent data have been obtained through a pilot study, PregDiabCare, in 2 urban Indian hospitals, funded by the International Diabetes Federation Bridges program [10]. Phase 1 of this pilot study aimed to provide contemporary data on the incidence of dysglycaemia after delivery in women with GDM. Among 366 women with prior GDM screened at a mean of 20 months post-delivery, 40% had impaired glucose tolerance (IGT) and/or impaired fasting glucose (IFG) and 32% had T2DM – *all new diagnoses*. Only 28% had normal glucose tolerance (NGT). A proportion of those with T2DM may have had this condition pre-pregnancy but undiagnosed, however this is likely to be a very small fraction given the estimated prevalence of T2DM in the age-matched general population of <5% [11].

Lifestyle interventions to prevent development of T2DM
Previous randomised controlled trials (RCT) have demonstrated that lifestyle modification programs directed at individuals with pre-diabetes can prevent or delay the onset of T2DM [12-17]. The DPP lifestyle intervention, conducted in a multi-ethnic US population was highly effective, with a number needed to treat of only 7 over 3 years [13]. In South Asia, the Indian Diabetes Prevention Programme (IDDP)[15] showed that in a mostly male, middle-aged Indian population with persistent IGT, a low intensity lifestyle modification was associated with a one-third reduction in T2DM incidence, over a median follow-up of 30 months. The 3-year cumulative incidence of T2DM in the control group of this study was 55%, so while the relative effects of the intervention in IDDP were smaller than those observed in DPP, the absolute reduction in development of T2DM was greater by virtue of the high baseline risk [13, 15].

Lifestyle interventions to prevent development of T2DM in women with prior GDM
To date, there are no completed adequately-powered trials of lifestyle interventions for T2DM prevention in women with prior GDM. In the cohort of 351 women with prior GDM included in DPP, the lifestyle intervention appeared to be more effective in preventing T2DM in comparison to parous pre-diabetic women without a history of GDM (50% vs 14% relative risk reduction) [9]. [9]. In populations outside of South Asia, data on the effectiveness of lifestyle interventions for diabetes prevention, specifically in women with prior GDM, are sparse. A recent cluster RCT involving 2320 women in the US [18] demonstrated that a DPP-derived, print/telephone-based lifestyle intervention was associated with modest reductions in postpartum weight retention [19]. Interim results of an individual RCT in China among 1180 women [20] are supportive of the effectiveness and feasibility of a lifestyle intervention program. In India, the Diabetes Community Lifestyle Improvement Program (D-CLIP) is currently evaluating a DPP-modified intervention in an RCT of approximately 600 men and women in India with dysglycaemia [21]. Less than 6% of D-CLIP participants have a history of GDM, and, to the best of our knowledge, there are no studies in South Asia among women with prior GDM, focused on T2DM prevention.

Developing an appropriate lifestyle intervention for South Asian women with GDM

In the absence of any proven lifestyle strategy for T2DM prevention among women with prior GDM in South Asian countries, we have drawn on four programs in developing the intervention proposed here. The first two are the DPP [13] and IDPP [15] studies (described above). The third is the intervention used in Phase 2 of PregDiabCare, which aimed to provide preliminary evidence about the feasibility and acceptability of a lifestyle program in Indian women with prior GDM [10]. The last is the Healthy Lifestyle Program for her (HeLP-her) [22], a highly successful program focused on young women, tested in a variety of populations and settings, and now implemented by the government in Australia. A description of each of these programs, their evaluations, and key learnings relevant to the current study are described in **Table 1**.

A crucial component of any lifestyle intervention is its delivery by appropriately trained staff. While wealthier countries are able to utilise skilled and often highly qualified healthcare workers, such resources are severely limited in low- and middle-income countries. One strategy to address this is “task-shifting” – a situation where a task normally performed by a highly trained healthcare worker is transferred to one with a different or lower level of training, and/or without formal health education [23]. In low- and middle-income countries, task-shifting or task-sharing has been embraced as a strategy to address chronic shortfalls in numbers of highly trained health professionals for the management of infectious and maternal and childhood illness [24, 25]. Increasingly, there is interest in such strategies in the context of chronic non-communicable diseases. While the evidence base for effectiveness in this context is currently limited, a recent systematic review of task-shifting in low and middle-income countries for the prevention or control of chronic diseases found that robust initial and on-going training, and use of algorithms or protocols to support decision-making were key facilitators of success [26]. We will incorporate this evidence in developing a training package for non-physician healthcare workers to deliver the new lifestyle intervention.

Table 1 - Relevant lifestyle change programs informing the new proposed intervention

	**DPP[13]**	**IDPP[15]**	**PregDiabCare(10)**	**HeLP-her[22]**	**HeLP-high risk pregnancy[27]**
Aim	Prevent type 2 DM	Prevent type 2 DM	Prevent type 2 DM (pilot feasibility only)	Prevent weight gain	Prevent excess gestational weight gain.
Setting & Context	Adults at high risk for the development of type 2 diabetes in the United States	Community setting in native Asian Indians		Community setting in urban Australian population	Community setting in rural Australian population
Evidence	RCT, 3234 pre-diabetic, 2.8 years.	RCT, 531 pre-diabetic, 2.5 years.	Uncontrolled, 58 women, 9 months.	RCT, 250 women community based (21% born overseas), 1 year	RCT, 228 pregnant high risk GDM, (60% Asian origin), 14 to 28 weeks gestation
Core elements	Prescriptive diet and physical activity, systematic and intensive	Increased physical activity and energy restriction Walk 30 mins /day	Healthy eating and physical activity education. Behaviour change: goal setting, relapse based on self-regulation theory.	Self-management, non-prescriptive, simple eating and activity messages, small achievable changes to behaviour, based on self-determination theory.	Self-management, non-prescriptive, simple eating and activity messages, small achievable changes to behaviour based on self-determination theory.
Delivery	16 face-to-face individual + group, delivered by trained case workers. Extra individual, phone & education classes.	6 face-to-face individual + monthly phone call over 3 years, delivered by dietician.	7x 2hrs group delivered by educated health professional.	4 group + SMS texts, delivery by trained health professional. No specialists in nutrition, physical activity or psychology required.	4 individual + SMS texts, delivered by trained health professional. No specialist nutrition, physical activity or psychology knowledge required.
Results	58% (95% CI: 48-66%) relative reduction in the incidence of T2DM. 38% achieved	29% (95% CI: 21-37%) rel. reduction in T2DM. At 2 yrs small weight gain in intervention and larger weight gain in control.	2 kg reduction in body weight, 2cm reduction in waist circumference from baseline not statistically significant.	Intervention group lost 200gms, controls gained 810g over 1 year. Difference of 1.13kg. Better glucose and lipid profiles with intervention.	By 28 wk gestation weight gain significantly lower with intervention (0.9kg), P < 0.05. Postpartum weight retention was lower with intervention, P<0.05.

	weight loss goal. Wt loss average 5%.				
Learnings and potential for scale-up	Reduction in T2DM incidence with modest weight loss. Intensive, costly, high attrition, not feasible in all countries.	In South Asians benefits achievable through prevention of weight gain. Low intensity, feasible, lower attrition.	Culturally appropriate. 54% attended 7 group sessions, 75% attended 5 sessions. Workforce with relevant skills scarce.	Retention rate excellent. 14% loss to follow-up. Simple messages, easy delivery with simple training scale–up demonstrated. Successful delivery in community.	Recruiting from hospital clinics is feasible. Delivered in a hospital setting. Scale-up feasible and can be delivered as part of standard care with simple training.

Summary of proposed work - We will take the learnings from the programs outlined in Table 1 to develop a new lifestyle program that has a high probability of being feasible, acceptable, and cost-effective in the South Asian context for women with prior GDM. We will optimise this intervention using an iterative, systems-based, and user-centred approach. The intervention will be delivered by auxiliary nurse midwives or their equivalent in each participating hospital, representing a strategy of within-system task-shifting to augment scalability and sustainability. We will then evaluate this in a randomised controlled trial to determine whether it will reduce the incidence of T2DM and dysglycaemia, in a manner that is affordable, acceptable, and scalable. This project focuses on generating new knowledge on implementation of a preventive strategy embedded within existing health systems, using mixed-methods evaluation to inform cost-effectiveness, acceptability, and scalability. It represents a case study of "Integrated Innovation™" [28] incorporating a science component (a program based on behaviour change theory that supports a multi-level approach to prevention by combining individually targeted strategies with social support), a social component (an innovative workforce strategy), and a sustainability component (a systems perspective for integration with existing health system infrastructure).

2.2 AIM

To determine whether a resource- and culturally-appropriate lifestyle intervention program in South Asian countries, provided to women with gestational diabetes, after childbirth, will reduce the incidence of worsening of glycaemic status, in a manner that is affordable, acceptable, and scalable.

2.3 STUDY DESIGN

An open-label parallel group pragmatic individual randomised controlled trial with blinded primary endpoint adjudication will be performed. The randomised controlled trial will be preceded by an intervention development and optimisation phase.

3. METHODS: PARTICIPANTS, INTERVENTIONS, OUTCOMES, AND EVALUATION

3.1 STUDY SETTING

The study will be conducted in approximately 24 hospitals and their catchment communities in 3 countries (Bangladesh, India, and Sri Lanka).
This is an individual RCT in 1414 women from 24 hospitals in India (~700 participants from 12 hospitals), Bangladesh (~350 participants from 6 hospitals) and Sri Lanka (~350 participants from 6 hospitals). Design: A pragmatic prospective randomised open-label blinded outcome evaluation (PROBE) controlled trial, with mixed methods evaluation, will be conducted (Figure 1). Central computer-based randomisation will be stratified by hospital, and use of insulin during index pregnancy.

3.2 ELIGIBILITY CRITERIA

At each participating hospital, women with GDM will be identified at 24-34 weeks of gestation through the performance of a standard oral glucose tolerance test (OGTT) using the International Association of the Diabetes and Pregnancy Study Groups (IADPSG) criteria.[29] If a patient has undergone assessment of glycaemic status prior to 24 weeks' gestation, an OGTT must be performed to confirm the diagnosis between 24 and 34 weeks' gestation, *unless* she is treated with an oral hypoglycaemic drug and/or insulin before 24 weeks, and satisfies the IADPSG criteria for diagnosis of GDM. If only FPG was done at 24-34 weeks, then she will be categorized as GDM/overt diabetes/ normal on the basis of FPG value alone (see Figure 2).

Figure 2. Scenarios to identify women with GDM eligible for the LIVING study based on diagnostic tests

Scenario 1: OGTT at 24-34 weeks of gestation (ideal scenario)

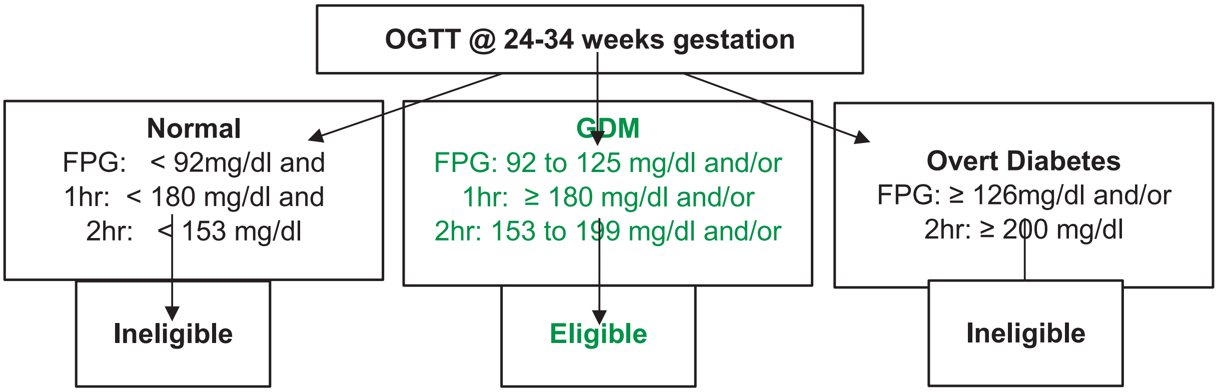

Scenario 2: OGTT at <24 weeks of gestation (scenario of second preference)

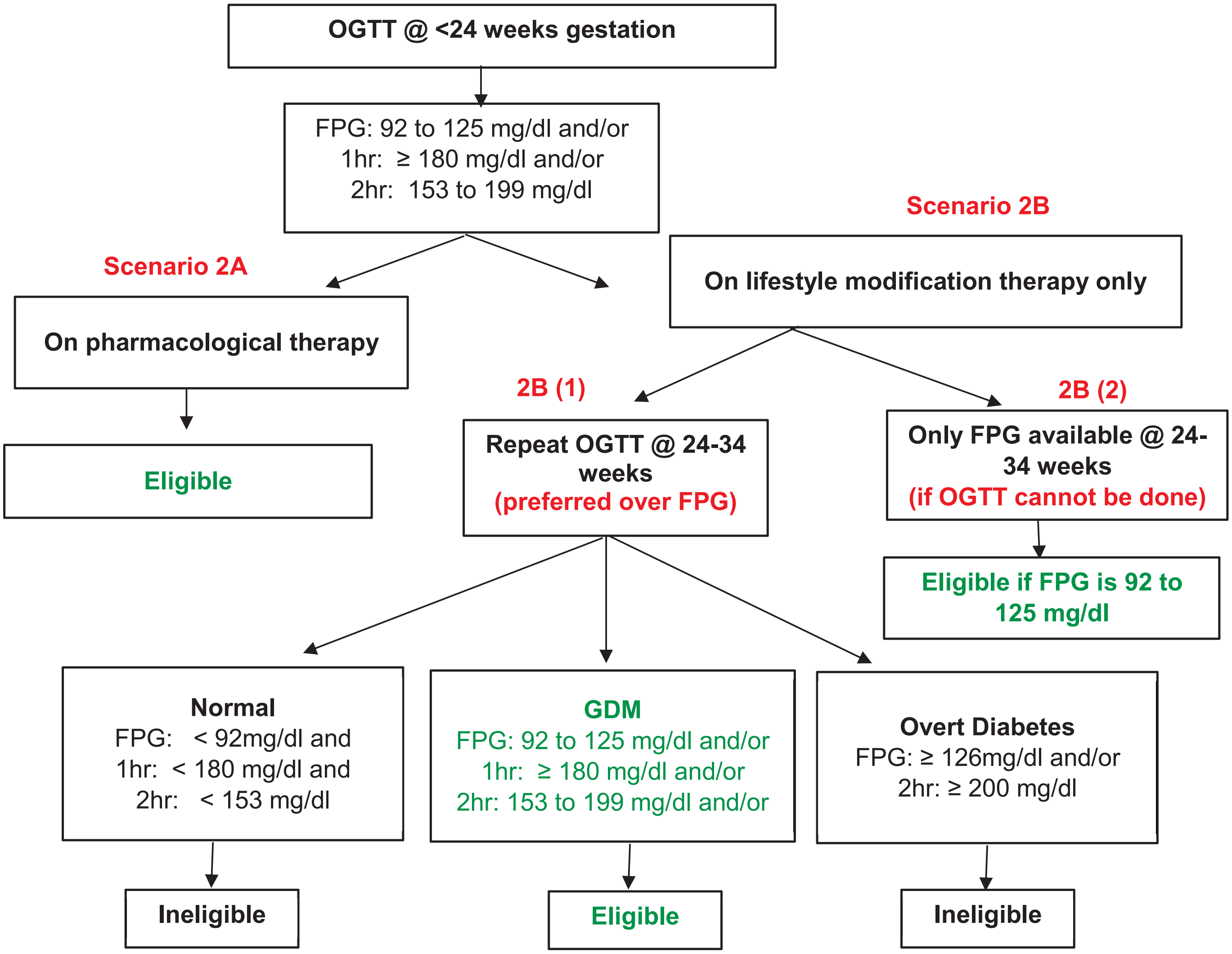

<u>**Scenario 3: FPG at <24 weeks of gestation (least favoured scenario)**</u>

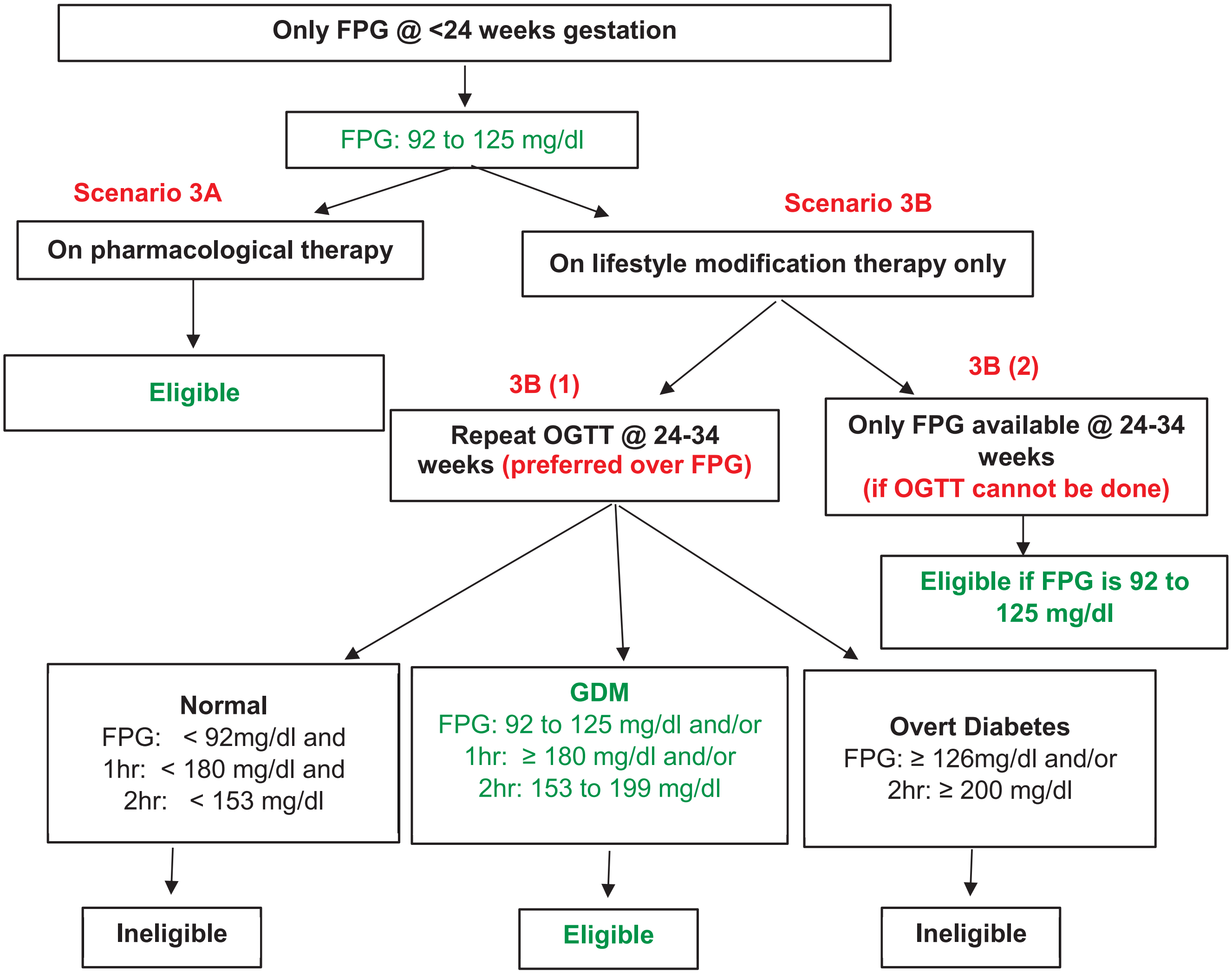

Abbreviations:

OGTT: Oral Glucose Tolerance Test
FPG: Fasting Plasma Glucose

These individuals will receive standard GDM care, but will also be exposed to an additional brief standardised educational program to improve knowledge of nutrition and physical activity both during and after pregnancy. The study will also be discussed, as establishing rapport with the participants at this stage will be important to maximise participation in post-pregnancy screening. Further contact will be made with potential participants on at least one occasion after hospital discharge, and prior to 3 months post-childbirth, when women will be invited for an OGTT to determine trial eligibility. Those diagnosed with T2DM using ADA criteria at this time will be referred for appropriate care; all other women will be eligible for trial participation.

The *inclusion* criterion, therefore, is the absence of T2DM (i.e. confirmation of NGT, IFG, or IGT) at 3 to 18 months post-partum OGTT. Consistent with design features of a pragmatic trial [29], *exclusion* criteria will be limited to:

- □ Confirmed case of Type 2 Diabetes

□ Travel time to hospital more than 2 hours (unless individual circumstances will not impede hospital attendance for study visits)
□ Lack of access to a mobile telephone
□ Use of steroids during pregnancy other than for lung maturation of the baby
□ Likelihood of moving residence in the next 3 years

All reasons for exclusion or consent refusal will be carefully recorded.

3.3 STUDY ARMS

3.3.1 Intervention

Provisionally, the intervention will include up to 4 face-to-face group sessions combined with remote on-going support, and an intensification offering when needed (**Table 2**). However, depending on outcomes during the optimisation phase, the balance of face-to-face and remote contact might vary substantially overall, and between countries. Face-to-face sessions will be delivered by a trained facilitator using the existing HeLP-her training program optimised for each country. Eligible and consenting women will attend a local hospital in groups of not more than 10 women, and have baseline measures assessed prior to the commencement of the program. During session 1, they will receive a program-specific user manual, and over the following weeks, work through the behavioural skills sessions with a health worker trained in the delivery of the program. The participant manuals developed for the PregDiabCare pilot will undergo minor adaptation for use in each country. Following the face-to-face visits, participants will receive reminders, motivational messages, and small actionable behaviours delivered using mobile text messages and/or voice messages, which we will adapt from the WHO BE HE@LTHY BE MOBILE manual on implementing m Diabetes [30]. . At week 26 from the commencement of the intervention, participants will undergo a review of progress. Program intensification will be offered to women who gain weight by the 6 month time point: those who gain >2% of baseline weight (which accounts for transient weight fluctuations) will receive two intensification sessions of individualised coaching, and monthly phone coaching for the remaining 6 months, delivered by the trained facilitators. All phases of the program focus on self-management through small, feasible, and sustainable changes, by building knowledge and skills across 3 themes (1) simple healthy eating, and moderate physical activity messages; 2) behavioural skills, such as problem-solving, goal-setting, and self-monitoring; and 3) enhancing internal motivation, self-efficacy, and self-management. Simple messages regarding eating and physical activity give women an achievable context in which to begin to formulate their own health priorities. The program is deliberately not prescriptive, and has a focus on sustainable behaviours and local context. It is estimated, based on the previous HeLP-her trials, that ~ 30% of participants may require more intensive support, as planned in the intensification sessions.

Table 2 - Outline of intervention design*

Time		Delivery	Content
24-34 weeks gestation		Individual	Introduce program: introduce study; general advice on T2DM risk; permission obtained to contact patient after childbirth and appointment made for post-partum OGTT.
Month 1-3	Session 1 Week 1	Group session (≤10 participants) (90 mins)	Introduce program and resources (manual and text messages); introduce concept of small steps to behaviour change and sensitize participants to importance of lifestyle modification. Ice breaking group activities.
	Session 2 Week 3	Group session	Emphasize on importance of diet for a healthy lifestyle and goal-setting to increase self-efficacy. Discussion on picture book. Group activities related to diet.

	Session 3<br>Week 6	Group session<br>+<br>*Support strategies delivered remotely*	Emphasize on physical activity for a healthy lifestyle and weight management, goal setting to increase self-efficacy, and discussion on household physical activities. Group activities related to physical activity.<br>*Following this session, participants receive reminders of core messages, behaviours, and actions via SMS or voice messages and phone calls*
Month 6	Session 4<br>Week 26	Group session<br>+<br>*Support strategies delivered remotely*	Review progress. Reinforcement of previous sessions, evaluation of goal-attainment and behaviour changes made. Group activity on reviewing core messages and personal goal-setting.<br>*Participants receive reminders of core messages, behaviours, and actions via SMS or voice messages and phone calls*
Month 7-12	2 Intensification Sessions (week 32 and week 44)	Individual sessions	If participants are persistently overweight, or gain >2% of baseline weight, intensification is offered based on barriers' assessment done earlier, and accordingly 2 counselling sessions with monthly phone coaching and SMSs are provided.
	Week 26 - 48	*Support strategies delivered remotely*	*Participants who lose the weight desired continue to receive reminders of core messages, behaviours, and actions via SMS and voice messages.*
	Week 52<br>End of intervention evaluation	Individual visit	Participants provide feedback on different elements of the intervention

*From March 25, 2020, due to lockdown measures relating to the Covid-19 emergency, all intervention sessions will be held remotely (telephone or video), either individually or in groups. As weight change cannot be assessed during lockdown conditions, all participants who complete Session 4 will be offered intensification.

Training

This intervention is an innovative adaptation of existing programs to young women post-childbirth, in resource-poor settings, who are at high risk of T2DM. Implementation strategies which maximise accessibility, engagement, uptake, and retention have been incorporated. Although the behavioural content is similar across all programs that have informed its development, the intervention is unique in how women are engaged and retained, and how the intervention is packaged to ensure less burden on participants, ease of delivery by minimally-trained healthcare workers, and low cost. To maximise accessibility and uptake, while minimising attrition and cost, face-to-face meetings are kept to a minimum, offering an intensity step-up only for those who need it. In addition, a range of technologies is employed to deliver follow-up support and information to participants. Program facilitators who have completed program-specific training will deliver the intervention. Facilitators will be selected from auxiliary nurse midwives (or equivalent) and will undergo two days of training. Facilitators need excellent interpersonal skills, sound knowledge of evidence-based practice, an understanding of health, health behaviour, nutrition, and physical activity, and knowledge and experience in communicating with a variety of participants. Program-specific training includes both theory and practical components, and motivational interviewing techniques. Following the initial training, facilitators will attend monthly meetings for ongoing support and opportunities to discuss barriers and develop strategies to optimise the delivery of the program. Facilitators will be provided with implementation protocols, data-collection templates, and delivery and implementation resources

such as scripts, food models, and photographs, and a selection of strategies to use to assist behaviour change.

Formative Phase
Prior to finalising the intervention, it will be optimised in a substantive formative phase as detailed in annexure 1. In each participating country, focus group discussions involving women with prior GDM will test the preliminary intervention design. In each participating country, a barriers analysis will be conducted to understand local contextual factors. This will be guided by Normalisation Process Theory [31] and Michie's behaviour change theories [29] to understand the capabilities, opportunities, and motivation of hospital administrators, existing auxiliary nurse midwives (or equivalent), and patients in relation to the intervention. In addition to desktop document analysis, process mapping work, semi-structured interviews, and focus group discussions with key stakeholders will be conducted. This will allow optimisation of the intervention for local delivery. This will also inform finalisation of the training program and material for the healthcare workers who will deliver the intervention (henceforth described as "facilitators"). All participants in the formative phase will be given information about the study and consent will be obtained (annexure 4).

3.3.2 Control
Control group participants will be referred to their usual doctor for ongoing management, with no attempt made to influence this. Any abnormal OGTT results during follow up will be provided to the patient and their doctor. This is entirely consistent with current usual care.

3.4 OUTCOMES

Primary outcome:

Proportion of women with a change of glycaemic category, at or prior to final visit:

- Normal glucose tolerance (NGT) to impaired fasting glucose (IFG) or impaired glucose tolerance (IGT), or T2DM;
- IFG or IGT to T2DM

This will be diagnosed using ADA criteria as given below:

- Normal glucose tolerance (<100 mg/dL fasting and <140 mg/dL post-glucose load)
- Impaired glucose tolerance (<100 mg/dL fasting and 140-199 mg/dL post-load)
- Impaired fasting glucose (100-125 mg/dL fasting and <140mg/dL post-load)
- Type 2 diabetes (≥126 mg/dL fasting or ≥200 mg/dL post-load)

If regional standards evolve to the diagnostic use of HbA1c with appropriate standardisation, we may migrate to this methodology depending on the timing and uptake of such changes.
Secondary outcomes:

- Fasting blood glucose
- Body weight
- Waist circumference
- Systolic blood pressure
- Physical activity level
- Diet

3.5 SAMPLE SIZE

The inclusion of 1414 women with GDM from 24 hospitals (~60/hospital) will provide 90% power ($2\alpha=0.05$) to detect a relative risk of ≤0.65, assuming that the control group cumulative incidence for change in glycaemic category will be 20%, and allowing for 20% drop out (from follow-up, not intervention). Assuming that no more than 5% of individuals with T2DM had pre-

existing undiagnosed pre-gestational T2DM, the PregDiabCare study results indicate an incidence of post-gestational T2DM of 27% at 20 months. Thus, with a median follow-up of 24 months, an assumed incidence of change in glycaemic category of 20% is conservative for the primary outcome and reasonable for the key secondary outcomes of T2DM.

3.6 STATISTICAL METHODS

Analysis will be based on the principle of intention-to-treat. The effectiveness of the study intervention on the primary outcomes will be determined using Cox models for survival data, with censoring of participants who develop a subsequent pregnancy during follow-up. Given that the occurrence of primary outcomes can only be registered to the date of ascertainment during study visits, the use of other methods (e.g. Poisson regression) for the primary analysis will be explored during the blind review and will be finalised in the Statistical Analysis Plan prior to database lock and unblinding. Analyses of secondary outcomes will be conducted using standard statistical procedures applicable to dichotomous, categorical or continuous data as appropriate.

3.7 PROCESS EVALUATION

A detailed process and outcome evaluation will be conducted to inform the successful implementation and scale-up of the intervention. It will also aim to inform for whom effectiveness was greatest and under what conditions or circumstances.

A detailed awareness of local contextual factors will be critical in the process evaluation. It will be informed by Normalisation Process Theory [31] assessing how well the new service fits within the processes of current service provision in the hospitals, and by Michie's behaviour change theories [29] to understand capabilities, opportunities and motivations of various actors and how they interact. The RE-AIM framework [32] will be adopted to inform translation of research findings to practice by considering both the individual and population impact of the intervention and sustainability of behaviour changes over time. Individual, contextual, ecological and system level outcomes will be assessed using qualitative methods. A maximum variation sampling technique will be employed to ensure that diverse opinions are gained from patients, healthcare workers (auxiliary nurse midwives or equivalent), hospital administrators, and representatives of relevant government agencies, with broad representation across the 3 countries. A number of process and outcome variables will be explored, including program delivery, fidelity, acceptability, cost, efficacy, and sustainability, that might influence scale up. Key issues to explore include (1) how healthcare workers deliver the intervention; (2) what effects it has on organisational practices and personnel, and vice versa; and (3) what are patients' experiences with the study, the factors that influence their motivation to take up and continue with recommended lifestyle changes, the strategies they use to do so, and the role of their family and peers. Data will be collected at the end of the intervention period through the use of semi-structured, in-depth interviews, and focus group discussions. All participants for the process evaluation will be given information about the study, and their consent obtained. A separate ethics approval/amendment will be sought before conducting process evaluation. Data will be analysed contemporaneously until thematic saturation is achieved. A multidisciplinary research team will guide the analysis process. See annexure 10.5 for the detailed protocol of the process evaluation, including the interview and discussion schedules, and participant information sheets and consent forms.

3.8 ECONOMIC EVALUATION

The economic evaluation will comprise a trial-based component and a modelled evaluation of long-term costs and outcomes. Intervention costs will be based on salaries and training. A within-trial comparison of health care costs (e.g. blood tests, hospital and clinic visits, and medications) will enable estimation of potential cost-offsets associated with the intervention.

As we do not expect an effect of the intervention on survival, the (within-trial-based) incremental cost effectiveness ratio will be determined by average differences in utility observed between treatment arms in the trial. To capture costs and outcomes beyond the trial, a decision-analytic model will be developed to enable long-term morbidity, quality of life and survival to be simulated. The model will specify a number of health states (such as myocardial infarction, stroke, renal failure, type 2 diabetes with and without micro and macro-vascular complications, death and no disease) between which individuals cycle each year over a lifetime. It will draw on the literature and available databases to determine the transition probabilities between health states and the cost and quality of life associated with each. All patients will simulate progression across states until death. Incremental cost per Quality Adjusted Life Year (QALY) gained will be determined by then folding back the model to determine the average costs and outcomes, discounted at appropriate rate, accrued in both treatment arms. Sensitivity analysis will be conducted to determine the robustness of base case estimates to assumptions used in the economic evaluation.

4. METHODS: ASSIGNMENT OF INTERVENTIONS

4.1 ALLOCATION

Randomisation will be conducted through a central, computer-based randomisation service, and will be stratified by country, centre, and use of insulin during pregnancy.

4.2 BLINDING

By necessity, neither site investigators nor participants will be blinded to allocation (intervention vs. usual care). However, all central study staff, statisticians, and outcome adjudicators will remain blinded until final database lock.

5. METHODS: DATA COLLECTION, MANAGEMENT, AND ANALYSIS

5.1 DATA COLLECTION METHODS

Follow-up data collection will operate on the principle of collecting the minimum information required to evaluate the major quantitative study outcomes. Data will be collected at randomisation and 6 monthly intervals thereafter, involving a case record form, with OGTT and HbA1c tests, alternately conducted at the follow-up 6-month visits, and both tests at the End of Study visit. Data will be captured electronically and data management will adhere to ICH-GCP guidelines, utilising Standard Operating Procedures established at participating institutes. Monitoring will be used to ensure quality and completeness of data, with an emphasis on central statistical monitoring to minimise costs. There will be 100% source data verification (against original laboratory reports) for all results from oral glucose tolerance tests, and of all HbA1c values.

Table 3 – Data collection

Measurements	Pre-Reg 24-34 weeks gestation	Reg 3-18 months post-partum	Rand	6 monthly visits	12 monthly visit	EOS visit
Written informed consent	X		X			
Eligibility criteria (inclusion/exclusion)	X	X	X			
Screen failure		X				

Participant demographics and medical history			X			
Anthropometric measurements			X	X	X	X
Oral glucose tolerance test (OGTT)	X	X		X$	X*	X*
HbA1c	X^	X		X		X
Vitals- BP and heart rate			X	X	X	X
Medical history			X			
Diet and physical activity questionnaires**			X	X	X	X
Healthcare utilisation (hospital, clinics, tests, medications)			X	X	X	X
Serious Adverse Events#			X	X	X	X

*OGTT will not be performed if interim diagnosis of T2DM is adequately confirmed by appropriate source documents
^HbA1c from old records (if available)
$ If HbA1c is ≥ 6.5 %, then an OGTT will be performed.
** Modified Global Physical Activity Questionnaire and Intake 24 Diet recall
# to be filled only when severe adverse event is seen any time after randomisation

From March 25, 2020, due to lockdown measures relating to the Covid-19 emergency, the following changes to participant follow-up have been instituted:

1. All follow-up visits with study participants will be conducted remotely via telephone. This will exclude the ability to collect any data relating to anthropometric measurements, OGTT, HbA1c and vitals (BP and heart rate).
2. All participants who have completed at least 12 months follow-up (and, if randomised to the intervention, have completed all aspects of the intervention) will have an EOS visit scheduled as their next visit.
3. Once lockdown conditions have been lifted, all participants will be scheduled for a visit to, at a minimum, undergo testing for HbA1c and undergo an OGTT. If conditions allow, in-clinic visits will be arranged for anthropometric measures and vitals (BP and heart rate).

5.1.1 Pre-registration visit (24-34 weeks gestation)

1. Obtain stage 1 written informed consent (annexure 2) to contact patient during the post-partum period and perform OGTT if not already performed as part of routine care.
2. If OGTT is positive, discuss interest in participation, obtain participant contact details, and deliver brief standardised educational program.

5.1.2 Registration visit (3-18 months post-partum)

1. Perform OGTT and measure HbA1c.
2. If OGTT indicates T2DM, refer for care; if OGTT does not indicate T2DM, invite patient to participate in trial, and arrange randomisation visit appointment (could be same day).

5.1.3 Randomisation visit (Week 0)

1. Obtain stage 2 written informed consent (annexure 3).
2. Assess inclusion and exclusion criteria.
3. Record demographic data and medical history.

4. Measure height, weight, waist circumference, and blood pressure.
5. Administer diet and physical activity questionnaires.
6. Record healthcare utilisation since delivery.

5.1.4 Six month visits (Month 6, 12, 18, 24, 30, and 36)

1. Measure weight, waist circumference, and blood pressure.
2. Determine whether an interim diagnosis of T2DM has been made.
3. If an interim diagnosis of T2DM has been made, obtain confirmatory source documentation. The participants will not be followed up further, and will be discontinued from the study intervention. Data will be censored for these participants.
4. At Months 6, 18, and 30, perform HbA1c. If value is more than 6.5%, then call patient for OGTT.
5. At Months 12, 24 and 36, perform OGTT, unless an interim diagnosis of T2DM has been made, and source documentation is available. Participants who change glycaemic category, but do not develop T2DM, will be followed up.
6. Administer diet and physical activity questionnaires.
7. Record healthcare utilisation since the last study visit.
8. Record any serious adverse events since the last study visit.

5.1.5 EOS visit (End of study)

1. Measure weight, waist circumference, and blood pressure.
2. Determine whether an interim diagnosis of T2DM has been made.
3. If an interim diagnosis of T2DM has been made, obtain confirmatory source documentation.
4. If an interim diagnosis of T2DM has not been made, perform OGTT and measure HbA1c.
5. Administer diet and physical activity questionnaires.
6. Record healthcare utilisation since the last study visit.
7. Record any serious adverse events since the last study visit.

5.2 DATA MANAGEMENT

Data management will be the responsibility of The George Institute for Global Health. An electronic Case Record Form and an appropriate electronic data management system will be utilised to capture and store study data. Data management will be responsible for generating automatic data queries and tracking their resolution, as well as generating study management reports. Only authorised users with appropriate permissions will have access to the database.

5.3 OUTCOME ADJUDICATION

As the primary outcome is entirely dependent on changes in absolute values from oral glucose tolerance tests (and does not involve any other non-laboratory criteria), independent outcome adjudication will not be required. To ensure the accuracy of relevant data, 100% source data verification against laboratory reports will be undertaken by appropriately qualified central study staff who are blinded to intervention allocation.

6. METHODS: MONITORING AND SAFETY

6.1 SERIOUS ADVERSE EVENTS

All serious adverse events must be reported during follow-up (from registration to 30 days after the final visit). A Serious Adverse Event (SAE) is any adverse event (any untoward, undesired, unplanned clinical event in the form of signs, symptoms, disease, or laboratory or physiological observations occurring in a human being participating in a clinical study, regardless of causal relationship) that meets one or more of the following criteria:

- results in death; or

- is life threatening (see below); or
- requires hospitalization or prolongation of an existing hospitalization (see below); or
- results in a persistent or significant disability or incapacity (see below); or
- results in a congenital anomaly or birth defect

Additionally, an important medical event that may not result in death, be life threatening, or require hospitalization may be considered an SAE when, based on appropriate medical judgment, they may jeopardize the subject and may require medical or surgical intervention to prevent one of the outcomes listed above. Examples of such events include allergic bronchospasm requiring intensive treatment in an emergency room or at home, blood dyscrasia or convulsions that do not require hospitalization, or development of drug dependency or drug abuse.

An adverse event is considered life threatening when this places the subject at immediate risk of death from the event as it occurred. A life-threatening event does not include an event that might have caused death had it occurred in a more severe form but that did not create an immediate risk of death as it actually occurred. For example, drug-induced hepatitis that resolved without evidence of hepatic failure would not be considered life threatening, even though drug-induced hepatitis of a more severe nature can be fatal. A hospitalization planned before the start of the study for a preexisting condition that has not worsened does not constitute an SAE (e.g. elective hospitalization for a cholecystectomy due to a preexisting condition of biliary colic that has not worsened during the study). Disability is defined as a substantial disruption in a person’s ability to conduct normal activities of daily living.

If there is any doubt whether an event constitutes an SAE, this event should be reported as an SAE.

Any SAE should be reported within 24 hours of the investigator’s or study coordinator’s becoming aware of this. Reporting of SAEs to Ethics Committees will comply with local requirements.

6.2 MONITORING

The Project Manager will devise a study monitoring plan which will be implemented by experienced monitors from each of the regional coordinating centres. Monitoring will take place to ensure compliance with the protocol GCP Principles and any local requirements.
From March 25, 2020, due to lockdown measures relating to the Covid-19 emergency, the monitoring plan has been adjusted to a remote format.

7. ETHICS AND CONFIDENTIALITY

This study will be designed, conducted, analysed and reported in accordance with ICH-GCP Principles and all local requirements in each participating country.

7.1 RESEARCH ETHICS APPROVAL

Ethics approval will be obtained from a number of ethics committees, including the University of Sydney Human Research Ethics Committee. In India, approvals from the Health Ministry Screening Committee and the All India Institute of Medical Sciences (Central Coordinating Centre) have been obtained. Further ethics committee approvals will be obtained from each hospital participating in India. In Sri Lanka, approval will be obtained from the Ethics Review Committee, Faculty of Medicine, University of Kelaniya, which is accredited by the Ministry of Health. In Bangladesh, ethics committee approval from icddr,b will be sufficient to allow involvement of all hospitals. The trial will be registered with the Clinical Trials Registry of India and the Sri Lanka Clinical Trials Registry before initiating the study.

7.2 CONSENT

Participants willing to take part in the study will be consented by trial centres as per the ethical requirements. A two-stage consent process will be utilised; with written informed consent being obtained both at initial engagement during pregnancy, as well as prior to randomisation. Participants will be given adequate explanation about the study and will be given ample time to consider their trial participation. They will be given the opportunity to ask questions about the trial and what their participation involves, and will receive clarification from the investigator and other study staff. Prior to a subject's participation in the trial, a written informed consent form (using appropriately translated versions where appropriate) should be signed and personally dated by the subject or by the subject's legally acceptable representative, and by the person who conducted the informed consent discussion. If a subject is unable to read or if a legally acceptable representative is unable to read, an impartial witness should be present during the entire informed consent discussion and must attest the written informed consent form. A copy of the signed written informed consent form will be given to the trial participant.

7.3 CONFIDENTIALITY

All documents and data relating to this study are strictly confidential. Documents given to the investigators and trial centres by the Coordinating Centres should not be disclosed to other parties without the written approval of the sponsors. The investigator and his or her team should maintain confidentiality of the identity of all study participants and assure security and confidentiality of study data and documents.

8. ADMINISTRATIVE SECTION

8.1 STEERING COMMITTEE

The Steering Committee will be the decision-making body for the trial. It will provide scientific direction to the study; approve the protocol and subsequent amendments; monitor study progress; and plan dissemination. The Steering Committee will meet through teleconference or other modes of communication at regular intervals to discuss study progress.

8.2 INSURANCE

In the event of a study related injury or death to a clinical trial participant, the sponsor(s) hold insurance policies to cover medical expenses and/or pay compensation in compliance with local ethical requirements.

8.3 RECORD RETENTION

All essential trial documents will be archived and retained at the trial centre and/or coordinating centres for at least 15 years after the completion of the study. At the end of such period, the investigator shall notify, in writing, the project management team of its intent to destroy all study material.

8.4 OWNERSHIP, DISCLOSURE OF DATA, AND PUBLICATION

The steering committee will have full ownership of the study data, its storage, and dissemination. All publications will be reviewed and approved by the steering committee which will be named on all reports. The research teams, collaborating investigators and their respective centres will be named, and trial participants acknowledged in the final report and in publications arising from the trial.

8.5 FUNDING

This study is funded by a National Health and Medical Research Council (Australia) grant, and an Indian Council of Medical Research grant provided under the Global Alliance for Chronic Disease scheme.

9.REFERENCES

1. Ramachandran, A., et al., *Prevalence of diabetes in pregnant women--a study from southern India.* Diabetes Res Clin Pract, 1994. **25**(1): p. 71-4.
2. Grewal, E., et al., *Prediction of gestational diabetes mellitus at 24 to 28 weeks of gestation by using first-trimester insulin sensitivity indices in Asian Indian subjects.* Metabolism, 2012. **61**(5): p. 715-20.
3. Seshiah, V., et al., *Gestational diabetes mellitus manifests in all trimesters of pregnancy.* Diabetes Res Clin Pract, 2007. **77**(3): p. 482-4.
4. Zargar, A.H., et al., *Prevalence of gestational diabetes mellitus in Kashmiri women from the Indian subcontinent.* Diabetes Res Clin Pract, 2004. **66**(2): p. 139-45.
5. Guariguata, L., et al., *Global estimates of the prevalence of hyperglycaemia in pregnancy.* Diabetes Res Clin Pract, 2014. **103**(2): p. 176-85.
6. Dahanayaka, N.J., et al., *Inadequacy of the risk factor based approach to detect gestational diabetes mellitus.* Ceylon Med J, 2012. **57**(1): p. 5-9.
7. Jayawardena, R., et al., *Prevalence and trends of the diabetes epidemic in South Asia: a systematic review and meta-analysis.* BMC Public Health, 2012. **12**: p. 380.
8. Bellamy, L., et al., *Type 2 diabetes mellitus after gestational diabetes: a systematic review and meta-analysis.* Lancet, 2009. **373**(9677): p. 1773-9.
9. Ratner, R.E., et al., *Prevention of diabetes in women with a history of gestational diabetes: effects of metformin and lifestyle interventions.* J Clin Endocrinol Metab, 2008. **93**(12): p. 4774-9.
10. Y. Gupta, D.K., A. Desai, D. Praveen, R. Joshi, R. Rozati, N. Bhatla, D. Prabhakaran, P. Reddy, A. Patel, N. Tandon, *The conversion of gestational diabetes mellitus to future type 2 diabetes mellitus and the predictive value of HbA1c in an Indian cohort.* Diabetic Medicine, 2016.
11. Ramachandran, A., et al., *High prevalence of diabetes and impaired glucose tolerance in India: National Urban Diabetes Survey.* Diabetologia, 2001. **44**(9): p. 1094-101.
12. Gillies, C.L., et al., *Pharmacological and lifestyle interventions to prevent or delay type 2 diabetes in people with impaired glucose tolerance: systematic review and meta-analysis.* BMJ, 2007. **334**(7588): p. 299.
13. Knowler, W.C., et al., *Reduction in the incidence of type 2 diabetes with lifestyle intervention or metformin.* N Engl J Med, 2002. **346**(6): p. 393-403.
14. Tuomilehto, J., et al., *Prevention of type 2 diabetes mellitus by changes in lifestyle among subjects with impaired glucose tolerance.* N Engl J Med, 2001. **344**(18): p. 1343-50.
15. Ramachandran, A., et al., *The Indian Diabetes Prevention Programme shows that lifestyle modification and metformin prevent type 2 diabetes in Asian Indian subjects with impaired glucose tolerance (IDPP-1).* Diabetologia, 2006. **49**(2): p. 289-97.
16. Pan, X.R., et al., *Effects of diet and exercise in preventing NIDDM in people with impaired glucose tolerance. The Da Qing IGT and Diabetes Study.* Diabetes Care, 1997. **20**(4): p. 537-44.
17. Dunkley, A.J., et al., *Diabetes prevention in the real world: effectiveness of pragmatic lifestyle interventions for the prevention of type 2 diabetes and of the impact of adherence to guideline recommendations: a systematic review and meta-analysis.* Diabetes Care, 2014. **37**(4): p. 922-33.
18. Ferrara, A., et al., *A pragmatic cluster randomized clinical trial of diabetes prevention strategies for women with gestational diabetes: design and rationale of the Gestational Diabetes' Effects on Moms (GEM) study.* BMC Pregnancy Childbirth, 2014. **14**: p. 21.

19. Ferrara, A., et al., *The Comparative Effectiveness of Diabetes Prevention Strategies to Reduce Postpartum Weight Retention in Women With Gestational Diabetes Mellitus: The Gestational Diabetes' Effects on Moms (GEM) Cluster Randomized Controlled Trial.* Diabetes Care, 2016. **39**(1): p. 65-74.
20. Hu, G., et al., *Tianjin Gestational Diabetes Mellitus Prevention Program: study design, methods, and 1-year interim report on the feasibility of lifestyle intervention program.* Diabetes Res Clin Pract, 2012. **98**(3): p. 508-17.
21. Weber, M.B., et al., *A model of translational research for diabetes prevention in low and middle-income countries: The Diabetes Community Lifestyle Improvement Program (D-CLIP) trial.* Prim Care Diabetes, 2012. **6**(1): p. 3-9.
22. Lombard, C., et al., *A low intensity, community based lifestyle programme to prevent weight gain in women with young children: cluster randomised controlled trial.* BMJ, 2010. **341**: p. c3215.
23. Lekoubou, A., et al., *Hypertension, diabetes mellitus and task shifting in their management in sub-Saharan Africa.* Int J Environ Res Public Health, 2010. **7**(2): p. 353-63.
24. Mdege, N.D., S. Chindove, and S. Ali, *The effectiveness and cost implications of task-shifting in the delivery of antiretroviral therapy to HIV-infected patients: a systematic review.* Health Policy Plan, 2013. **28**(3): p. 223-36.
25. Lewin, S., et al., *Lay health workers in primary and community health care for maternal and child health and the management of infectious diseases.* Cochrane Database Syst Rev, 2010(3): p. CD004015.
26. Joshi, R., et al., *Task shifting for non-communicable disease management in low and middle income countries--a systematic review.* PLoS One, 2014. **9**(8): p. e103754.
27. Harrison, C.L., et al., *Optimizing healthy gestational weight gain in women at high risk of gestational diabetes: a randomized controlled trial.* Obesity (Silver Spring), 2013. **21**(5): p. 904-9.
28. Grand Challenges Canada/ Grand Defis Canada. 2010 [cited 2014 27 May]; Available from: http://www.grandchallenges.ca/integrated-innovation/.
29. Michie, S., M.M. van Stralen, and R. West, *The behaviour change wheel: a new method for characterising and designing behaviour change interventions.* Implement Sci, 2011. **6**: p. 42.
30. A handbook on implementing m diabetes- WHO BE HE@LTHY BE MOBILE manual.
31. Murray, E., et al., *Normalisation process theory: a framework for developing, evaluating and implementing complex interventions.* BMC Med, 2010. **8**: p. 63.
32. Gaglio, B., J.A. Shoup, and R.E. Glasgow, *The RE-AIM framework: a systematic review of use over time.* Am J Public Health, 2013. **103**(6): p. e38-46.

10. ANNEXURES

10.1 ANNEXURE 1 – FORMATIVE STAGE PROTOCOL V1.3

Lifestyle InterVention IN Gestational Diabetes (LIVING)
A lifestyle intervention program for the prevention of type 2 diabetes mellitus among South Asian women with gestational diabetes mellitus

FORMATIVE STAGE PROTOCOL_V 1.3

Lifestyle InterVention IN Gestational Diabetes (LIVING)

Background

The aim of the LIVING study is to determine whether a resource and culturally appropriate lifestyle intervention program in South Asian countries (Bangladesh, India, Sri Lanka), provided to women with Gestational Diabetes Mellitus (GDM) after childbirth, will reduce the incidence of type 2 diabetes (T2DM) and dysglycaemia, in a manner that is affordable, acceptable and scalable. The intervention will be delivered by auxiliary nurse midwives or other relevant non-physician healthcare worker in each participating hospital, representing a strategy of within-system task-shifting to augment scalability and sustainability. We will then evaluate this in a Randomised Controlled Trial to determine whether it will reduce the incidence of T2DM and dysglycaemia, in a manner that is affordable, acceptable and scalable.

Overview of formative stage

Prior to the finalisation of the intervention, a qualitative formative process will be implemented to understand relevant local contextual factors (barriers and facilitators), behaviour of actors (capabilities, opportunities, motivations) and the health systems processes (actual and potential) for management of GDM and post-pregnancy care in the three countries. This will be guided by Normalisation Process Theory [1] and Michie's behaviour change theories[2] to understand the capabilities, opportunities and motivation of hospital administrators, existing nurse auxiliaries (or equivalent) and patients in relation to the intervention. In addition to desktop document analysis, process mapping work and semi-structured interviews, focus group discussions with key stakeholders will be conducted. This will allow optimisation of intervention for local delivery. This will also inform finalisation of the training program and material for the healthcare workers who will deliver the intervention (henceforth described as "facilitators").We will then optimise this intervention using an iterative, systems-based and user-centred approach. The formative stage research will be conducted in a total of 7 sites (4 in India, 2 in Bangladesh and 1 in Sri Lanka).

Objectives

The formative phase has 2 objectives:

	Objective	Method	Sample	Tool
1	To explore how health systems diagnose and manage gestational diabetes, content of the intervention and the best method to deliver the intervention using the existing health systems infrastructure and workforce.	In depth interviews (IDI) – 5 at each hospital [5*7=35 IDIs]	From each hospital one hospital administrator/ department administrator for Endocrinology or Obstetrics and Gynaecology/involved in resource allocation, one obstetrician, one endocrinologist, one non-physician health professional involved with obstetric or	IDI guide

			diabetes care (e.g. auxiliary nurse mid-wife or dietician) and post-partum women previously diagnosed with GDM in last 24 months .	
2	To refine the content and process of intervention delivery	Focus group discussion (FGD)	7 FGDs (one per hospital) with 6-8 women in each group. The focus groups will be comprised of post-partum women within 24 months of delivery who were previously diagnosed with GDM.	FGD guide

Methods

Recruitment strategy

A purposive sample of hospitals will be selected from study sites that have agreed to take part in the LIVING trial. One centre will be chosen in Sri Lanka, as this is considered sufficient to capture the likely diversity within the dominant health system. One public and one private hospital will be included from Bangladesh, North India and South India. Women with prior or current GDM will be purposively sampled from hospital records, with the objective of including some diversity related to sociodemographic background. These women will be contacted either by a phone call or during a visit to the hospital for routine care and written informed consent will be obtained from those who are willing to participate in the study. All interviews will be conducted by one or more trained social scientist(s).

Data Collection

Written informed consent will include permission to record the FGD/interview and the participants will be informed about respondent confidentiality and data usage. The project team will brief the participants on the purpose of the study. The discussion topics will be simple and easy to understand. Prompts and probes will be used to encourage the group to expand and broaden their discussions. Each FGD/interview would be conducted by a team of two, a moderator and a note-taker, and would last for approximately 45-90 minutes. All the questions listed in FGD guide and Interview schedule will be flexible and open ended. All the interviews will take place at the health centre and interviews will be audio-recorded.

Data management and Analysis

The recorded interviews will be transcribed verbatim and translated to English (where necessary), and will be stored in Microsoft word format. Access to the data will only be given to the researchers involved in the qualitative study and will be solely used for the purpose of research. The audio recordings will be analysed using emergent codes to develop themes. The principles of thematic analysis for key issues, recurring issues and commonly emerging topics, to identify patterns will be used.Each of these items will be initially coded, and a coding scheme developed for compiling and grouping information from the various interviews. The study findings will be presented in an anonymous way with participants' quotes in order to corroborate the interpretation of analysis[3]. The steps of coding and charting will be done using NVIVO (QSR International, Melbourne, Victoria), a qualitative data analysis software. A multidisciplinary research team will guide the entire analysis process.

Anticipated Outcome & Future Plans

FGDs and Interview data analyses will be utilised to inform the development of a culturally/contextually specific intervention for LIVING study.

Tools

<u>Objective 1</u> - To explore how health systems diagnose and manage gestational diabetes and the best method to deliver the intervention using the existing health system infrastructure and

workforce. This will be addressed through in-depth interviews of relevant healthcare professionals (endocrinologists/obstetricians/hospital administrators/other care providers such as auxiliary nurse midwives or dieticians) and with post-partum women previously diagnosed with GDM in last 24months.

In-depth-interview guide for healthcare professionals
Note: questions will vary according to type of healthcare professional.

1. **Introduction**
    a) Tell us about yourself (age/specialization/years of practice/etc.)
    b) Since how long you have been associated with this institution?
    c) How often do you see women with GDM?
    d) According to you, what are the challenges involved in managing the women with GDM?
2. **Current diagnosis and management of GDM**
    a) According to you, what are the current management and referral practices for GDM Patients?
    b) According to you, what are the current follow up procedures for GDM patients?
    c) In your opinion, is any behavioural change intervention is necessary for women with GDM following delivery? If yes what are they? And why?
    d) Are you aware of any existing support systems available in the hospital for such behavioural intervention for these women?
3. **Intervention (the interviewer to first explain the intervention and collect the following information)**
    a) Logistics of the intervention
        i. According to you, how could this intervention be delivered within the hospital if it were part of usual practice?
        ii. In your opinion, who would be the best health professional to deliver the intervention if it were to become usual practice?
        iii. In your opinion, what type of non-physician healthcare workers are available at the hospital who might have the ability to lead group sessions for women? Explore attitudes towards task-sharing.
        iv. According to you, do we need to provide incentives to participants attending the group sessions? If so, what type of incentives?
    b) What is your opinion on sustainability of the intervention, if the results of the study were to be positive?
    c) Logistics of the group sessions
        i. What do you think would be the ideal size of group for sessions?
        ii. What days / times of the week do you think would be most suitable to hold the session?
        iii. How easy will it be to find space for the sessions?
        iv. What are the ways to utilise the potential support from peers to encourage sustainable behaviour change?
        v. Do you think it would be feasible to create teams of 3-4 people within the group for internal monitoring of outcomes and enable teams to compare their performance against other teams?

In-depth-interview guide for post-partum women previously diagnosed with GDM in last 24 months
Introduction

a) Please tell us about yourself in brief? *Probe: age, literacy status, economic status, residence (urban / rural), type of family (nuclear / extended), occupation, number and gender of children, source of water, electrification, ownership of mobile phone, availability of cooking gas, availability of toilet facility at home, distance from the hospital availability of an escort to visit the hospital*.

1) **Current diagnosis and management of GDM**

   a) What do you know about Gestational Diabetes Mellitus?
   b) Please tell me about your experience of managing Gestational Diabetes?
   c) Generally, what kind of difficulties did you faced to manage your gestational diabetes?
   d) Can you tell me a little about the information you received from the hospital or the doctor? Do the discussion with doctors involves postpartum care also?
   e) Overall, how was your experience with the hospital staff?
   f) Did you visit the endocrinologist when you were diagnosed with GDM? please give reasons

2) **Postpartum screening**

   a) According to you, what are various barriers and facilitators that the women like you may face for returning for an OGTT in the post-partum period in hospital?

3) **Physical Activity & Healthy Diet**

   a) Do you think physical activity has an impact on health? In your opinion how important is to do the physical activity /exercise after child birth? (Probe; perception about post-partum weight gain), Give details?
   b) Have you done the physical activity/exercise after the birth of child? How much time did you spend on doing the exercise? (Probe: environmental or social barriers/ lack of motivation and facilitators like family support and attitude towards physical activity/weight gain)In your opinion what kind of activities (physical activities/ exercises) should be avoided after delivery?
   c) Nutritious food is required after the child birth. Please give your opinion? Generally, what type of food you have eaten after the child birth?
   d) Were you able to make decisions regarding your diet / food cooked at home after delivery? Are there any factors which have an impact on the mother's diet (Probe: potential barriers and facilitators- family structure ,family support, resources available, customs and rituals)

4) **Intervention**
   a) Do you think women with GDM/postpartum in your hospital need some information to bring awareness in the patients' outlook towards GDM & its treatment? If yes, please elaborate. If no, give reasons.
   b) According to you what are the different ways which we can adopt to engage these women for our program?
   c) Please suggest the best way you feel this intervention can be delivered (probe: at the hospital/mode of delivery- phone call, SMS /frequency of contact)
   d) Do you have any suggestions on how to keep women engaged with us in year 2 and 3?
   e) What are the ways to utilise the potential support from peer/family members to encourage sustainable behaviour change?
   f) Who do you think will be the best person to deliver this intervention if this was to become a usual practice, why do you feel so (probe: doctor/nurse/counsellor/MSW)
   g) What are the major challenges you expect women to encounter while this intervention is being delivered? Can you suggest measures to overcome these?
   h) Do you feel this intervention should be a part of the usual care for GDM?
   i) How big should be a group? Why do you think this is an appropriate size?
   j) How much time should one group session last?

k) Which days do you think are good for group sessions and what time do you think will be the best to conduct group sessions, why is it so?
l) What should be an appropriate place to conduct these sessions? Why do you suggest this place?
m) Do you have any more thoughts you would like to share with us? Do you have any questions for us?

Any additional comments to be sought.

Objective 2 - To refine the content and process of intervention delivery.This will be addressed through FGDs. Each focus group will include currently with or recently experienced (now post-partum) GDM. Each session is expected to last for 45-90 minutes.

FGD guide
After providing a brief introduction to the study and intervention, collect the following information:

a) Socio-demographic details - age, literacy status, economic status, residence (urban/rural), type of family (nuclear/extended), occupation, number of children, source of water, electrification, ownership of mobile phone, availability of cooking gas, availability of toilet facility at home and distance from the hospital.
b) At your home who is in control of kitchen i.e. who makes the decisions related to dietary intake?

Theme 1: Physical activity

a) Explore perceptions of post-partum physical activity.
b) Explore understanding of leisure time physical activity.
c) Explore beliefs about adequate physical activity.
d) Explore opportunities for exercise available and type of exercise available.
e) Understand levels of exercise through household activities and perceptions of these being sufficient.
c) Explore barriers to exercise (environmental or social barriers/lack of motivation/perceptions of post pregnancy weight gain).
d) Explore potential facilitators of exercise.

Theme 2: Healthy diet

a) Explore perceptions about healthy diet (relationship to weight gain, content).
b) Explore perceptions about post-partum diet (e.g. should be high in sugar and ghee).
c) Explore barriers to dietary change (e.g. who makes choices about family diet?).
d) Explore potential facilitators for dietary change.

Theme 3: Patient journey once diagnosed with GDM

a) Explore journey especially with respect to post-partum advice about future risks and how to manage these (including whether OGTT testing had been offered).
b) Explore post-partum interactions with delivery hospital and opportunities for re-engagement.
c) Explore concerns regarding costs or inconvenience that may be associated with post-partum care focused on mother's future health.

Theme 4: Postpartum screening

a) Explore facilitators and barriers for returning for an OGTT in the post-partum period.

Theme 5: Intervention

After providing a brief introduction to the intervention:

a) Explore reasons why women may or may not be interested in participating in the intervention.

b) Explore potential facilitators of attendance (e.g. timing with well -baby or family planning services, providing child care).
c) Explore preferences for size and timing of groups (day of week / time of day).
d) Explore barriers/challenges to attend group sessions at prescribed intervals.
e) Explore preferences for including family members / friends being involved in group sessions.
f) Explore preference for type of health professional delivering group sessions.
g) Explore preference for peer interactions.
h) Preference of remote support strategies– phone call/SMS/smartphone apps and any facilitators or barriers to remote support (e.g. literacy, infrequent phone access).

References

1. Murray E, Treweek S, Pope C, MacFarlane A, Ballini L, et al. (2010) Normalisation process theory: a framework for developing, evaluating and implementing complex interventions. BMC Med 8: 1.
2. Michie S, van Stralen MM, West R (2011) The behaviour change wheel: a new method for characterising and designing behaviour change interventions. Implementation Science 6: 42.
3. Ritchie J, Lewis J, Nicholls CM, Ormston R (2013) Qualitative research practice: A guide for social science students and researchers: Sage.

10.2 ANNEXURE 2 - PISCF FOR STAGE 1 CONSENT – MAIN TRIAL V2.0

Lifestyle **I**nter**V**ention **IN** **G**estational **D**iabetes (LIVING)
A lifestyle intervention program for the prevention of type 2 diabetes mellitus among South Asian women with gestational diabetes mellitus

PISCFs FOR STAGE 1 CONSENT_MAIN TRIAL_V 2.0

Study/Protocol Number-1093171

CHIEF INVESTIGATOR NAME
Prof. Anushka Patel

The George Institute for Global Health
Level 5, 1 King street Newtown NSW,
Australia 2042, PO Box M201, Missenden Road
NSW 2050,Australia

T +61 2 8052 4439 **F** +61 2 9993 4502
Email: apatel@georgeinstitute.org
Website: www.georgeinstitute.org

Lifestyle **I**nter**V**ention **IN** **G**estational Diabetes (LIVING)
STAGE 1 PARTICIPANT INFORMATION STATEMENT AND CONSENT FORM

(1) Why do I have to read this document?
You are invited to take part in a research study called LIVING (Lifestyle InterVention IN Gestational diabetes). This study will compare a low intensity post-delivery lifestyle program to usual care among women diagnosed with Gestational Diabetes Mellitus (GDM) during their recent pregnancy.

Before you decide to participate, it is important for you to understand why the research is being conducted, and what it will involve. Please take the time to read the following information carefully and if you wish, please discuss it with your doctor, other healthcare provider, relatives and friends. Your participation is voluntary and you are entirely free to decide whether or not to take part in this study. If you choose to take part, you are free to withdraw at any time, without giving any reason. If you choose not to take part, your medical care or legal rights will not be affected, and you will continue to receive standard care given by your doctors and other healthcare providers. If there is anything that is not clear, or if you would like to have more information, please do not hesitate to ask the doctor or the study team.
The trial doctor/research coordinator will discuss the study with you in detail. Once you understand the study and if you have no objections to your involvement, you will be asked to sign the consent form (last page of this document). By signing the consent form, you do not alter your legal rights, but you indicate that you understand the information and have no objection to participating in the study. You will be given a copy of this Participant Information Statement and Consent Form to keep for your records.
(2) What is this study about?
Gestational diabetes mellitus is a form of diabetes consisting of high blood glucose (sugar) levels during pregnancy. It develops in 4% pregnancies worldwide and is associated with complications to both mother and baby. In South Asia, the GDM rate is much higher and appears to be increasing over time. High blood glucose levels usually disappear after pregnancy, but women with GDM and their children are at an increased risk of developing type 2 diabetes later in life. Type 2 diabetes is a lifelong condition often requiring long term

medication use and associated with increased risks of heart disease, stroke, kidney disease, and eye disease. Studies from India suggest that 35-40% of women with GDM will develop type 2 diabetes within 5 years of delivery.

There is evidence that lifestyle modification programs can be useful in delaying the development of type 2 diabetes among other groups of people at high risk of developing this condition. However, it is not known whether a lifestyle modification program developed for women in South Asia with previous GDM can be successful is preventing the development of type 2 diabetes.

In this study, we will develop and test a new low intensity lifestyle program that has a high probability of being feasible, acceptable and affordable in the South Asian context for women with prior GDM.

(3) Who is running the study?

In India, the study is being conducted by the All India Institute of Medical Sciences (AIIMS), New Delhi and The George Institute for Global Health, Hyderabad. In Bangladesh the study is being conducted by International Centre for Diarrhoeal Disease Research, Bangladesh (icddr,b) , Dhaka and in Sri Lanka by the University of Kelaniya, Colombo. The study is funded by the Indian Council of Medical Research and the National Health and Medical Research Council of Australia and as a part of The Global Alliance for Chronic Diseases scheme.

The study is being carried out by following researchers:

**Professor Nikhil Tandon**<br>(PI and co-Chair)<br>All India Institute of Medical Sciences<br>New Delhi, India	**Professor Anushka Patel**<br>(PI and co-Chair)<br>The George Institute for Global Health<br>Sydney, Australia

Details of local researchers:

**India-North**<br>**Dr. Prabhakaran Dorairaj**<br>Centre for Chronic Disease Control (CCDC), C-1/52, Second Floor, Safdarjung Development Area (SDA), New Delhi -110016<br>Tel: 011-41618456 Email: dprabhakaran@ccdcindia.org	**India-South**<br>**Dr. Praveen Devarsetty**<br>The George Institute for Global Health, India<br>Unit No. 301, Second Floor, ANR Centre, Road No.1, Banjara Hills | Hyderabad - 500 034 | Telangana India<br>Ph: +91 40 3099 4444, Mobile: +91 99597 77623<br>Email: dpraveen@georgeinstitute.org.in	**Bangladesh**<br>**Dr. Aliya Naheed**<br>Health Systems and Population Studies Division, International Centre for Diarrhoeal Disease Research, Bangladesh, Dhaka.<br>Cell# + (880) 1819 215 611<br>Office # +(880) 28860523-32, (880) 28812353-57 [PABX] Extn. 2521<br>Email: anaheed@icddrb.org	**Sri Lanka**<br>**Prof. Asita de Silva**<br>Clinical Trials Unit Faculty of Medicine, University of Kelaniya, Thalagolla Road, Ragama, Sri Lanka<br>Ph: (94) 112665266, (94) 112665300<br>Email: asitades@gmail.com

(4) What will the study involve for me?

There are two stages of involvement in this study. At this stage, women with GDM will be identified at 24-34 weeks pregnancy through the performance of a standard Oral Glucose Tolerance Test (OGTT). This test will be performed if this has not already been done as part of your routine care during pregnancy. If you are found to have GDM, you will receive

appropriate care for this condition, but we will also provide education to improve your knowledge about diet and physical activity both during and after pregnancy. We will collect your contact details and preferred method of contact. Within the first 6 months after delivery, we may contact you one or more times to check your progress and to remind you to attend an appointment at 6 months post-delivery. When you attend this appointment, we will perform another OGTT. If this test shows that you have developed type 2 diabetes, we will refer you to a doctor for proper care; otherwise, we will invite you to participate in the second stage of the study which is aimed at testing a new lifestyle modification program. This will be discussed with you in more detail if you agree to participate in stage 2.

Pre-Delivery visit

When you visit the hospital for your routine care at 24-34 weeks of pregnancy, you will be offered an OGTT to determine if you have GDM. You may have already had this test done, and, if so, it will not be repeated. The OGTT requires you to fast overnight (no food from 10:00 pm until the time of the test the next morning). When you come to the hospital for your OGTT, you will have a blood test (two ml or half teaspoonful of blood will be obtained), be asked to drink a solution of glucose (sugar), and one and two hours later, you will have blood samples withdrawn (two ml or half teaspoonful of blood will be obtained at each time). If you are diagnosed with GDM during your antenatal visit, you will be briefed about the study. If you are interested in the study, you will need to indicate your voluntary willingness to participate by signing the Consent Form. Whether or not you agree to participate in the study, you will receive standard treatment for GDM, and we will also provide you with information to improve your knowledge about nutrition, physical activity and managing stress both during and after pregnancy. Details of the post pregnancy phase of the study will also be discussed with you. Your permission will be obtained to contact you after delivery, and preferred contact method and details will be collected at this point. The study doctor/research staff will then collect basic information about you. You will be invited to come back when your baby becomes 6 months old (Post-Delivery Visit).

Post-Delivery Visit

A blood test and another OGTT will be performed. At this stage if you are diagnosed with type 2 diabetes, you will be referred to a doctor for proper care. If not, you will be asked to participate in the post-pregnancy stage 2 of the study, which will test the lifestyle modification program. This will be explained to you in detail and you can make a decision then about whether or not you wish to participate in this stage of the study.

(5) How much of my time will the study take?

Interviews conducted by the research team will take between 40 to 60 minutes. We appreciate your other commitments and will fit in with your schedule.

(6) Who can participate in this study?

You can participate in this study if:

- You are aged 18 years or older, and are 24-34 weeks pregnant

You will not be able to take part in the study if:

- you have already had a test that shows that you do not have GDM
- your residence is too far from hospital
- you or your family does not have a usable mobile phone

(7) Do I have to be in the study? Can I withdraw from the study once I have started?

At this stage, the actual study (recruitment of participants) has not yet began. We will approach you for a second stage consent between 3-9 months after your delivery. However, being in this study is completely voluntary and you do not have to take part. Your decision whether to

participate will not affect your current or future relationship with the researchers or any individuals or organizations related to this study.

If you decide to take part in the study and then change your mind later, you are free to withdraw at any time by informing to the doctor or study facilitator at the hospital. If you decide to withdraw from the study, we will not collect any more information from you. Please let us know at the time when you withdraw what you would like us to do with the information we have collected about you up to that point. If you wish, your information will be removed from our study records and will not be included in the study results.

(8) What are the possible risks and benefits of participation in this study?

Risks
There will be no costs associated for being in the study. This study will not involve the use of any drugs, medications or procedures. The risks of participating in this study include:
Blood collection: There may be temporary bruising, discomfort, or local irritation of the skin where the blood sample is taken.
Giving up your time to participate in the study: This might cause some inconvenience but we will try to fit the program in to your schedule (e.g., hospital visits).
Benefits
This study will provide you with some information about healthy lifestyle changes to control your blood glucose levels and the risks of developing type 2 diabetes. All blood test results will be provided to you, and you can show them to your usual doctor.

(9) What are my responsibilities if I take part in this study?

If you take part in this study you need to agree to

- provide correct information to the best of your knowledge
- visit the trial centre for the post-delivery visit
- give blood samples as per the schedule advised by the study team
- While you are in this study you should not participate in any other research study.
- Inform the study team if you do not want to continue your participation in the study.

(10) What happens if I suffer injury or complications as a result of the study?

If you suffer any injuries or complications as a direct result of being in the study, you should contact the study doctor who will assist you in arranging the appropriate medical treatment, as soon as possible. The sponsor(s) holds insurance policies to cover medical care and/or compensation in case of any such events.

If you have any questions concerning the availability of medical care or if you think you have experienced a research-related illness, injury or emergency, contact

Dr. ____________________________ Phone: __________________________________
(Site level doctor details will be inserted later)

(11) What if new information becomes available about the study?

You will be informed in a timely manner if any new information becomes available that may be relevant to your willingness to continue participation in the study.

(12) What happens if the research study is terminated?

Your participation in this study may be stopped if the study is terminated. The study doctor will make sure you receive appropriate ongoing care. You do not have to participate in this study to receive medical advice for any condition.

(13) What will happen to information about me that is collected during the study?

All information collected about you will be kept strictly confidential. Your initials and date of birth will be used as identifiers in the study. Only study monitors, auditors, and the Ethics Committee will be granted direct access to your records for verification of study procedures and/or data, without violating your confidentiality, to the extent permitted by the applicable laws. By signing a written informed Consent Form, you authorise such access. Study findings may be published but you will not be individually identified in these publications. All records and data relating to the study will be destroyed in a secure manner, 15 years after the end of this study while protecting your confidentiality.

(14) What will happen to my blood sample after it has been used?

The blood sample/s you provide during the study will not be stored for further use. Copies of the test reports will be given to you for your records. Your results will be kept confidential and will not be disclosed to any third party.

(15) What will happen to my treatment when the study is finished?

At this stage of the study no treatment or intervention is involved. Your doctor will continue to provide you with appropriate care.

(16) Will I be paid for taking part in this study?

You will not be paid for taking part in this study. However, you will be reimbursed for reasonable travel expenses for study visits to the hospital.

(17) Can I tell other people about the study?

Yes, you are welcome to tell other people about the study.

(18) What if I would like further information about the study?

When you have read this information, a member of the research team will be available to discuss it with you further and answer any questions you may have.

(19) Will I be told the results of the study?

You have a right to receive feedback about the overall results of this study. You can tell us that you wish to receive feedback by ticking the relevant box at the end of the consent form. Please also tick your preferred method of receiving information.
The results of the blood tests will be provided to you as soon as they are available from the laboratory.

(20) What if I have a complaint or any concerns about the study?

Research involving humans is reviewed by an independent group of people called a Human Research Ethics Committee (HREC). The ethical aspects of this study have been approved by the HREC of *[to be replaced by relevant institution and protocol number].* As part of this process, we have agreed to carry out the study according to the Australian *National Statement on Ethical Conduct in Human Research (2007)*.

If you are concerned about the way this study is being conducted or you wish to make a complaint to someone independent from the study, please contact the university using the details outlined below. Please quote the study title and protocol number.

The Manager, Ethics Administration, University of Sydney:
Telephone: +61 2 8627 8176
Email: ro.humanethics@sydney.edu.au
Fax: +61 2 8627 8177 (Facsimile)

If you have any complaints regarding the research you may also contact the Patient Representative at your hospital/ the member secretary of the overseeing ethics committee, whose contact details are provided below.

[relevant ethics committee from below]

India – Details of site specific hospital HRECs will be added later.

Local contact details in Bangladesh and Sri Lanka

**Bangladesh** IRB Coordinator, Research Administration<br>Office of the Deputy Executive Director<br>Icddr,b, Mohakhali, Dhaka-1212<br>Mobile: +88 01711428989<br>Direct: +880-2-9827084<br>PABX: 9827001-10, Extn: 3206<br>Fax: +880-2-9827077<br>Email: salamk@icddrb.org<br>Web: www.icddrb.org	**Sri Lanka**<br>Secretary<br>Ethics Review Committee,<br>Faculty of Medicine<br>University of Kelaniya,<br>Thalagolla Road, Ragama, Sri Lanka<br>Email: ercmed@kln.ac.lk<br>Tel: +94112961142

Thank you for considering participation in this study!

LIVING – Stage 1 CONSENT FORM

Study Title: LIVING- Lifestyle InterVention IN Gestational diabetes

Study/Protocol Number: 1093171

Subject Initials: ______________________Subject's Name: ______________________

Date of Birth/Age: ______________________

Please read the below mentioned statements and confirm your acceptance to participate in the study by giving an initial in the right hand side box against each statement.	*Initial box (Subject)*
(i) I confirm that I have read and understood the participant information statement dated dated [add date] for the above study and have had the opportunity to ask questions.	[ ]
(ii) I understand that my participation in the study is voluntary and that I am free to withdraw at any time, without giving any reason, without my medical care or legal rights being affected.	[ ]
(iii) I understand that the Sponsor of the clinical trial, others working on the Sponsor's behalf, the Ethics Committee and the regulatory authorities will not need my permission to look at my health records both in respect of the current study and any further research that may be conducted in relation to it, even if I withdraw from the trial. I agree to this access. However, I understand that my identity will not be revealed in any information released to third parties or published.	[ ]
(iv) I agree not to restrict the use of any data or results that arise from this study provided such a use is only for scientific purpose(s).	[ ]
(v) I agree to take part in the above study.	[ ]

Subject/ legally acceptable representative	______________________ Name	______________________ Signature/Thumb Impression	______________ Date
Study Investigator	______________________ Name	______________________ Signature	______________ Date

Impartial Witness

______________________ ______________________ ______________
__________ Name ______ Signature _____ Date

Would you like to receive feedback about the overall results of this study?

YES ☐ **NO** ☐

If you answered **YES**, please indicate your preferred form of feedback and address:

☐ Postal: ____
____

☐ Email: ____

Participant to complete:

Name: ____

Signature: ____________________________________ Date: ____________________

Interviewer's name: ____

I have explained the nature and purpose of the study to the above participant and have answered their questions.

Signature: ________________________________ Date: ____________

10.3 ANNEXURE 3 – PISCF FOR STAGE 2 CONSENT – MAIN TRIAL V4.0

Lifestyle InterVention IN Gestational Diabetes (LIVING)
A lifestyle intervention program for the prevention of type 2 diabetes mellitus among South Asian women with gestational diabetes mellitus

PISCFs FOR STAGE 2 CONSENT_MAIN TRIAL_V 4.0

Study/Protocol Number-1093171

CHIEF INVESTIGATOR (SUPERVISOR) NAME
Prof. Anushka Patel

The George Institute for Global Health
Level 5, 1 King street Newtown NSW, Australia 2042,PO Box M201,Missenden Road
NSW 2050,Australia
T +61 2 8052 4439 | **F** +61 2 9993 4502
Email: apatel@georgeinstitute.org |
Website: www.georgeinstitute.org

Lifestyle **I**nter**V**ention **IN G**estational Diabetes (LIVING)
STAGE 2 PARTICIPANT INFORMATION STATEMENT AND CONSENT FORM

(1) Why do I have to read this document?

You are invited to take part in a research study called LIVING (Lifestyle InterVention IN Gestational diabetes). This study will compare a low intensity post-delivery lifestyle program to usual care among women diagnosed with Gestational Diabetes Mellitus (GDM) during their recent pregnancy.

Before you decide to participate, it is important for you to understand why the research is being conducted, and what it will involve. Please take time to read the following information carefully, and if you wish, please discuss it with your doctor, other healthcare provider, relatives and friends. Your participation is voluntary and you are entirely free to decide whether or not to take part in this study. If you choose to take part, you are free to withdraw at any time, without giving any reason. If you choose not to take part, your medical care or legal rights will not be affected and you will continue to receive the usual care given by your doctors and other healthcare providers. If there is anything that is not clear, or if you would like to have more information, please do not hesitate to ask the doctor or the study team.
The trial doctor/research coordinator will discuss the study with you in detail. Once you understand the study and if you have no objections to your involvement, you will be asked to sign the Consent Form (last two pages of this document). By signing the Consent Form you do not alter your legal rights, but you indicate that you understand the information and have no objection to participating in the study. You will be given a copy of this Participant Information Statement and Consent Form to keep for your records.
(2) What is this study about?
Gestational diabetes mellitus (GDM) is a form of diabetes consisting of high blood glucose (sugar) levels during pregnancy. It develops in 4% pregnancies worldwide and is associated with complications to both mother and baby. In South Asia, the GDM rate is much higher and

appears to be increasing over time. High blood glucose levels usually disappears after pregnancy but women who have had GDM and their children are at an increased risk of developing type 2 diabetes later in life. Type 2 diabetes is a lifelong condition usually requiring long term medication use and associated with increased risks of heart disease, stroke, kidney disease and eye disease. Studies from India suggest that 35-40% of women who have had GDM will develop type 2 diabetes within 5 years of delivery.
There is evidence that lifestyle modification programs can be useful in delaying the development of type 2 diabetes among other groups of people at high risk of developing this condition. However, it is not known whether a lifestyle modification program developed for women in South Asia with previous GDM can be successful is preventing the development of type 2 diabetes.
In this study, we will develop and test a new low intensity lifestyle program that has a high probability of being feasible, acceptable and affordable in the South Asian context for women with prior GDM.

(3) Who is running this study?

In India, the study is being conducted by the All India Institute of Medical Sciences, New Delhi and The George Institute for Global Health, Hyderabad. In Bangladesh the study is being conducted by International Centre for Diarrhoeal Disease Research, Bangladesh (icddr,b), Dhaka and in Sri Lanka by the University of Kelaniya, Colombo. The study is funded by the Indian Council of Medical Research and the National Health and Medical Research Council of Australia and as a part of The Global Alliance for Chronic Diseases scheme.

The study is being carried out by following researchers:

**Professor Nikhil Tandon**<br>(PI and co-Chair)<br>All India Institute of Medical Sciences<br>New Delhi, India	**Professor Anushka Patel**<br>(PI and co-Chair)<br>The George Institute for Global Health<br>Sydney, Australia

Details of local researchers:

**India-North**	**India-South**	**Bangladesh**	**Sri Lanka**
**Dr. Prabhakaran Dorairaj**<br>Centre for Chronic Disease Control (CCDC),<br>C-1/52, Second Floor, Safdarjung Development Area (SDA), New Delhi - 110016<br>Tel: 011-41618456Email: dprabhakaran@ccdcindia.org	**Dr. Praveen Devarsetty**<br>The George Institute for Global Health, India<br>Unit No. 301, Second Floor, ANR Centre, Road No.1, Banjara Hills | Hyderabad - 500 034 | Telangana India<br>Ph: +91 40 3099 4444, Mobile: +91 99597 77623<br>Email: dpraveen@georgeinstitute.org.in	**Dr. Aliya Naheed**<br>Health Systems and Population Studies Division, icddr,b, Dhaka.<br>Cell# + (880) 1819 215 611<br>Office # +(880) 28860523-32, (880) 28812353-57 [PABX] Extn. 2521<br>Email: anaheed@icddrb.org	**Prof. Asita de Silva**<br>Clinical Trials Unit Faculty of Medicine, University of Kelaniya,<br>Thalagolla Road, Ragama, Sri Lanka<br>Ph: (94) 112665266, (94) 112665300<br>Email: asitades@gmail.com

(4) What will the study involve for me?

You were diagnosed with gestational diabetes mellitus during your recent pregnancy. We will test your blood glucose at a post-delivery visit, 3 to 18 months after childbirth, and invite you to participate in the LIVING study if this test shows that you are not diabetic.

The following will occur during study visits:

Post-Delivery Visit

An Oral Glucose Tolerance Test (OGTT), and HbA1c test will be performed. The OGTT requires you to fast overnight (no food from 10:00 pm until the time of the test the next morning). When you come to the hospital for your OGTT, you will have a blood test (two ml or half teaspoonful of blood will be obtained), be asked to drink a solution of glucose (sugar), and one and two hours later, you will have blood samples withdrawn (two ml or half teaspoonful of blood will be obtained at each time). At this stage if you are diagnosed with type 2 diabetes, you will be referred to a doctor for proper care. If not, you will be invited to participate in the LIVING study, which will test the lifestyle modification program.

Randomisation visit

After you agree to participate in the study, you will be asked questions about your medical history, the medicines you are taking, and lifestyle factors, such as smoking, diet and exercise. You will have your heart rate, blood pressure, height, weight, and waist circumference measured. You will also be asked questions about any recent visits to, tests performed by, or treatments given by any healthcare provider.

You will be randomly (like with a toss of a coin) allocated to either having your normal post-pregnancy care (control arm), or in addition to your normal post-pregnancy care, take part in a lifestyle modification program (intervention arm) aimed at promoting physical activity, nutrition, and reduced stress levels.

If you are allocated to the control arm, you will be seen at the hospital by the study team every 6 months for an average of 2 years (follow-up study visits), for blood tests and other assessments.

If you are allocated to the intervention arm, you will be seen at the hospital by the study team every 6 months for an average of 2 years (study visits), for blood tests and other assessments. In addition, you will participate in a lifestyle modification program that includes 4 face-to-face group sessions, combined with ongoing support through a range of activities that may include text or voice messaging, and phone calls.. The group sessions may be videorecorded, audiorecorded, and/or observed remotely via video/telephone calls by the research team. Any videorecording will be focused on the facilitator only.

6-month visits

At these visits, you will be asked about lifestyle factors, such as diet, exercise, and tobacco use. You will have your heart rate, blood pressure, weight, and waist circumference measured. You will be asked questions about any recent visits to, tests performed by, or treatments given by any healthcare provider. Any serious new health problems will also be recorded.

12-month and end-of-study visits

At these visits, you will be asked about lifestyle factors such as diet, exercise, and tobacco use. You will have your heart rate, blood pressure, weight, and waist circumference measured. You will be asked questions about any recent visits to, tests performed by, or treatments given by any healthcare provider. Any serious new health problems will also be recorded. In addition blood tests will be performed, including an Oral Glucose Tolerance Test (OGTT). This test requires you to fast overnight (no food from 10:00 pm until the time of the test the next day). When you come to the hospital for your OGTT, you will have a blood test (two ml or half teaspoonful of blood will be obtained), be asked to drink a solution of glucose, and two hours later, you will have another blood tests (two ml or half teaspoonful of blood will be obtained again).

Process evaluation

At the end of the study, you may be invited to take part in an interview which aims to explore your views on the benefits, disadvantages, and acceptability of the lifestyle modification program. It can be a semi structured interview or a focus group discussion (FGD).

The interview will be informal with several questions being asked. It will cover information about your life and your health experience and views about the program. The interview will last for approximately one hour and may occur at the same time as your end of study visit, or at a separate time after this, either at the hospital or at your home. During the interview if a question is asked that you do not want to answer, please just say so. The researcher will then move on to another question. The researcher will record the interview, and afterwards the tapes will be used to make a written record. The written record will only be identified by your initials and a study number. Anything you say will be completely confidential and cannot be traced back to you when the research results are published.
We may also conduct focus group discussions where we will ask questions related to your experiences with the intervention. About 6-8 women will participate in each group discussion. All FGDs will be digitally recorded, professionally transcribed and your name and personal details will be removed for analysis purposes. The data will be analysed by a small team of experienced researchers and will be stored on a secure, password protected folder accessible only to the researchers. We seek permission to use quotes from your interview in our reports but none of this information will allow identification of an individual person. If you are included in process evaluation, a detailed participant information sheet will be given to you before obtaining a consent from you to participate in the study.

(5) How much of my time will the study take?

The intervention (only for members of the intervention group) includes 4 face-to-face group sessions (one session per week) over the first 6 months. Approximately 2 hours of your time will be required for each face-to-face group session that includes the intervention, as well as relevant study procedures. Apart from this, you are also encouraged to follow a lifestyle modification program as part of your routine daily schedule as per your convenience.
Follow-up visits for all participants, including blood tests and other investigations, which will take about 2 hours each visit, will be conducted every 6 months at the hospital.

(6) Who can participate in this study?

You can participate in this study if:

- You are aged 18 years or older, and diagnosed with GDM during the antenatal period.

You will not be able to take part in the study if:

- you have already developed type 2 diabetes
- the travel time from your residence to the hospital is over 2 hours (unless individual circumstances will not impede hospital attendance for study visits)
- you or your family do not have a usable mobile phone

(7) Do I have to be in the study? Can I withdraw from the study once I have started?

Being in this study is completely voluntary and you do not have to take part. Your decision whether to participate will not affect your current or future relationship with the researchers or any individuals or organizations related to this study.

If you decide to take part in the study and then change your mind later, you are free to withdraw at any time by informing the doctor or study facilitator at the hospital.

If you decide not to take part in the study, and you wish to continue treatment, you will still receive the standard treatment available for your condition. It is important that you discuss the alternatives to participating in this study with your doctors.

Data that can be withdrawn:

If you decide to withdraw from the study, we will not collect any more information from you. Please let us know at the time when you withdraw what you would like us to do with the information we have collected about you up to that point. If you wish, your information collected during in-depth interviews will be removed from our study records and will not be included in the study results, up to the point that we have analysed and published results.

Data that cannot be withdrawn:

If you decide to withdraw from the study, we will not collect any more information from you. However, we wish to inform you that due to the nature of group discussions it will not be possible to delete the information from video or audio recordings. In this case, any information already collected will be kept in our records and may be included in the study results.

(8) What are the possible risks and benefits of participation in this study?

Risks

There will be no costs associated for being in the study. This study will not involve use of any drugs, medications or procedures. The risks of participating in this study include:
Blood collection: There may be temporary bruising, discomfort, or local irritation of the skin where the blood sample is taken.
Giving up your time to participate in the study: This might cause some inconvenience but we will try to fit the program in to your schedule (e.g., hospital visits).
Interview: At the end of the study, during the interview there is a risk that a topic could be raised that you may find upsetting. If you find this, please say so. The recording will be stopped immediately. The interview and recording will only start again if you agree and a different topic will be discussed. You might worry afterwards about something you said in the interview. There is no need for this as everything is treated in strict confidence. All the recordings and written records will be made anonymous. If you are still worried, you can contact the interviewer to discuss your concerns.

Benefits

This study will provide you with some information about healthy lifestyle changes to control your blood glucose levels and the risks of developing type 2 diabetes, independent of the arm to which you are randomised. All study test results will be provided to you, which you can show to your usual doctor.

While it is hoped that this study improves medical knowledge and may improve treatment for women who have had GDM, it may not be of direct benefit to you beyond your usual treatment that you would receive from your doctor, and other healthcare providers.
Information about the study may be useful in the future to a large number of people if the lifestyle modification program is shown to reduce the risks of developing abnormal blood glucose levels and / or type 2 diabetes, or otherwise.

At the end of the study, you will have a chance to talk about your experiences, which you may find helpful.

(9) What are my responsibilities if I take part in this study?

If you take part in this study you need to agree to

- provide correct information to the best of your knowledge, including information on blood glucose tests during your recent pregnancy
- visit the trial centre as per the study visits schedule advised by the study team
- discuss your current medications with the study team and any new medications prescribed by other doctors
- agree to give blood samples as per the schedule advised by the study team

- read the SMSs, or listen to the voice messages, sent by the study team. If you have lost your phone, inform the study team
- If you become pregnant again, inform the study team.
- tell the study team about any medical problems, doctor visits, hospital visits, or medical procedures that you have while you are in the study
- not participate in any other interventional research study while you are in this study
- inform the study team if you do not want to continue your participation in the study.

(10) What happens if I suffer injury or complications as a result of the study?

If you suffer any injuries or complications as a direct result of being in study, you should contact the study doctor, who will assist you in arranging the appropriate medical treatment, as soon as possible. The sponsor(s) holds insurance policies to cover medical care and/or compensation in case of any such events.

If you have any questions concerning the availability of medical care, or if you think you have experienced a research-related illness, injury or emergency, contact

Dr. ___________________________ Phone: _________________________________
(Site level doctor details will be inserted later)

(11) What if new information becomes available about the study?
You will be informed in a timely manner if any new information becomes available that may be relevant to your willingness to continue participation in the study.

(12) What will happen to my treatment when the research study is completed?
After you complete the study, your doctor will decide, in consultation with you, any treatment that is best for you. In addition, the study team will advise you to inform them and see the doctor promptly if any major health problems occur within 1 month of study completion.
(13) What happens if the research study is terminated?
Your participation in this study may be stopped if the study is terminated. The study doctor will make sure you receive appropriate ongoing care. You do not have to participate in this study to receive medical advice for any condition.

(14) What will happen to information about me that is collected during the study?
All information collected about you will be kept strictly confidential. Your initials and date of birth will be used as identifiers in the study. Only study monitors, auditors, and the Ethics Committee will be granted direct access to your records for verification of study procedures and/or data, without violating your confidentiality, to the extent permitted by the applicable laws. By signing a written Informed Consent Form, you authorise such access. Study findings may be published, but you will not be individually identified in these publications. All records and data relating to the study will be destroyed in a secure manner, 15 years after the end of this study, while protecting your confidentiality.

(15) What will happen to my blood sample after it has been used?
The blood sample/s you provide during the study will not be stored for further use. Copies of the test reports will be given to you for your records. Your results will be kept confidential and will not be disclosed to any third party.

(16) Will I be paid for taking part in this study?
You will not be paid for taking part in this study. However, you will be reimbursed for reasonable travel expenses for study visits to the hospital.
(17) Can I tell other people about the study?

Yes, you are welcome to tell other people about the study.

(18) What if I would like further information about the study?

When you have read this information, a member of the research team will be available to discuss it with you further, and answer any questions you may have.

(19) Will I be told the results of the study?

You have a right to receive feedback about the overall results of this study. You can tell us that you wish to receive feedback by ticking the relevant box at the end of consent form. Please also tick your preferred method of receiving information.

The results of the blood tests will be provided to you as soon as they are available from the laboratory.

(20) What if I have a complaint or any concerns about the study?

Research involving humans is reviewed by an independent group of people called a Human Research Ethics Committee (HREC). The ethical aspects of this study have been approved by the HREC of *[relevant institution and protocol number].* As part of this process, we have agreed to carry out the study according to the *National Statement on Ethical Conduct in Human Research (2007)*.

If you are concerned about the way this study is being conducted or you wish to make a complaint to someone independent from the study, please contact the university using the details outlined below. Please quote the study title and protocol number.

The Manager, Ethics Administration, University of Sydney:
Telephone: +61 2 8627 8176
Email: ro.humanethics@sydney.edu.au
Fax: +61 2 8627 8177 (Facsimile)

If you have any complaints regarding the research you may also contact the Patient Representative at your hospital/ the member secretary of the overseeing ethics committee, whose contact details are provided below.

[relevant ethics committee from below]

India – Details of site specific hospital HRECs will be added later.

Details of local contacts in Bangladesh and Sri Lanka

**Bangladesh**	**Sri Lanka**
IRB Coordinator, Research Administration<br>Office of the Deputy Executive Director<br>Icddr,b, Mohakhali, Dhaka-1212<br>Mobile: +88 01711428989<br>Direct: +880-2-9827084<br>PABX: 9827001-10, Extn: 3206<br>Fax: +880-2-9827077<br>Email: salamk@icddrb.org<br>Web: www.icddrb.org	Secretary<br>Ethics Review Committee,<br>Faculty of Medicine<br>University of Kelaniya,<br>Thalagolla Road, Ragama, Sri Lanka<br>Email: ercmed@kln.ac.lk<br>Tel: +94112961142

Thank you for considering participation in this study!

LIVING – STAGE 2 CONSENT FORM

Study Title: LIVING- Lifestyle InterVention IN Gestational diabetes

Study/Protocol Number: 1093171

Subject Initials: ________________________Subject's Name: ______________________

Date of Birth/Age: ___________________________

	Initial box(Subject)
Please read the below mentioned statements and confirm your acceptance to participate in the study by giving an initial in the right hand side box against each statement.	[ ]
(i) I confirm that I have read and understood the participant information statement dated [add date] for the above study and have had the opportunity to ask questions.	[ ]
(ii) I understand that my participation in the study is voluntary and that I am free to withdraw at any time, without giving any reason, without my medical care or legal rights being affected.	[ ]
(iii) I understand that the Sponsor of the clinical trial, others working on the Sponsor's behalf, the Ethics Committee and the regulatory authorities will not need my permission to look at my health records both in respect of the current study and any further research that may be conducted in relation to it, even if I withdraw from the trial. I agree to this access. However, I understand that my identity will not be revealed in any information released to third parties or published.	[ ]
(iv) I agree not to restrict the use of any data or results that arise from this study provided such a use is only for scientific purpose(s).	[ ]
(v) I agree to take part in the above study.	[ ]

Subject/ legally acceptable representative	________________ Name	________________ Signature/Thumb Impression	________________ Date
Study Investigator	________________ Name	________________ Signature	________________ Date
Impartial Witness	________________ Name	________________ Signature	________________ Date

Would you like to receive feedback about the overall results of this study?
YES ☐ **NO** ☐

If you answered **YES**, please indicate your preferred form of feedback and address:

☐ Postal: ____
____

☐ Email: ____

Participant to complete:

Name: ____

Signature: ______________________________ Date: ____________________

Interviewer's name: ____
I have explained the nature and purpose of the study to the above participant, and have answered their questions.

Signature: ______________________________ Date: ____________________

10.4 ANNEXURE 4 - PISCF FOR FORMATIVE STAGE V1.3

Study/Protocol Number-1093171

CHIEF INVESTIGATOR NAME
Prof. Anushka Patel

The George Institute for Global Health
Level 5, 1 King street Newtown NSW, Australia 2042,PO Box M201,Missenden Road, NSW 2050,Australia
T +61 2 8052 4439 | **F** +61 2 9993 4502
Email: apatel@georgeinstitute.org |
Website: www.georgeinstitute.org

Lifestyle **I**nter**V**ention **IN G**estational Diabetes **(LIVING)**
A lifestyle intervention program for the prevention of type 2 diabetes mellitus among South Asian women with gestational diabetes mellitus

PISCFs FOR FORMATIVE STAGE_V 1.3

Lifestyle **I**nter**V**ention **IN G**estational Diabetes (LIVING)

PARTICIPANT INFORMATION STATEMENT FOR INDEPTH INTERVIEWS - HEALTH PROFESSIONALS

(1) What is this study about?

You are invited to take part in the formative phase of a lifestyle intervention program for the prevention of type 2 diabetes mellitus among South Asian women with gestational diabetes mellitus (GDM).
The main study aims to determine whether a resource- and culturally-appropriate lifestyle modification program in South Asian countries, provided after childbirth, to women with GDM, will reduce the occurrence of abnormal blood glucose levels and type 2 diabetes, in a manner that is affordable and acceptable. In this study, we will develop and evaluate a new low intensity lifestyle program that has a high probability of being feasible, acceptable and affordable in the South Asian context for women with prior GDM.
This formative phase of the study will include gathering of information from the health care providers, hospital administrators and women with current or previous GDM. You have been invited to participate in this study as your hospital is taking part in the study.

This Information Statement tells you about the research study. Knowing what is involved will help you decide if you want to take part in the research. Please take the time to read this information carefully. Please ask questions about anything that is not clear, or you want to know more about.

Participation in this research study is voluntary.

By giving your consent to take part in this study you are telling us that you:

- ✓ Understand what you have read.
- ✓ Agree to take part in the research study as outlined below.
- ✓ Agree to the use of your personal information as described.

You will be given a copy of this Information Sheet to keep.

(2) Who is running the study?

In India, the study is being conducted by the All India Institute of Medical Sciences, New Delhi and The George Institute for Global Health, Hyderabad. In Bangladesh, the study is being conducted by International Centre for Diarrhoeal Disease Research, Bangladesh (icddr,b), Dhaka and by the University of Kelaniya, Sri Lanka. This study is being funded by the National Health and Medical Research Council of Australia and the Indian Council of Medical Research.

The study is being carried out by following researchers:

**Professor Nikhil Tandon**<br>(PI and co-Chair)<br>All India Institute of Medical Sciences (AIIMS)<br>New Delhi, India	**Professor Anushka Patel**<br>(PI and co-Chair)<br>The George Institute for Global Health<br>Sydney, Australia

Details of local researchers:

India-North	India-South	Bangladesh	Sri Lanka
**Dr. Prabhakaran Dorairaj**<br>Centre for Chronic Disease Control, C-1/52, Second Floor, Safdarjung Development Area (SDA), New Delhi - 110016<br>Tel: 011-41618456Email: dprabhakaran@ccdcindia.org	**Dr. Praveen Devarsetty**<br>The George Institute for Global Health, India<br>Unit No. 301, Second Floor, ANR Centre, Road No.1, Banjara Hills | Hyderabad - 500 034 | Telangana India<br>Ph: +91 40 3099 4444, Mobile: +91 99597 77623<br>Email: dpraveen@georgeinstitute.org.in	**Dr. Aliya Naheed**<br>Health Systems and Population Studies Division, International Centre for Diarrhoeal Disease Research, Bangladesh, Dhaka.<br>Cell# + (880) 1819 215 611<br>Office # +(880) 28860523-32, (880) 28812353-57 [PABX] Extn. 2521<br>Email: anaheed@icddrb.org	**Prof. Asita de Silva**<br>Clinical Trials Unit Faculty of Medicine, University of Kelaniya, Thalagolla Road, Ragama, Sri Lanka<br>Ph: (94) 112665266, (94) 112665300<br>Email: asitades@gmail.com

(3) What will the study involve for me?

Interviews will be conducted by the research team and involve gathering information about the diagnosis, management and referral pathways for GDM that could support the LIVING intervention. Interviews will be conducted to explore the feasibility of the intervention program including factors that might influence uptake of the program as part of your routine care with patients. All interviews will be digitally recorded, professionally transcribed and your name and personal details will be removed for analysis purposes. You will be identified by a unique study identification number. The data will be analysed by a small team of experienced researchers and will be stored on a secure, password protected folder accessible only to the researchers.

We seek permission to use quotes from your interview in our reports but none of this information will allow identification of an individual person. At the conclusion of the research,

the information collected will be securely stored for 15 years minimum is accordance with ethics regulations.

(4) How much of my time will the study take?

Interviews conducted by the research team will take between 45 to 90 minutes. We appreciate your work commitments and will fit this in with your schedule.

(5) Who can take part in the study?

Your hospital has agreed to participate in the LIVING study. We are now inviting you to participate in the formative phase of the study.

(6) Do I have to be in the study? Can I withdraw from the study once I have started?

Participating in this evaluation is completely voluntary and you do not have to take part.

You are free to discontinue your involvement with this study at any time by informing us. Your decision whether or not to participate will not affect your current or future relationship with the researchers or organizations related to this study.

If you decide to withdraw from the study, we will not collect any more information from you. Please let us know at the time when you withdraw what you would like us to do with the information we have collected about you up to that point. If you wish, your information will be removed from our study records and will not be included in the study results, up to the point that we have analysed and published the results.

(7) Are there any risks or costs associated with being in the study?

Aside from giving up your time, we do not expect that there will be any risks or costs associated with taking part in this study.

(8) Is this study associated with any financial benefits to researchers and affiliated organisations?

This study is not associated with any financial benefits to researchers and affiliated organizations. This research is intended to better our understanding of lifestyle intervention models in the prevention and delaying of type 2 diabetes for women diagnosed with gestational diabetes. From this process an appropriate standardized lifestyle intervention program will be developed eventually.

(9) What happens if I suffer injury or complications as a result of the study?

We will be only collecting information from you by asking few questions and you will not be exposed to any drug, medication or procedure.

If you suffer any injuries or complications as a direct result of being in the study, you should contact the study investigator as soon as possible, who will assist you in arranging the appropriate medical treatment. In such cases, to cover medical care and/or compensation in case of any such events the sponsor(s) holds insurance policies.

If you have any questions concerning the availability of medical care or if you think you have experienced a research-related illness, injury or emergency, contact

Dr. ____________________________ Phone: __________________________________

(Site level doctor details will be inserted later)

(10) Are there any benefits associated with being in the study?

We intend that this formative phase will assist in the development of the LIVING study intervention which aims to prevent developing type 2 diabetes in women with prior gestational diabetes. We cannot, however, guarantee that it will be of direct benefit to you.

(11) What will happen to information about me that is collected during the study?

By providing your consent, you are agreeing to us collecting personal information about you for the purposes of this research study. Your information will only be used for the purposes outlined in this Participant Information Statement, unless you consent otherwise.

The information collected during the formative phase of the study will be treated as strictly confidential and not released to anyone outside the research team, except as required by law. The information provided to the researchers will not include any personal information and will be identified by a unique study number only. Study findings may be published but you will not be individually identified in these publications.

(12) Can I tell other people about the study?

Yes, you are welcome to tell other people about the study.

(13) What if I would like further information about the study?

When you have read this information, a member of the research team will be available to discuss it with you further and answer any questions you may have.

(14) Will I be told the results of the study?

You have a right to receive feedback about the overall results of this study. You can tell us that you wish to receive feedback by ticking the relevant box on the consent form. You will receive a summary of the research study results after the study is finished.

(15) What if I have a complaint or any concerns about the study?

Research involving humans is reviewed by an independent group of people called a Human Research Ethics Committee (HREC). The ethical aspects of this study have been approved by the HREC of *[to be replaced by relevant institution and protocol number].* As part of this process, we have agreed to carry out the study according to the Australian *National Statement on Ethical Conduct in Human Research (2007)*.

If you are concerned about the way this study is being conducted or you wish to make a complaint to someone independent from the study, please contact the university using the details outlined below. Please quote the study title and protocol number.

The Manager, Ethics Administration, University of Sydney:
Telephone: +61 2 8627 8176
Email: ro.humanethics@sydney.edu.au
Fax: +61 2 8627 8177 (Facsimile)

If you have any complaints regarding the research you may also contact the Patient Representative at your hospital/ the member secretary of the overseeing ethics committee, whose contact details are provided below.

India – Details of site specific hospital HRECs will be added later.

**Bangladesh**	**Sri Lanka**
IRB Coordinator, Research Administration<br>Office of the Deputy Executive Director<br>Icddr,b, Mohakhali, Dhaka-1212<br>Mobile: +88 01711428989<br>Direct: +880-2-9827084<br>PABX: 9827001-10, Extn: 3206<br>Fax: +880-2-9827077<br>Email: salamk@icddrb.org<br>Web: www.icddrb.org	Secretary<br>Ethics Review Committee,<br>Faculty of Medicine<br>University of Kelaniya,<br>Thalagolla Road, Ragama, Sri Lanka<br>Email: ercmed@kln.ac.lk<br>Tel: +94112961142

This information sheet is for you to keep.

Study/Protocol Number-1093171

CHIEF INVESTIGATOR NAME
Prof. Anushka Patel

The George Institute for Global Health
Level 5, 1 King street Newtown NSW, Australia 2042,PO Box M201,Missenden Road
NSW 2050,Australia
T +61 2 8052 4439 | **F** +61 2 9993 4502
Email: apatel@georgeinstitute.org |
Website: www.georgeinstitute.org

Lifestyle **I**nter**V**ention **IN** **G**estational Diabetes (LIVING)

HEALTH PROFESSIONAL CONSENT FORM

I, ... [PRINT NAME], agree to take part in this research study.

In giving my consent I state that:

- ✓ I understand the purpose of the study, what I will be asked to do, and any risks/benefits involved.

- ✓ I have read the Participant Information Statement and have been able to discuss my involvement in the study with the researchers if I wished to do so.
- ✓ The researchers have answered any questions that I had about the study, and I am happy with the answers.
- ✓ I understand that being in this study is completely voluntary, and I do not have to take part. My decision whether to participate will not affect my current or future relationship with the researchers at The George Institute and The University of Sydney
- ✓ I understand that I can withdraw from the study at any time.
- ✓ I understand that personal information about me that is collected over the course of this project will be stored securely and will only be used for purposes that I have agreed to. I understand that information about me will only be told to others with my permission, except as required by law.
- ✓ I understand that the results of this study may be published, and that publications will not contain my name or any identifiable information about me.

Health Professional to complete:

- I agree to take part in an interview for the study. YES ☐ NO ☐
- I agree that the interview be audiotaped. YES ☐ NO ☐
- I agree that some of my words (not my name) may be used in the study report. YES ☐ NO ☐
- I understand that I have the right to withdraw my consent and cease involvement in the study at any time without penalty, either financially or personally. YES ☐ NO ☐

Would you like to receive feedback about the overall results of this study?
YES ☐ **NO** ☐

If you answered **YES**, please indicate your preferred form of feedback and address:

☐ Postal: ____

____

☐ Email: ____

Health Professional to complete:

Name: ____

Signature: ____

Date: ____

Interviewer's name: ____
I have explained the nature and purpose of the study to the above participant and have answered their questions.

Signature: ____________________________________ Date: ________________________

Study/Protocol Number-1093171

CHIEF INVESTIGATOR NAME
Prof. Anushka Patel

The George Institute for Global Health
Level 5, 1 King street Newtown NSW, Australia 2042,PO Box M201,Missenden Road
NSW 2050,Australia
T +61 2 8052 4439 | **F** +61 2 9993 4502
Email: apatel@georgeinstitute.org |
Website: www.georgeinstitute.org

Lifestyle **I**nter**V**ention **IN G**estational Diabetes (LIVING)

PARTICIPANT INFORMATION STATEMENT FOR IN-DEPTH INTERVIEWS-PARTICIPANTS WITH GDM (CURRENT/ PRIOR)

(1) What is this study about?

You are invited to take part in the formative phase of a lifestyle intervention program for the prevention of type 2 diabetes mellitus among South Asian women with gestational diabetes mellitus.

Gestational diabetes mellitus (GDM) is a form of diabetes consisting of high blood glucose (sugar) levels during pregnancy. It develops in 4% pregnancies worldwide and is associated with complications to both mother and baby. In South Asia, the GDM rate is much higher and appears to be increasing over time. High blood glucose levels usually disappear after pregnancy but women with GDM and their children are at an increased risk of developing type 2 diabetes later in life. Type 2 diabetes is a lifelong condition often requiring long term medication use and associated with increased risks of heart disease, stroke, kidney disease and eye disease. Studies from India suggest that 35-40% of women with GDM will develop type 2 diabetes within 5 years of delivery.

There is evidence that lifestyle modification programs can be useful in delaying the development of type 2 diabetes among other groups of people at high risk of developing this condition. However, it is not known whether a lifestyle modification program developed for women in South Asia with previous GDM can be successful in preventing the development of type 2 diabetes.

This study aims to determine whether a resource- and culturally-appropriate lifestyle modification program in South Asian countries, provided to women with GDM after delivery, will reduce the occurrence of abnormal blood glucose levels and type 2 diabetes, in a manner

that is affordable and acceptable. In this study, we will develop and test a new low intensity lifestyle program that is aimed to be acceptable and affordable in the South Asian context for women with prior GDM.
The formative phase of this study will include in-depth interviews with women who either have or were diagnosed with GDM in order to refine the intervention. You have been invited to participate in this study as you were diagnosed with GDM during your current or previous pregnancy.

This Information Statement tells you about the research study. Knowing what is involved will help you decide if you want to take part in the research. Please take the time to read this information carefully. Please ask questions about anything that is not clear, or you want to know more about.

By giving your consent to take part in this study you are telling us that you:

- ✓ Understand what you have read.
- ✓ Agree to take part in the research study as outlined below.
- ✓ Agree to the use of your personal information as described.

You will be given a copy of this Information Statement to keep.

(2) Who is running the study? Who is conducting and sponsoring this study?

In India, the study is being conducted by the All India Institute of Medical Sciences, New Delhi and The George Institute for Global Health, Hyderabad. In Bangladesh, the study is being conducted by International Centre for Diarrhoeal Disease Research, Bangladesh (icddr,b), Dhaka and in Sri Lanka by the University of Kelaniya, Sri Lanka. The study is funded by the Indian Council of Medical Research and the National Health and Medical Research Council of Australia and as a part of The Global Alliance for Chronic Diseases scheme.
The study is being carried out by following researchers:

**Professor Nikhil Tandon**<br>(PI and co-Chair)<br>All India Institute of Medical Sciences (AIIMS)<br>New Delhi, India	**Professor Anushka Patel**<br>(PI and co-Chair)<br>The George Institute for Global Health<br>Sydney, Australia

Details of local researchers:

India-North	India-South	Bangladesh	Sri Lanka
**Dr. Prabhakaran Dorairaj**<br>Centre for Chronic Disease Control, C-1/52, Second Floor, Safdarjung Development Area (SDA), New Delhi - 110016<br>Tel: 011-41618456Email: dprabhakaran@ccdcindia.org	**Dr. Praveen Devarsetty**<br>The George Institute for Global Health, India<br>Unit No. 301, Second Floor, ANR Centre, Road No.1, Banjara Hills | Hyderabad - 500 034 | Telangana India<br>Ph: +91 40 3099 4444, Mobile: +91 99597 77623	**Dr. Aliya Naheed**<br>Health Systems and Population Studies Division, International Centre for Diarrhoeal Disease Research, Bangladesh, Dhaka.<br>Cell# + (880) 1819 215 611	**Prof. Asita de Silva**<br>Clinical Trials Unit Faculty of Medicine, University of Kelaniya, Thalagolla Road, Ragama, Sri Lanka<br>Ph: (94) 112665266, (94) 112665300<br>Email: asitades@gmail.com

	Email: dpraveen@georgeinstitute.org.in	Office # +(880) 28860523-32, (880) 28812353-57 [PABX] Extn. 2521<br>Email: anaheed@icddrb.org	

(3) What will the study involve for me?

Interviews will be conducted by the research team and involve gathering information about the diagnosis, management and referral pathways for GDM that could support the LIVING intervention. Interviews will be conducted to explore the feasibility of the intervention program including factors that might influence uptake of the program as part of routine care provided in the hospitals. All interviews will be digitally recorded, professionally transcribed and your name and personal details will be removed for analysis purposes. You will be identified by a unique study identification number. The data will be analysed by a small team of experienced researchers and will be stored on a secure, password protected folder accessible only to the researchers.

We seek permission to use quotes from your interview in our reports but none of this information will allow identification of an individual person. At the conclusion of the research, the information collected will be securely stored for 15 years minimum is accordance with ethics regulations.

(4) How much of my time will the study take?

The interviews conducted by the research team will take around 45-90 minutes. We appreciate your other commitments and will fit with your schedule.

(5) Who can take part in the study?

You can participate in this study if you have been diagnosed to have GDM in your current or previous pregnancy.

(6) Do I have to be in the study? Can I withdraw from the study once I've started?

Participating in this evaluation is completely voluntary and you do not have to take part.

You are free to discontinue your involvement with this study at any time by informing us. Your decision whether or not to participate will not affect your current or future relationship with the researchers or organizations related to this study.

If you decide to withdraw from the study, we will not collect any more information from you. Please let us know at the time when you withdraw what you would like us to do with the information we have collected about you up to that point. If you wish, your information will be removed from our study records and will not be included in the study results, up to the point that we have analysed and published the results.

(7) Are there any risks or costs associated with being in the study?

There would be no costs associated for being in the study. Aside from giving up your time, we do not expect that there will be any risks or costs associated with taking part in the interviews. If you take part in this evaluation, you will not be paid to participate.

(8) Is this study associated with any financial benefits to researchers and affiliated organisations?

This research is intended to better our understanding of ways to prevent or delay type 2 diabetes among the women diagnosed with gestational diabetes. The formative research is not associated with any financial benefits to the researchers or affiliated organisations.

(9) What happens if I suffer injury or complications as a result of the study?

If you suffer any injuries or complications as a direct result of being in study, you should contact the study doctor as soon as possible, who will assist you in arranging the appropriate medical treatment. In such cases, to cover medical care and/or compensation in case of any such events the sponsor(s) holds insurance policies.

If you have any questions concerning the availability of medical care or if you think you have experienced a research-related illness, injury or emergency, contact

Dr**.** ___________________________ Phone: _________________________________
(Site level doctor details will be inserted later)

(10) Are there any benefits associated with being in this study?

You will not be paid for taking part in this study and there are no other financial benefits (direct or indirect). However, you will be reimbursed for reasonable travel expenses for attending the interviews.

(11) What will happen to information about me that is collected during the study?

By providing your consent, you are agreeing to us collecting personal information about you for the purposes of this research study. Your information will only be used for the purposes outlined in this Participant Information Statement, unless you consent otherwise.

The information collected during the formative phase of the study will be treated as strictly confidential and not released to anyone outside the research team, except as required by law. The information provided to the researchers will not include any personal information and will be identified by a unique study number only. Study findings may be published but you will not be individually identified in these publications.

(12) Can I tell other people about the study?

Yes, you are welcome to tell other people about the study.

(13) What if I would like further information about the study?

When you have read this information, a member of the research team will be available to discuss it with you further and answer any questions you may have.

(14) Will I be told the results of the study?

You have a right to receive feedback about the overall results of this study. You can tell us that you wish to receive feedback by ticking the relevant box on the consent form.

(15) What if I have a complaint or any concerns about the study?

Research involving humans is reviewed by an independent group of people called a Human Research Ethics Committee (HREC). The ethical aspects of this study have been approved by the HREC of *[to be replaced by relevant institution and protocol number].* As part of this process, we have agreed to carry out the study according to the Australian *National Statement on Ethical Conduct in Human Research (2007)*.

If you are concerned about the way this study is being conducted or you wish to make a complaint to someone independent from the study, please contact the university using the details outlined below. Please quote the study title and protocol number.

The Manager, Ethics Administration, University of Sydney:
Telephone: +61 2 8627 8176
Email: ro.humanethics@sydney.edu.au
Fax: +61 2 8627 8177 (Facsimile)

If you have any complaints regarding the research you may also contact the Patient Representative at your hospital/ the member secretary of the overseeing ethics committee, whose contact details are provided below.

India – Details of site specific hospital HRECs will be added later.

Local contact details in Bangladesh and Sri Lanka

**Bangladesh**	**Sri Lanka**
IRB Coordinator, Research Administration<br>Office of the Deputy Executive Director<br>Icddr,b, Mohakhali, Dhaka-1212<br>Mobile: +88 01711428989<br>Direct: +880-2-9827084<br>PABX: 9827001-10, Extn: 3206<br>Fax: +880-2-9827077<br>Email: salamk@icddrb.org<br>Web: www.icddrb.org	Secretary<br>Ethics Review Committee,<br>Faculty of Medicine<br>University of Kelaniya,<br>Thalagolla Road, Ragama, Sri Lanka<br>Email: ercmed@kln.ac.lk<br>Tel: +94112961142

This information sheet is for you to keep.

Study/Protocol Number-1093171

CHIEF INVESTIGATOR NAME
Prof. Anushka Patel

The George Institute for Global Health
Level 5, 1 King street Newtown NSW,
Australia 2042,PO Box M201Missenden
Road
NSW 2050,Australia
T +61 2 8052 4439 | **F** +61 2 9993 4502
Email: apatel@georgeinstitute.org |
Website: www.georgeinstitute.org

Lifestyle **I**nter**V**ention **IN G**estational Diabetes (LIVING)

PARTICIPANTS (WITH CURRENT / PRIOR GDM) CONSENT FORM FOR INDEPTH INTERVIEWS

I, ... [PRINT NAME], agree to take part in this research study.

In giving my consent I state that:

✓ I understand the purpose of the study, what I will be asked to do, and any risks/benefits involved.

✓ I have read the Participant Information Statement, and have been able to discuss my involvement in the study with the researchers if I wished to do so.

✓ The researchers have answered any questions that I had about the study, and I am happy with the answers.

✓ I understand that being in this study is completely voluntary and I do not have to take part. My decision whether to participate will not affect my current or future relationship with my GP or Pharmacist.

✓ I understand that I can withdraw from the study at any time.

✓ I understand that personal information about me that is collected over the course of this project will be stored securely and will only be used for purposes that I have agreed to. I understand that information about me will only be told to others with my permission, except as required by law.

✓ I understand that the results of this study may be published, and that publications will not contain my name or any identifiable information about me.

Participant to complete:

I agree to take part in an interview for the study.	YES ☐	NO ☐
I agree that the interview be audiotaped.	YES ☐	NO ☐
I agree that some of my words (not my name) may be used in the study report.	YES ☐	NO ☐
I understand that I have the right to withdraw my consent and cease involvement in the study at any time without penalty, either financially or personally.	YES ☐	NO ☐

Would you like to receive feedback about the overall results of this study?

YES ☐ **NO** ☐

If you answered **YES**, please indicate your preferred form of feedback and address:

☐ Postal: ____

____

☐ Email: ____

Participant to complete:

Name: ____

Signature: ______________________________________ Date: ______________________

Interviewer's name: ____
I have explained the nature and purpose of the study to the above participant, and have answered their questions.

Signature: ______________________________________ Date: ______________________

Study/Protocol Number-1093171

**CHIEF INVESTIGATOR NAME**<br>Prof. Anushka Patel	The George Institute for Global Health<br>Level 5, 1 King street Newtown NSW, Australia 2042,PO Box M201,Missenden Road<br>NSW 2050,Australia<br>**T** +61 2 8052 4439 | **F** +61 2 9993 4502<br>**Email:** apatel@georgeinstitute.org | **Website:** www.georgeinstitute.org

Lifestyle **I**nter**V**ention **IN G**estational Diabetes (LIVING)
PARTICIPANT INFORMATION STATEMENT FOR FOCUS GROUP DISCUSSIONS-PARTICIPANTS WITH GDM (PRIOR)

(1) What is this study about?

You are invited to take part in the formative phase of a lifestyle intervention program for the prevention of type 2 diabetes mellitus among South Asian women with gestational diabetes mellitus.
Gestational diabetes mellitus (GDM) is a form of diabetes consisting of high blood glucose (sugar) levels during pregnancy. It develops in 4% pregnancies worldwide and is associated with complications to both mother and baby. In South Asia, the GDM rate is much higher and appears to be increasing over time. High blood glucose levels usually disappear after pregnancy but women with GDM and their children are at an increased risk of developing type 2 diabetes later in life. Type 2 diabetes is a lifelong condition often requiring long term medication use and associated with increased risks of heart disease, stroke, kidney disease and eye disease. Studies from India suggest that 35-40% of women with GDM will develop type 2 diabetes within 5 years of delivery.
There is evidence that lifestyle modification programs can be useful in delaying the development of type 2 diabetes among other groups of people at high risk of developing this condition. However, it is not known whether a lifestyle modification program developed for women in South Asia with previous GDM can be successful in preventing the development of type 2 diabetes.
This study aims to determine whether a resource- and culturally-appropriate lifestyle modification program in South Asian countries, provided to women with GDM after delivery, will reduce the occurrence of abnormal blood glucose levels and type 2 diabetes, in a manner that is affordable and acceptable. In this study, we will develop and test a new low intensity lifestyle program that is aimed to be acceptable and affordable in the South Asian context for women with prior GDM.
The formative phase of this study will include group discussion with women who either have or were diagnosed with GDM in order to refine the intervention. You have been invited to participate in this study as you were diagnosed with GDM during your current or previous pregnancy.

This Information Statement tells you about the research study. Knowing what is involved will help you decide if you want to take part in the research. Please take the time to read this information carefully. Please ask questions about anything that is not clear, or you want to know more about.

By giving your consent to take part in this study you are telling us that you:

- ✓ Understand what you have read.
- ✓ Agree to take part in the research study as outlined below.
- ✓ Agree to the use of your personal information as described.

You will be given a copy of this Information Statement to keep.

(2) Who is running the study? Who is conducting and sponsoring this study?

In India, the study is being conducted by the All India Institute of Medical Sciences, New Delhi and The George Institute for Global Health, Hyderabad. In Bangladesh, the study is being conducted by International Centre for Diarrhoeal Disease Research, Bangladesh (icddr,b), Dhaka and in Sri Lanka by the University of Kelaniya, Sri Lanka. The study is funded by the Indian Council of Medical Research and the National Health and Medical Research Council of Australia and as a part of The Global Alliance for Chronic Diseases scheme.

The study is being carried out by following researchers:

**Professor Nikhil Tandon**<br>(PI and co-Chair)<br>All India Institute of Medical Sciences (AIIMS)<br>New Delhi, India	**Professor Anushka Patel**<br>(PI and co-Chair)<br>The George Institute for Global Health<br>Sydney, Australia

Details of local researchers:

India-North	India-South	Bangladesh	Sri Lanka
**Dr. Prabhakaran Dorairaj**<br>Centre for Chronic Disease Control, C-1/52, Second Floor, Safdarjung Development Area (SDA), New Delhi - 110016<br>Tel: 011-41618456<br>Email: dprabhakaran@ccdcindia.org	**Dr. Praveen Devarsetty**<br>The George Institute for Global Health, India<br>Unit No. 301, Second Floor, ANR Centre, Road No.1, Banjara Hills | Hyderabad - 500 034 | Telangana India<br>Ph: +91 40 3099 4444, Mobile: +91 99597 77623<br>Email: dpraveen@georgeinstitute.org.in	**Dr. Aliya Naheed**<br>Health Systems and Population Studies Division, International Centre for Diarrhoeal Disease Research, Bangladesh, Dhaka.<br>Cell# + (880) 1819 215 611<br>Office # +(880) 28860523-32, (880) 28812353-57 [PABX] Extn. 2521<br>Email: anaheed@icddrb.org	**Prof. Asita de Silva**<br>Clinical Trials Unit Faculty of Medicine, University of Kelaniya, Thalagolla Road, Ragama, Sri Lanka<br>Ph: (94) 112665266, (94) 112665300<br>Email: asitades@gmail.com

(3) What will the study involve for me?

Group discussions with selected participants will be conducted to understand the usefulness and acceptability of the intervention program.

If you agree to participate, we will invite you for a group discussion where we will ask questions related to the diagnosis and treatment of gestational diabetes, discuss your experience in accessing services, and seek your opinion about the lifestyle intervention planned for this study. About 6-8 women will participate in each group discussion. All interviews will be digitally recorded, professionally transcribed and your name and personal details will be removed for analysis purposes. You will be identified by a unique study identification number. The data will be analysed by a small team of experienced researchers and will be stored on a secure, password protected folder accessible only to the researchers.

We seek permission to use quotes from your interview in our reports but none of this information will allow identification of an individual person. At the conclusion of the research, the information collected will be securely stored for 15 years minimum is accordance with ethics regulations.

(4) How much of my time will the study take?

The discussions conducted by the research team will take around 45-90 minutes. We appreciate your other commitments and will fit with your schedule.

(5) Who can take part in the study?

You can participate in this study if you have been diagnosed to have GDM in your current or previous pregnancy.

(6) Do I have to be in the study? Can I withdraw from the study once I have started?

Participating in this study is completely voluntary and you do not have to take part.

If you decide to take part in the study and then change your mind later, you are free to withdraw from the study at any time by informing us. Your decision whether or not to participate will not affect your current or future relationship with the researchers at any of the participating institutions and your doctor. However, we wish to inform you that due to the nature of group discussions it will not be possible to delete the information from audio recordings. In this case, any information already collected will be kept in our records and may be included in the study results.

(7) Are there any risks or costs associated with being in the study?

There would be no costs associated for being in the study. Aside from giving up your time, we do not expect that there will be any risks or costs associated with taking part in the health care consultation recording or interviews. If you take part in this evaluation you will not be paid to participate.

(8) Is this study associated with any financial benefits to researchers and affiliated organisations?

This research is intended to better our understanding of ways to prevent or delay type 2 diabetes among the women diagnosed with gestational diabetes. The formative research is not associated with any financial benefits to the researchers or affiliated organisations.

(9) What happens if I suffer injury or complications as a result of the study?

If you suffer any injuries or complications as a direct result of being in study, you should contact the study doctor as soon as possible, who will assist you in arranging the appropriate medical treatment. In such cases, to cover medical care and/or compensation in case of any such events the sponsor(s) holds insurance policies.

If you have any questions concerning the availability of medical care or if you think you have experienced a research-related illness, injury or emergency, contact

Dr. ____________________________ Phone: __________________________________
(Site level doctor details will be inserted later)

(10) Are there any benefits associated with being in this study?

You will not be paid for taking part in this study and there are no other financial benefits (direct or indirect). However, you will be reimbursed for reasonable travel expenses for the group discussion.

(11) What will happen to information about me that is collected during the study?

By providing your consent, you are agreeing to us collecting personal information about you for the purposes of this research study. Your information will only be used for the purposes outlined in this Participant Information Statement, unless you consent otherwise.

The information collected during the formative phase of the study will be treated as strictly confidential and not released to anyone outside the research team, except as required by law. The information provided to the researchers will not include any personal information and will be identified by a unique study number only. Study findings may be published but you will not be individually identified in these publications.

(12) Can I tell other people about the study?

Yes, you are welcome to tell other people about the study.

(13) What if I would like further information about the study?

When you have read this information, a member of the research team will be available to discuss it with you further and answer any questions you may have.

(14) Will I be told the results of the study?

You have a right to receive feedback about the overall results of this study. You can tell us that you wish to receive feedback by ticking the relevant box on the consent form.

(15) What if I have a complaint or any concerns about the study?

Research involving humans is reviewed by an independent group of people called a Human Research Ethics Committee (HREC). The ethical aspects of this study have been approved by the HREC of *[to be replaced by relevant institution and protocol number].* As part of this process, we have agreed to carry out the study according to the Australian *National Statement on Ethical Conduct in Human Research (2007)*.

If you are concerned about the way this study is being conducted or you wish to make a complaint to someone independent from the study, please contact the university using the details outlined below. Please quote the study title and protocol number.

The Manager, Ethics Administration, University of Sydney:
Telephone: +61 2 8627 8176
Email: ro.humanethics@sydney.edu.au
Fax: +61 2 8627 8177 (Facsimile)

If you have any complaints regarding the research you may also contact the Patient Representative at your hospital/ the member secretary of the overseeing ethics committee, whose contact details are provided below.

India – Details of site specific hospital HRECs will be added later.

Local contact details in Bangladesh and Sri Lanka

**Bangladesh**	**Sri Lanka**
IRB Coordinator, Research Administration<br>Office of the Deputy Executive Director<br>Icddr,b, Mohakhali, Dhaka-1212<br>Mobile: +88 01711428989<br>Direct: +880-2-9827084<br>PABX: 9827001-10, Extn: 3206<br>Fax: +880-2-9827077<br>Email: salamk@icddrb.org<br>Web: www.icddrb.org	Secretary<br>Ethics Review Committee,<br>Faculty of Medicine<br>University of Kelaniya,<br>Thalagolla Road, Ragama, Sri Lanka<br>Email: ercmed@kln.ac.lk<br>Tel: +94112961142

This information sheet is for you to keep.

CHIEF INVESTIGATOR NAME
Prof. Anushka Patel

The George Institute for Global Health
Level 5, 1 King street Newtown NSW, Australia 2042,PO Box M201,Missenden Road
NSW 2050,Australia
T +61 2 8052 4439 | **F** +61 2 9993 4502
Email: apatel@georgeinstitute.org |
Website: www.georgeinstitute.org

Lifestyle **I**nter**V**ention **IN G**estational Diabetes (LIVING)

PARTICIPANTS (PRIOR GDM) CONSENT FORM FOR FOCUS GROUP DISCUSSIONS

I, ... [PRINT NAME], agree to take part in this research study.

In giving my consent I state that:

- ✓ I understand the purpose of the study, what I will be asked to do, and any risks/benefits involved.

- ✓ I have read the Participant Information Statement and have been able to discuss my involvement in the study with the researchers if I wished to do so.

- ✓ The researchers have answered any questions that I had about the study and I am happy with the answers.
- ✓ I understand that being in this study is completely voluntary and I do not have to take part. My decision whether to participate will not affect my current or future relationship with my GP or Pharmacist.
- ✓ I understand that I can withdraw from the study at any time.
- ✓ I understand that personal information about me that is collected over the course of this project will be stored securely and will only be used for purposes that I have agreed to. I understand that information about me will only be told to others with my permission, except as required by law.
- ✓ I understand that the results of this study may be published, and that publications will not contain my name or any identifiable information about me.

Participant to complete:

• I agree to take part in an interview for the study.	YES ☐	NO ☐
• I agree that the interview be audiotaped.	YES ☐	NO ☐
• I agree that some of my words (not my name) may be used in the study report.	YES ☐	NO ☐
• I understand that I have the right to withdraw my consent and cease involvement in the study at any time without penalty, either financially or personally.	YES ☐	NO ☐

Would you like to receive feedback about the overall results of this study?
YES ☐ **NO** ☐

If you answered **YES**, please indicate your preferred form of feedback and address:

☐ Postal: ____
____

☐ Email: ____

Participant to complete:

Name: ____

Signature: ______________________________ Date: ____________________

Interviewer's name: ____

I have explained the nature and purpose of the study to the above participant and have answered their questions.

Signature: ______________________________ Date: ______________

10.5 ANNEXURE 5 – PROTOCOL FOR PROCESS EVALUATION V 1.0

Lifestyle InterVention IN Gestational Diabetes (LIVING)
A lifestyle intervention program for the prevention of type 2 diabetes mellitus among South Asian women with gestational diabetes mellitus

PROTOCOL FOR PROCESS EVALUATION_V 1.0

Study/Protocol Number-1093171

CHIEF INVESTIGATOR (SUPERVISOR) NAME
Prof. Anushka Patel

The George Institute for Global Health
Level 5, 1 King street Newtown NSW, Australia 2042,PO Box M201,Missenden Road
NSW 2050,Australia
T +61 2 8052 4439 | **F** +61 2 9993 4502
Email: apatel@georgeinstitute.org |
Website: www.georgeinstitute.org

Lifestyle **I**nter**V**ention **IN** **G**estational Diabetes (LIVING)

PROCESS EVALUATION PROTOCOL

Overview of the LIVING study
The Lifestyle Intervention in Gestational Diabetes (LIVING) study is a randomised controlled trial (RCT) of a low intensity lifestyle modification programme tailored to women with GDM in India, Bangladesh, and Sri Lanka. The multiple interacting components of this complex intervention were informed by several successful studies in the prevention of T2DM and weight gain, which have been described elsewhere. The multicentre study will be undertaken over 60 months (December 2015 to December 2020). 1414 women with prior GDM will be recruited from a total of up to 24 hospitals in these three countries. The primary outcome of the randomised trial is the proportion of women with a worsening of glycaemic category at or prior to the final visit: (i) normal glucose tolerance to impaired fasting glucose (IFG) or impaired glucose tolerance (IGT) or T2DM; and (ii) IFG or IGT to T2DM. The secondary outcomes of the study include change in body weight, waist circumference, blood pressure, fasting blood glucose, physical activity level, and diet.
The intervention comprises four face-to-face group sessions, 84 automated text or pre-recorded voice messages, monthly phone calls, and two ‘intensification’ sessions in the event that weight goals are not achieved within six months of the commencement of the intervention. The LIVING study process evaluation conducted alongside the RCT will inform policy and decision makers about the reasons why and the mechanisms through which the intervention worked, and whether the intervention is likely to be generalisable and scalable in the real world contexts in which they work.
Aims and objectives
The overall aim of this process evaluation is to evaluate and understand what worked and why during the implementation of the LIVING intervention. In addition, this study aims to provide additional information that will assist in the interpretation of the LIVING study results. Specific objectives are as follows:

i. Evaluate for whom the intervention was effective and why

ii. Evaluate whether the intervention was implemented appropriately, and as planned
iii. Evaluate whether the intervention is generalisable, scalable, and sustainable in a real world setting

Methods and analysis

Methodological approaches informing our evaluation framework

The RE-AIM framework will be used to inform the LIVING study's process evaluation framework.

The RE-AIM framework consists of five dimensions of health-related behaviour interventions: *reach* to the target population, *effectiveness* of the intervention, *adoption* by the intended recipients of the intervention, staff, and institutions such as hospitals, *implementation* (including consistency of implementation and costs), and *maintenance* of the intervention effects in specific settings over time. These five dimensions aim to encourage researchers, evaluators and policy makers to identify the elements of health interventions that improve adoption, implementation and sustainability. The RE-AIM framework was considered highly suitable as the LIVING programme was designed with the intention of broader adoption and implementation into policy and practice in the South Asian context and has been used to design our evaluation framework (Figure 1).

The LIVING study process evaluation framework

The evaluation framework (Figure 1) demonstrates each of the intervention components and the relationships among them. Firstly, the framework incorporates the intervention components addressing assumed causal mechanisms. These causal mechanisms (explained in detail in our logic model [Figure 2]) are intended to make it easier to understand why the intervention will work, and to facilitate external scrutiny of their plausibility. These assumptions are underpinned by the key elements of Michie's behaviour change wheel (capability, opportunity, and motivation). Thus, it also serves to test the applicability and robustness of the theory in the context of lifestyle change in women with prior GDM in India, Bangladesh, and Sri Lanka. Secondly, the framework incorporates implementation factors (e.g. reach and recruitment, fidelity and dose, as well as adoption and implementation), mechanisms of impact (e.g. effectiveness, acceptability, and maintenance) and finally, the outcomes of the trial (to be assessed at the end of the study).

Figure 5.1. Evaluation framework for LIVING process evaluation

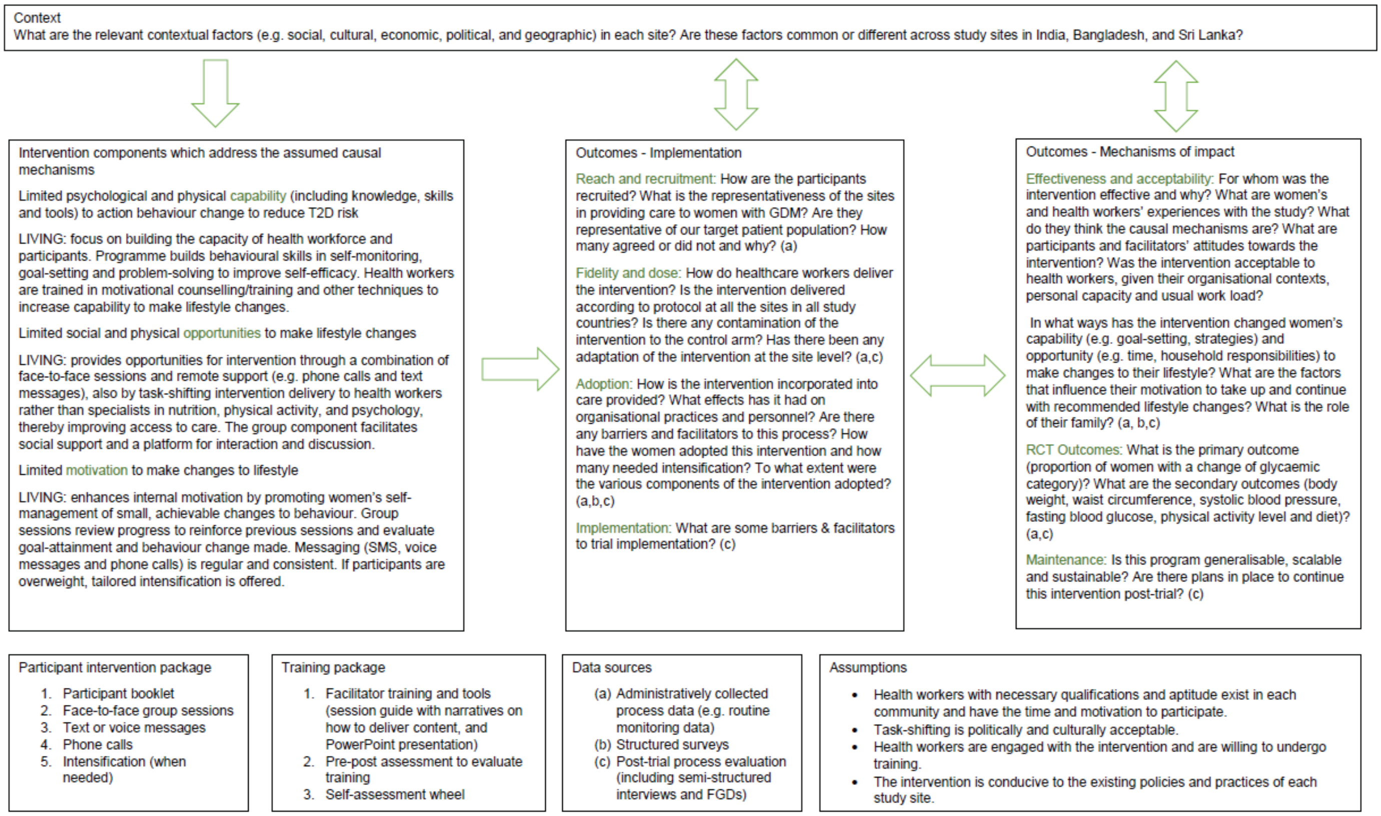

Figure 5.2. Logic model for LIVING to prevent or delay T2DM in women with GDM

LIVING Intervention inputs

- Participant booklet
- Face-to-face group sessions
- Text or voice messages
- Motivational interviewing via telephone
- Intensification
- Training of auxiliary nurses to deliver intervention

Intervention processes and actions

- Participants receive information regarding the health harms of GDM and the potential of lifestyle changes to prevent or delay onset of T2DM
- Participants trained to execute lifestyle change through goal-setting, self-management, and problem-solving through group sessions
- Intervention facilitators reinforce new skills and knowledge through phone calls, text messages and offer intensification after personalised barriers assessment
- Group and individual sessions provide culturally appropriate social support for participants to understand GDM and make lifestyle changes

Changes to capability, motivation and opportunity

- Increased knowledge of health consequences triggers change to diet, exercise, and
- Participants set, monitor and achieve their health-related goals
- Participants devise strategies to improve self-efficacy, and overcome physical, social, and psychological challenges to lifestyle change
- Motivation to maintain changes to lifestyle is sustained through text or voice messages, and phone calls

Changes at organisational level

- Greater integration of departments (obstetrics and gynaecology; paediatrics; and endocrinology and diabetes) and other support staff (nursing, dietetics, and other disciplines) for improved continuity of care
- Improved access to care for women with GDM due to within-system task-shifting

Immediate impacts in women with GDM

- More women attend postpartum screening
- More women with GDM engage in self-care activities to improve and maintain good mental health
- More women with GDM maintain a healthy weight through changes to diet (e.g. increased consumption of fruit, vegetables, and grains; reductions in sugar and salt)
- More women with GDM exercise at least thirty minutes every day

Health outcomes in women with GDM

- Prevention or delay of onset of T2DM
- Improved quality of life and mental health

Methods
A mixed methods design will be used to investigate the components of our process evaluation framework. This will involve the integration of both qualitative and quantitative data in the research process including evaluation datasets, administratively collected process data accessed during monitoring visits, check lists and logs filled at intervention sites, audit reports, quantitative participant evaluation surveys, semi-structured interviews, and focus group discussions.
1. Evaluation datasets: Data from participants will be collected at randomisation, and 6 monthly intervals thereafter, with Oral Glucose Tolerance Tests (OGTT) and HbA1c tests, alternately conducted at the follow-up 6 monthly visits, and both tests at the End of Study visit.
2. Administrative data: Monitoring (at least one every six months) will be conducted by central study staff to ensure compliance with the protocol, and any local requirements. During each visit, the study monitors will fill in a monitoring report on record maintenance, conduct of sessions, and implementation of the programme, and offer feedback to ensure consistency of the implementation. The voice/text messaging report will indicate the number of successful messages sent every month as well as whether the voice message was heard completely, or disconnected early.
3. Programme checklists and logbooks: Programme specific checklists were developed in conjunction with the research team records to assess implementation fidelity, recruitment, intervention delivery, and programme context. Intervention session logs documenting attendance, collection of feedback, and participation of group members will also be analysed.
4. Programme auditing: The delivery of the group sessions will be audited by direct observation, remote observation, or audio or video recording. These data will be analysed alongside the intervention session logs and checklists. At least one of the four sessions conducted for each group will be observed by monitors. The intervention facilitators' conduct of at least one instance of each of the four different sessions will be observed by monitors.
5. Quantitative participant evaluation survey: A barriers and facilitators evaluation survey, administered after the 4th (and final) group session, at approximately 6 months after randomisation, will be undertaken to explore what women have found to be enabling and challenging in executing lifestyle modification following the group sessions. Surveys will list potential barriers to, and facilitators of, physical activity and healthy eating. Participants will also be able to list barriers and enablers that are not listed in the evaluation form.
6. Semi structured interviews: Semi-structured interviews will be conducted with a sub-group of participants, staff and stakeholders at the end of study. Interviews will also be conducted using a maximum variation sampling technique to ensure that diverse opinions are gained from healthcare workers, hospital administrators, and representatives of relevant government agencies with broad representation across the three countries.
7. Group session evaluation form: All women who attend group sessions will fill a feedback form at the end of each session. They will score their overall experience of the intervention session including feedback on intervention content, its presentation as well as facilitator's skills and behaviour.
Below, we describe how each of the data collection tools listed within the evaluation framework will be used.

1. Evaluating whether and how the intervention had impact, for whom and why

Surveys

The barriers and facilitators evaluation survey and group session feedback form will help us understand what aspects of women's lifestyle are preventing them from achieving changes, and which aspects of the intervention they found useful, e.g., self-motivation, weather, scheduling, social support, or information.
The group session feedback form will explore whether the topics covered in the sessions were relevant to them, whether they felt the trainer was well prepared, whether the time allotted for the session was sufficient, and whether the content was organised and easy to follow.

Evaluation datasets

These data will be used to quantitatively evaluate effectiveness (e.g. percent change in outcome variables at the end of the trial). Data from case record forms used in initial and follow-up assessments will be used to compare characteristics of intensification and non-intensification participants to identify any trends in whom the intervention was most effective or least effective for.

Semi-structured interviews and focus group discussions

Semi-structured interviews with health workers, and implementers and monitors of the intervention (see annexure 5.1 for interview schedule), and semi-structured interviews or focus group discussions with participants (see annexure 5.2 for discussion schedule) will seek to gain a deeper understanding of the themes underpinning the surveys described above. In addition, they will seek to address the surveys' gaps (e.g. themes that participants raise in the surveys that were not listed by intervention developers in the pre-formulated survey tool). These data will contribute to a deeper understanding of what participants and the health workers delivering the intervention think the causal mechanisms are, as well as their general attitudes towards the intervention. The discussions will include a specific focus on how (from the perspectives of the participant and the health worker) the intervention impacts elements of Michie's Behaviour Change Wheel, that is, women's capability, opportunity, and motivation.

2. Evaluating whether the trial was implemented appropriately and as planned

In order to investigate the fidelity of the trial, i.e. whether the components of the trial were delivered as intended and in appropriate detail, this process evaluation will utilise mixed methods. Routine monitoring data (e.g. estimated number exposed to recruitment, percent screened for eligibility, percent ineligible for participation, actual number eligible, and actual number who participated) and evaluation datasets will be used to assess reach (whether the intended audience came into contact with the intervention, and how). To assess implementation, and whether poor implementation limited the effects of the intervention, we will use trial administration data pertaining to, for example, the actual number of phone calls, text messages received, and sessions attended. The observation checklist, data obtained from programme auditing, as well as other administrative data collection tools mentioned above will assess the management of group dynamics, the transmission and emphasis of key messages, the provision of all intervention materials, and the participation of the group members.

In addition, pre-post assessment of facilitators will be undertaken to evaluate their training, and all facilitators will participate in a self-assessment wheel. We will use these data to understand how facilitators carried out their roles and responsibilities. These data will be supplemented with semi-structured interviews with participants, and facilitators. The interviews will seek to understand, from various perspectives, issues of fidelity and dose.

3. Evaluating whether the results of the study are generalisable, scalable and sustainable

A third objective of this process evaluation will be to ascertain whether the intervention is generalisable and scalable. Our theoretical framework will allow researchers, evaluators, and policymakers to pinpoint how the intervention, with reference to the key components of each theory incorporated into our framework, can be scaled up in India, Bangladesh, and Sri Lanka as well as tailored to other contexts, if the intervention is found to be effective. Focus groups and semi-structured interviews will be used to understand the barriers and enablers to implementation. In addition, they will seek to understand how the complex intervention was embedded in organisational contexts as the result of individual and collective work to implement the programme.

We will investigate the extent to which the integration of the programme attracted continuous investment by people by working together, as well as how it might be expected that this will continue in the future, and in other contexts. Finally, using semi-structured interviews with key stakeholders in decision-making and management, we will enquire about the extent to which current guidelines, environmental social planning, fiscal measures, and legislation, regulation and service provision are conducive to the scale-up, and sustainability of the intervention at each site.

Sampling

Participants for the interviews will be recruited using maximum variation purposive sampling. Of the 1414 women diagnosed with GDM recruited for the study, approximately half will receive the intervention. 60-90 women with GDM will be recruited at each of the sites in the North India, South India, Bangladesh, and Sri Lanka regions. Evaluation data, session feedback, and data from the barriers and facilitators survey will be obtained from each of the participants in the intervention. For the semi-structured interviews, we will recruit 10-15 women from each of the four regions, arriving at a total of 40-60 interview participants. We will also recruit one facilitator, site investigator, project and hospital management staff, and policy maker from each of the four regions, totalling 20 interview participants. In total, we will recruit 60-80 interview participants. We anticipate that this

sample size will be sufficient to achieve thematic saturation. In the event that thematic saturation has not been reached, we will increase our sample of interview participants. Participants will be recruited by the local site staff, and where relevant, reasons for not participating will be elicited. Written consent will be obtained for the discussions (see annexure 5.3 for Participant Information Sheet and Consent Form for intervention facilitators, site investigators, monitors, implementers, and other stakeholders, and annexure 5.4 for Participant Information Sheet and Consent Form for intervention participants).

Analysis plan

We will undertake thematic analysis of all qualitative data. Data analysis will be conducted contemporaneously with data collection until thematic saturation has been achieved. The development of the coding framework will be informed by the RE-AIM framework as well as the two theories (Michie's Behaviour Change Wheel, and Normalization Process Theory) that have informed our interview and discussion schedules. The analysts will establish themes, under the headings of the evaluation framework: context, causal mechanisms, implementation, mechanisms of impact, and outcomes. Given that the process evaluation is testing a hypothesised causal pathway (described in the logic model – Figure 2), the analysis will aim to scrutinise the extent to which our logic model predicted the actual impact of the intervention, and also identify unexpected mechanisms.

To triangulate data, the analysis team will engage in constant iterative comparison among data from various stakeholders (e.g. auxiliary nurse midwives, participants, and management staff). Our analysis will identify components of the intervention, implementation, and outcomes associated with a large degree of agreement, as well as identify any differences in experiences and perspectives among stakeholders. Data from other sources listed in the framework (including administratively collected process data, and survey data) will be used to triangulate themes. Quantitative data will help inform fidelity and dose as described in the previous section. The framework will aid in triangulating qualitative and quantitative findings, as well as identifying the relationships among context, implementation, mechanisms of impact, and outcomes. Data analyses will be ongoing, and process data will be analysed independently of the outcome evaluation data before the two sets of data are combined. Data will also be triangulated, to increase the reliability of results, using the multiple perspectives of analysts (based in India, Bangladesh, Sri Lanka, and Australia) and the diverse disciplinary (social sciences, medical, legal), and cultural backgrounds of the analysts. In addition, at least one analyst (JS) has not been involved in the development of the intervention and as such, we anticipate that this independent perspective will help to triangulate data, and ensure the reliability of our results.

Ethics and dissemination

All participants for the process evaluation will be given information about the study, and their consent will be obtained. Relevant ethics approval will be obtained beforehand from the respective human research ethics committee of the All India Institute of Medical Sciences, Delhi, India; University of Sydney, New South Wales, Australia; and at each local site in the three countries, India, Bangladesh, and Sri Lanka.

The findings of this process evaluation will be documented in academic publications, and presentations at scientific meetings. In addition, the findings will be presented and discussed during planned stakeholder workshops. The manuscript will be approved by the LIVING study Steering Committee prior to all publications or presentations.

Trial status

The trial was initiated in December 2015 and follow-up will be completed by the end of 2020. The recruitment of the sites, intervention development and its site-specific contextualization as well as the staff training is complete. Results will be reported at the end of 2021.

Annexure 5.1
Interview schedule: (Implementers) facilitators, site investigators, monitors, other stakeholders

Broad descriptive questions	Probes
What are your views on the burden of gestational diabetes in India/Sri Lanka/Bangladesh?	- Do you perceive this issue to be common?<br>- What kinds of support exist (e.g. family, and community, health care settings and national or subnational programs) to help women with gestational diabetes?<br>- What kind of support is available after delivery in women with gestational diabetes?<br>- What is or should be the role of hospitals, primary health care centres in addressing gestational diabetes?
What are your general attitudes towards the intervention?	What are you general attitudes towards the:<br>- Face-to-face group sessions<br>- Voice/text messages<br>- Monthly phone calls<br>- Intensification sessions
Evaluating whether and how the intervention had impact, for whom and why	
Motivation	
In your view, has the LIVING intervention motivated participants to make changes to their lifestyle?	- How have participants responded to the voice/text messages?<br>- How have participants responded to phone calls?<br>- How have participants responded to group sessions?<br>o Have they been well received? Was any aspect of the intervention better received than others? Or not well-received?<br>o Are there aspects of the intervention that participants liked or did not like?<br>- What are the factors that motivated (or did not motivate) participants to participate in the intervention?<br>- What impact has the intervention had on participants' motivation to:<br>o Maintain a healthy weight<br>o Increase consumption of fruit and vegetables<br>o Reduce sugar and salt<br>o Engage in at least thirty minutes of exercise daily

How has your training and practice in delivering the LIVING intervention motivated you?	- What are the factors you find most motivate you in your work?<br>- What are the factors that are least motivating?
Opportunity	
What are your general views about the opportunities that participants have been afforded through the program to make lifestyle changes?	- Has it been useful (or not) for participants to have the opportunity to talk about their health and learn new strategies to make changes to their lifestyle?<br>- Are these opportunities that participants would have had without being a part of the program?<br>- What were the things that made participants want to (or not) to participate?<br>o Physical location of face-to-face sessions<br>o Mode of delivery (e.g. group vs individual components of study)<br>o Social interaction<br>o Health workers/staff<br>o Situation with family<br>o Situation with friends<br>- Could you describe the environment during face-to-face sessions?<br>o Privacy and confidentiality<br>o Participants' level of comfort in sharing thoughts and asking questions<br>o Did you receive any feedback from your participants about their experience? If so, please describe.
What opportunities have you had to receive training and equip yourself with the knowledge and skills to deliver the LIVING intervention?	
Capability	
What has been your experience of working with participants to set, manage and achieve their goals?	
Has the LIVING program been able to build participants' capacity in goal setting and turn difficult habits into 'self-care' activities?	- Have participants successfully applied the skills and strategies they've learned in their own lives? If so, how?<br>- If not, what are the main reasons?<br>o Lack of time<br>o Low self-esteem<br>o Cost<br>o Lack of support
Tell me about your training as part of the LIVING study.	- Did your training and the materials you received give you the

	necessarily skills to deliver the intervention?<br>- Were there times when you felt ill-equipped to deal with a participant, situation or problem?<br>o If so, how could the training be improved?
**Evaluating whether the trial was implemented appropriately and as planned**	
What has it been like for you to be involved in the LIVING study?	- How has your involvement impacted your day-to-day work, and your institution (if relevant)? Were there any benefits or disadvantages for you or your institution?<br>- Did you experience any problems with the general administration of the trial? If so, please explain.<br>- In your view, has the intervention been delivered according the protocol?<br>- In your view, have facilitator guidelines been followed by you and your colleagues?<br>- What has been your experience of recruitment? What are your thoughts about the eligibility criteria?<br>- How did you find follow-up with participants?<br>- Have you experienced any problems in coordinating with other health workers involved in the study?<br>- Do you have any thoughts about the primary outcomes of the study?<br>- Has there been any contamination from the intervention to the control arm?
**Evaluating whether the results of the study are generalisable, scalable and sustainable?**	
What has been your experience in embedding the LIVING program within your organisational context?	- Did this require any changes to organisational practice or structure?<br>- How have you and your colleagues worked individually and collectively to implement the program within your practice?<br>o Were these efforts continuous and/or consistent?<br>o In your view will you be able to continue these efforts into the future?

	o Who were the key people involved in driving the intervention forward? In your view, did they have the appropriate skills?<br>o How supportive of the intervention were others in the organisation?<br>- How do you feel about the intervention becoming a part of your normal work?<br>o Is it likely to become a part of your normal work, if the intervention is found to be successful?
In your view, what are the barriers and enablers to implementation of the LIVING intervention outside of the trial in your context and in other South-Asian contexts?	
Concluding questions	- Are there things we could do to improve the LIVING program or the way it is run?<br>- Is there anything else you would like to share that we have not talked about in this interview?

Annexure 5.2
Interview / focus group discussion schedule: Participant

**Broad descriptive questions**	**Probes**
Evaluating whether and how the intervention had impact, for whom and why	
What are your views on the burden of gestational diabetes in India/Sri Lanka/Bangladesh?	- Do you perceive this issue to be common?<br>- What kinds of support exist (e.g. family, and community, health care settings and national or subnational programs) to help women with gestational diabetes (GDM)?<br>- What kind of support exists after delivery?<br>- What is or should be the role of hospitals, primary health care centres in addressing GDM?
What are your general attitudes towards the intervention?	What are you general attitudes towards the:<br>- Face-to-face group sessions<br>- Phone messages (voice/text)<br>- Monthly phone calls<br>- Intensification sessions
Motivation	
What are your general views on the role of motivation in taking care of yourself?	- What does ‘motivation’ mean to you in practice/in your life?<br>- Is motivation an important factor in making healthy lifestyle choices?<br>- Before taking part in LIVING, when did you find it easy to make healthy lifestyle choices?<br>o When did you find it difficult?<br>- Can you describe a time or situation where you didn’t want to work towards a healthy weight, improve your diet or do exercise?<br>o What would you do when you felt this way?
What has been your experience of the LIVING intervention in motivating you to take care of yourself?	- Have you found the voice/text messages, phone calls, group sessions, and intensification, if any, particularly useful in maintaining or changing your levels of motivation?<br>- If you have had both recorded voice messages and text messages, which did you find more useful?<br>- What impact has it had on your **motivation** to:<br>o Maintain a healthy weight<br>o Increase consumption of fruit and vegetables<br>o Reduce sugar and salt

	o Engage in at least thirty minutes of exercise<br>- Are there factors in your life that affect your motivation that the program has not addressed? If so, please explain.
Opportunity	
What are your general views about the opportunity to be involved in the study (attending face-to-face sessions, being called once a month, and receiving voice/text messages)?	- Has it been useful (or not) to have the opportunity to be involved in the LIVING program?<br>- Are these opportunities you would have had without being a part of the program?<br>- What were the things that have (or have not) made you want to take up the opportunity to participate?<br>o Physical location of face-to-face sessions<br>o Social interaction<br>o Health workers/staff<br>o Features of your phone, or access to a phone<br>- When you attended face-to-face sessions, could you describe the environment?<br>o How did this environment make you feel about yourself and your health?<br>o Did you feel that this was a safe and confidential environment to discuss your experiences of GDM?<br>o Did you feel comfortable asking questions and sharing your thoughts with the group? Why/why not?<br>o Did you feel the session was organised and delivered well?
Outside of the LIVING program, what opportunities have you had (e.g. time, physical space and support) to make changes to your lifestyle?	
Capability	
What has been your experience of skill-building within the LIVING program?	- How do you think the LIVING program is trying to familiarise you with skills and strategies for looking after your health?<br>- Did you have these skills and strategies already?<br>- Have you found these activities useful and/or relevant to your life?
Has the LIVING program been able to build your capacity in goal setting and turn difficult habits into 'self-care' activities?	- What does it mean to 'successfully' apply the skills/techniques from the LIVING program to your life?<br>o What are the changes to your capability you would like to see?<br>- Have you applied the lessons you've learned throughout the program in your daily life? If so, how?

	- If not, what are the main reasons?<br>o Lack of time<br>o Low self-esteem<br>o Cost<br>o Lack of support<br>- Describe any situation where you were, or were not able to, or had difficulty in applying the skills you learned in the LIVING program.<br>- When you felt you were not able to, or experienced difficulty in making changes to your lifestyle, what strategies did you use (if any) to help you work through this?
Has the program changed your perspective on your capacity to make changes to your lifestyle?	- In your view, to what extent is your capability in making changes to your lifestyle within your control?<br>- Have you always felt this way? Has the program changed your feelings about this?<br>- How much of the advice are you still following?<br>- Have there been any benefits to your family (e.g. increases to knowledge and changes to practice?)
Concluding questions	- Are there things we could do to improve the LIVING program or the way it is run?<br>- Would you recommend these sessions to other people if ever conducted in future?<br>- Is there anything else you would like to share that we have not talked about in this discussion?

Annexure 5.3

Lifestyle InterVention IN Gestational Diabetes (LIVING)

A lifestyle intervention program for the prevention of type 2 diabetes mellitus among South Asian women with gestational diabetes mellitus

PISCF FOR PROCESS EVALUATION_V 1.0 (IMPLEMENTER)
Health Worker / Investigator / Study coordinator / Monitor

Study/Protocol Number-1093171

**CHIEF INVESTIGATOR (SUPERVISOR) NAME**<br>Prof. Anushka Patel	The George Institute for Global Health<br>Level 5, 1 King street Newtown NSW, Australia 2042,PO Box M201,Missenden Road<br>NSW 2050,Australia<br>**T** +61 2 8052 4439 | **F** +61 2 9993 4502<br>**Email:** apatel@georgeinstitute.org | **Website:** www.georgeinstitute.org

Lifestyle **I**nter**V**ention **IN G**estational Diabetes (LIVING)

PARTICIPANT INFORMATION STATEMENT AND CONSENT FORM
PROCESS EVALUATION

(1) What is this study about?

The research study LIVING (Lifestyle InterVention IN Gestational diabetes) compares a low intensity post-childbirth lifestyle program to usual care among women diagnosed with Gestational Diabetes Mellitus (GDM) during their recent pregnancy. You were involved in the implementation of the intervention in the LIVING study. You are invited to take part in an interview that aims to explore your views on the benefits, disadvantages, and acceptability of this lifestyle modification program. From this interview, we will gather your perspectives on the intervention, which may be incorporated into the design and delivery of the intervention to optimise it for future implementation. This information sheet gives details of the interview planned. You will be given a copy of this information sheet to keep.

If there is anything that is not clear, or if you would like to have more information, please do not hesitate to ask the study team.

Once you understand the process evaluation, and if you have no objections to your involvement, you will be asked to sign the Consent Form. By signing the Consent Form you do not alter your legal rights, but you indicate that you understand the information and have no objection to participating in the process evaluation.

(2) Who is running the study?

In India, the study is being conducted by the All India Institute of Medical Sciences, New Delhi and The George Institute for Global Health, Hyderabad. In Bangladesh, the study is being conducted by International Centre for Diarrhoeal Disease Research, Bangladesh (icddr,b), Dhaka and by the University of Kelaniya, Sri Lanka. This study is being funded by the National Health and Medical Research Council of Australia and the Indian Council of Medical Research.

The study is being carried out by following researchers:

**Professor Nikhil Tandon**<br>(PI and co-Chair)<br>All India Institute of Medical Sciences (AIIMS)<br>New Delhi, India	**Professor Anushka Patel**<br>(PI and co-Chair)<br>The George Institute for Global Health<br>Sydney, Australia

Details of local researchers:

India-North	India-South	Bangladesh	Sri Lanka
**Dr. Prabhakaran Dorairaj**<br>Centre for Chronic Disease Control, Tower 4, Commercial Complex, C 9, Vasant Kunj, New Delhi-110070<br>Tel: 91 11 43421900<br>Fax: 91 11 43421975 Email: dprabhakaran@ccdcindia.org	**Dr. Praveen Devarsetty**<br>The George Institute for Global Health, India<br>Unit No. 301, Second Floor, ANR Centre, Road No.1, Banjara Hills | Hyderabad - 500 034 | Telangana India<br>Ph: +91 40 3099 4444, Mobile: +91 99597 77623<br>Email: dpraveen@georgeinstitute.org.in	**Dr. Aliya Naheed**<br>Health Systems and Population Studies Division, International Centre for Diarrhoeal Disease Research, Bangladesh, Dhaka.<br>Cell# + (880) 1819 215 611<br>Office # +(880) 28860523-32, (880) 28812353-57 [PABX] Extn. 2521<br>Email: anaheed@icddrb.org	**Prof. Asita de Silva**<br>Clinical Trials Unit Faculty of Medicine, University of Kelaniya, Thalagolla Road, Ragama, Sri Lanka<br>Ph: (94) 112665266, (94) 112665300<br>Email: asitades@gmail.com

(3) What will the study involve for me?

The interview will cover information about your views about the program. It will be conducted at a place of your convenience, either at the hospital or at your home. During an interview, if a question is asked that you do not want to answer, please say so. The researchers will then move on to another question.
All interviews will be audiorecorded, and transcribed, and your name and personal details will be removed for analysis purposes. The data will be analysed by a small team of experienced researchers and will be stored on a secure, password-protected folder accessible only to the researchers. We seek permission to use quotes from your interview in our reports but none of this information will allow identification of an individual person.

(4) How much of my time will the study take?

An interview will take between 45 and 90 minutes. We appreciate your other commitments, and will fit this in with your schedule.

(5) Who can take part in the study?

Individuals involved in the implementation of the LIVING study intervention can take part in this process evaluation.

(6) Do I have to be in the study? Can I withdraw from the study once I've started?

Your participation is voluntary and you are entirely free to decide whether or not to take part in this process evaluation. If you choose to take part, you are free to withdraw at any time, without giving

any reason. If you choose not to take part, your legal rights will not be affected. Your decision whether to participate will not affect your current or future relationship with the researchers or any individuals or organizations related to this study.

Data that can be withdrawn:

If you wish, your information collected during the interview will be removed from our study records and will not be included in the study results, up to the point that we have analysed and published results.

(7) Are there any risks or costs associated with being in the study?

Risks

There will be no costs associated with participating in the process evaluation. This process will not involve use of any drugs, medications or procedures. The risks of participating include:

(i) Giving up your time to participate in the study: This might cause some inconvenience but we will try to fit the discussions in to your schedule.

(ii) Psychological discomfort during the discussion: There is a risk that a topic could be raised that you may find upsetting. If this happens, please say so. The recording of the interview will be stopped immediately. The interview and recording will only start again if you agree, and a different topic will be discussed. You might worry afterwards about something that you said in the discussion. There is no need for this as everything is treated in strict confidence. All the recordings and written records will be made anonymous. If you are still worried, you can contact the researchers to discuss your concerns.

(8) Is this study associated with any financial benefits to researchers and affiliated organisations?

This study is not associated with any financial benefits to researchers and affiliated organisations.

(9) What happens if I suffer injury or complications as a result of the study?

We will be only collecting information from you by asking few questions and you will not be exposed to any drug, medication or procedure.

If you suffer any injuries or complications as a direct result of being in the study, you should contact the study investigator as soon as possible, who will assist you in arranging the appropriate medical treatment. In such cases, to cover medical care and/or compensation in case of any such events the sponsor(s) holds insurance policies.

If you have any questions concerning the availability of medical care or if you think you have experienced a research-related illness, injury or emergency, contact

Dr.__________________________ Phone: _________________________________

(Site level doctor details will be inserted later)

(10) Are there any benefits associated with being in the study?

It is hoped that this process evaluation will contribute to the revision and refinement of the Type 2 diabetes mellitus prevention intervention for women who have had gestational diabetes mellitus. We cannot, however, guarantee that it will be of direct benefit to you.

You will not be paid for taking part in this study. You will be provided refreshments at the discussion.

(11) What will happen to information about me that is collected during the study?

All information collected about you will be kept strictly confidential. Your initials and date of birth will be used as identifiers in the study. Only study monitors, auditors, and the Ethics Committee will be granted direct access to your records for verification of study procedures and/or data, without violating your confidentiality, to the extent permitted by the applicable laws. By signing a written Informed Consent Form, you authorise such access. Study findings may be published, but you will not be individually identified in these publications. All records and data relating to the study will be destroyed in a secure manner, 15 years after the end of this study, while protecting your confidentiality.

(12) Can I tell other people about the study?

Yes, you are welcome to tell other people about the study.

(13) What if I would like further information about the study?

When you have read this information, a member of the research team will be available to discuss it with you further and answer any questions you may have.

(14) Will I be told the results of the study?

You have a right to receive feedback about the overall results of this study. You can tell us that you wish to receive feedback by ticking the relevant box on the consent form. You will receive a summary of the research study results after the study is finished.

(15) What if I have a complaint or any concerns about the study?

Research involving humans is reviewed by an independent group of people called a Human Research Ethics Committee (HREC). The ethical aspects of this study have been approved by the HREC of *[to be replaced by relevant institution and protocol number].* As part of this process, we have agreed to carry out the study according to the Australian *National Statement on Ethical Conduct in Human Research (2007)*.

If you are concerned about the way this study is being conducted or you wish to make a complaint to someone independent from the study, please contact the university using the details outlined below. Please quote the study title and protocol number.

The Manager, Ethics Administration, University of Sydney:
Telephone: +61 2 8627 8176
Email: ro.humanethics@sydney.edu.au
Fax: +61 2 8627 8177 (Facsimile)

If you have any complaints regarding the research you may also contact the Patient Representative at your hospital/ the member secretary of the overseeing ethics committee, whose contact details are provided below.
[relevant ethics committee from below]

Details of local contacts in Bangladesh and Sri Lanka

**Bangladesh**	**Sri Lanka**
IRB Coordinator, Research Administration<br>Office of the Deputy Executive Director<br>Icddr,b, Mohakhali, Dhaka-1212<br>Mobile: +88 01711428989<br>Direct: +880-2-9827084<br>PABX: 9827001-10, Extn: 3206<br>Fax: +880-2-9827077	Secretary<br>Ethics Review Committee,<br>Faculty of Medicine<br>University of Kelaniya,<br>Thalagolla Road, Ragama, Sri Lanka<br>Email: ercmed@kln.ac.lk<br>Tel: +94112961142

Email: salamk@icddrb.org<br>Web: www.icddrb.org	

Thank you for considering participation in this study!

LIVING – PROCESS EVALUATION OF LIVING STUDY INTERVENTION

Study Title: LIVING- Lifestyle InterVention IN Gestational diabetes

Study/Protocol Number: 1093171

Participant Initials: ________________________
Participant's Name: _______________________

Date of Birth/Age:_______________________<br><br>*Please read the below mentioned statements and confirm your acceptance to participate in the process evaluation by giving an initial in the right hand side box against each statement.*		*Initial box(Subject)*<br><br>[ ]	
(i) I confirm that I have read and understood the participant information statement dated …………….. for the above process evaluation and have had the opportunity to ask questions.		[ ]	
(ii) I understand that my participation is voluntary and that I am free to withdraw at any time, without giving any reason, without my medical care or legal rights being affected.		[ ]	
(iii) I agree not to restrict the use of any data or results that arise from this process evaluation provided such a use is only for scientific purpose(s).		[ ]	
(iv) I agree to take part in the above process evaluation.		[ ]	
Subject/ legally acceptable representative	__________________<br>___________ Name	__________________<br>_<br>Signature/Thumb Impression	________________<br>Date
Study Investigator	__________________<br>__________ Name	__________________<br>_____ Signature	________________<br>Date
Impartial Witness	__________________<br>__________ Name	__________________<br>_____ Signature	________________<br>Date

Would you like to receive feedback about the overall results of this process evaluation?
YES ☐ **NO** ☐

If you answered **YES**, please indicate your preferred form of feedback and address:

☐ Postal: ____
____

☐ Email: ____

Participant to complete:

Name: ____

Signature: ________________________________ Date: ________________

Interviewer's name: ____

I have explained the nature and purpose of the process evaluation to the above participant, and have answered their questions.

Signature: ________________________________ Date: ________________

Annexure 5.4

Lifestyle InterVention IN Gestational Diabetes (LIVING)

A lifestyle intervention program for the prevention of type 2 diabetes mellitus among South Asian women with gestational diabetes mellitus

PISCF FOR PROCESS EVALUATION_V 1.0 (PARTICIPANT)

Study/Protocol Number-1093171

CHIEF INVESTIGATOR (SUPERVISOR) NAME
Prof. Anushka Patel

The George Institute for Global Health
Level 5, 1 King street Newtown NSW, Australia 2042,PO Box M201,Missenden Road
NSW 2050,Australia
T +61 2 8052 4439 | **F** +61 2 9993 4502
Email: apatel@georgeinstitute.org |
Website: www.georgeinstitute.org

Lifestyle **I**nter**V**ention **IN G**estational Diabetes (LIVING)

PARTICIPANT INFORMATION STATEMENT AND CONSENT FORM

PROCESS EVALUATION

(1) What is this study about?

The research study LIVING (Lifestyle InterVention IN Gestational diabetes) compares a low intensity post-childbirth lifestyle program to usual care among women diagnosed with Gestational Diabetes Mellitus (GDM) during their recent pregnancy. You are a participant in the intervention group in the LIVING study. You are invited to take part in a discussion that aims to explore your views on the benefits, disadvantages, and acceptability of the lifestyle modification program that you received. From this discussion, we will gather participants' perspectives on the intervention, which may be incorporated into the design and delivery of the intervention to optimise it for future implementation. This information sheet gives details of the discussions planned. You will be given a copy of this information sheet to keep.

If there is anything that is not clear, or if you would like to have more information, please do not hesitate to ask the doctor or the study team.

Once you understand the process evaluation, and if you have no objections to your involvement, you will be asked to sign the Consent Form. By signing the Consent Form you do not alter your legal rights, but you indicate that you understand the information and have no objection to participating in the process evaluation.

(2) Who is running the study?

In India, the study is being conducted by the All India Institute of Medical Sciences, New Delhi and The George Institute for Global Health, Hyderabad. In Bangladesh, the study is being conducted by International Centre for Diarrhoeal Disease Research, Bangladesh (icddr,b), Dhaka and by the University of Kelaniya, Sri Lanka. This study is being funded by the National Health and Medical Research Council of Australia and the Indian Council of Medical Research.

The study is being carried out by following researchers:

**Professor Nikhil Tandon**<br>(PI and co-Chair)<br>All India Institute of Medical Sciences (AIIMS)<br>New Delhi, India	**Professor Anushka Patel**<br>(PI and co-Chair)<br>The George Institute for Global Health<br>Sydney, Australia

Details of local researchers:

India-North	India-South	Bangladesh	Sri Lanka
**Dr. Prabhakaran Dorairaj**<br>Centre for Chronic Disease Control, Tower 4, Commercial Complex, C 9, Vasant Kunj, New Delhi-110070<br>Tel: 91 11 43421900<br>Fax: 91 11 43421975 Email: dprabhakaran@ccdcindia.org	**Dr. Praveen Devarsetty**<br>The George Institute for Global Health, India<br>Unit No. 301, Second Floor, ANR Centre, Road No.1, Banjara Hills | Hyderabad - 500 034 | Telangana India<br>Ph: +91 40 3099 4444, Mobile: +91 99597 77623<br>Email: dpraveen@georgeinstitute.org.in	**Dr. Aliya Naheed**<br>Health Systems and Population Studies Division, International Centre for Diarrhoeal Disease Research, Bangladesh, Dhaka.<br>Cell# + (880) 1819 215 611<br>Office # +(880) 28860523-32, (880) 28812353-57 [PABX] Extn. 2521<br>Email: anaheed@icddrb.org	**Prof. Asita de Silva**<br>Clinical Trials Unit Faculty of Medicine, University of Kelaniya, Thalagolla Road, Ragama, Sri Lanka<br>Ph: (94) 112665266, (94) 112665300<br>Email: asitades@gmail.com

(3) What will the study involve for me?

The discussion, which may be an individual interview or a group discussion, will cover information about your life and your health experience and views about the program. It will take approximately one hour and may occur at the same time as one of your study follow-up visits, or at a separate time, either at the hospital or at your home. Group discussions to discuss experiences with the intervention will usually include six to eight women. During an individual interview, if a question is asked that you do not want to answer, please say so. The researchers will then move on to another question. If a topic that you do not wish to discuss comes up during a group session, you do not need to contribute to that discussion.
All interviews and group discussions will be audiorecorded, and transcribed, and your name and personal details will be removed for analysis purposes. The data will be analysed by a small team of experienced researchers and will be stored on a secure, password-protected folder accessible only to the researchers. We seek permission to use quotes from your interview in our reports but none of this information will allow identification of an individual person.

(4) How much of my time will the study take?

Discussions conducted by the research team will take between 45 and 90 minutes. We appreciate your other commitments, and will fit this in with your schedule.

(5) Who can take part in the study?

Participants in the intervention group in the LIVING study can take part in this process evaluation.

(6) Do I have to be in the study? Can I withdraw from the study once I've started?

Your participation is voluntary and you are entirely free to decide whether or not to take part in this process evaluation. If you choose to take part, you are free to withdraw at any time, without giving any reason. If you choose not to take part, your medical care or legal rights will not be affected and you will continue to receive the usual care given by your doctors and other healthcare providers. Your decision whether to participate will not affect your current or future relationship with the researchers or any individuals or organizations related to this study.

Data that can be withdrawn:

If you wish, your information collected during individual interviews will be removed from our study records and will not be included in the study results, up to the point that we have analysed and published results.

Data that cannot be withdrawn:

Due to the nature of group discussions it will not be possible to delete the focus group discussion information from audio recordings. In this case, any information already collected will be kept in our records and may be included in the study results.

(7) Are there any risks or costs associated with being in the study?

Risks

There will be no costs associated with participating in the process evaluation. This process will not involve use of any drugs, medications or procedures. The risks of participating include:

(i) Giving up your time to participate in the study: This might cause some inconvenience but we will try to fit the discussions in to your schedule (e.g., hospital visits).

(ii) Psychological discomfort during the discussion: There is a risk that a topic could be raised that you may find upsetting. If this happens, please say so. The recording of the interview will be stopped immediately. The interview and recording will only start again if you agree, and a different topic will be discussed. If a topic that you do not wish to discuss comes up in a group discussion, you should avoid discussing it. You might worry afterwards about something that you said in the discussion. There is no need for this as everything is treated in strict confidence. All the recordings and written records will be made anonymous. If you are still worried, you can contact the researchers to discuss your concerns.

(8) Is this study associated with any financial benefits to researchers and affiliated organisations?

This study is not associated with any financial benefits to researchers and affiliated organisations.

(9) What happens if I suffer injury or complications as a result of the study?

We will be only collecting information from you by asking few questions and you will not be exposed to any drug, medication or procedure.

If you suffer any injuries or complications as a direct result of being in the study, you should contact the study investigator as soon as possible, who will assist you in arranging the appropriate medical treatment. In such cases, to cover medical care and/or compensation in case of any such events the sponsor(s) holds insurance policies.

If you have any questions concerning the availability of medical care or if you think you have experienced a research-related illness, injury or emergency, contact

Dr.__________________________ Phone: _________________________________

(Site level doctor details will be inserted later)

(10) Are there any benefits associated with being in the study?

You will have a chance to talk about your experiences, which you may find helpful. Participating in this discussion may not be of any other direct benefit to you. It is hoped that this process evaluation

will contribute to the revision and refinement of the Type 2 diabetes mellitus prevention intervention for women who have had gestational diabetes mellitus. We cannot, however, guarantee that it will be of direct benefit to you.
You will not be paid for taking part in this study. However, you will be reimbursed for reasonable travel expenses for study visits to the hospital, and provided refreshments at the discussion.

(11) What will happen to information about me that is collected during the study?

All information collected about you will be kept strictly confidential. Your initials and date of birth will be used as identifiers in the study. Only study monitors, auditors, and the Ethics Committee will be granted direct access to your records for verification of study procedures and/or data, without violating your confidentiality, to the extent permitted by the applicable laws. By signing a written Informed Consent Form, you authorise such access. Study findings may be published, but you will not be individually identified in these publications. All records and data relating to the study will be destroyed in a secure manner, 15 years after the end of this study, while protecting your confidentiality.

(12) Can I tell other people about the study?

Yes, you are welcome to tell other people about the study.

(13) What if I would like further information about the study?

When you have read this information, a member of the research team will be available to discuss it with you further and answer any questions you may have.

(14) Will I be told the results of the study?
You have a right to receive feedback about the overall results of this study. You can tell us that you wish to receive feedback by ticking the relevant box on the consent form. You will receive a summary of the research study results after the study is finished.

(15) What if I have a complaint or any concerns about the study?

Research involving humans is reviewed by an independent group of people called a Human Research Ethics Committee (HREC). The ethical aspects of this study have been approved by the HREC of *[to be replaced by relevant institution and protocol number].* As part of this process, we have agreed to carry out the study according to the Australian *National Statement on Ethical Conduct in Human Research (2007)*.

If you are concerned about the way this study is being conducted or you wish to make a complaint to someone independent from the study, please contact the university using the details outlined below. Please quote the study title and protocol number.

The Manager, Ethics Administration, University of Sydney:
Telephone: +61 2 8627 8176
Email: ro.humanethics@sydney.edu.au
Fax: +61 2 8627 8177 (Facsimile)

If you have any complaints regarding the research you may also contact the Patient Representative at your hospital/ the member secretary of the overseeing ethics committee, whose contact details are provided below.
[relevant ethics committee from below]

Details of local contacts in Bangladesh and Sri Lanka

**Bangladesh**	**Sri Lanka**
IRB Coordinator, Research Administration<br>Office of the Deputy Executive Director<br>Icddr,b, Mohakhali, Dhaka-1212<br>Mobile: +88 01711428989<br>Direct: +880-2-9827084<br>PABX: 9827001-10, Extn: 3206<br>Fax: +880-2-9827077<br>Email: salamk@icddrb.org<br>Web: www.icddrb.org	Secretary<br>Ethics Review Committee,<br>Faculty of Medicine<br>University of Kelaniya,<br>Thalagolla Road, Ragama, Sri Lanka<br>Email: ercmed@kln.ac.lk<br>Tel: +94112961142

Thank you for considering participation in this study!

LIVING – PROCESS EVALUATION OF LIVING STUDY INTERVENTION

Study Title: LIVING- Lifestyle InterVention IN Gestational diabetes

Study/Protocol Number: 1093171

Participant Initials: ________________________
Participant's Name: ________________________

Date of Birth/Age:________________________<br><br>*Please read the below mentioned statements and confirm your acceptance to participate in the process evaluation by giving an initial in the right hand side box against each statement.*	*Initial box(Subject)*<br>[ ]
(i) I confirm that I have read and understood the participant information statement dated …………….. for the above process evaluation and have had the opportunity to ask questions.	[ ]
(ii) I understand that my participation is voluntary and that I am free to withdraw at any time, without giving any reason, without my medical care or legal rights being affected.	[ ]
(iii) I understand that the Sponsor of the clinical trial, others working on the Sponsor's behalf, the Ethics Committee and the regulatory authorities will not need my permission to look at my health records both in respect of the current process evaluation and any further research that may be conducted in relation to it, even if I withdraw from the trial. I agree to this access. However, I understand that my identity will not be revealed in any information released to third parties or published.	[ ]
(iv) I agree not to restrict the use of any data or results that arise from this process evaluation provided such a use is only for scientific purpose(s).	[ ]
(v) I agree to take part in the above process evaluation.	[ ]

Subject/ legally acceptable representative	____________________<br>____________ Name	____________________<br>_<br>Signature/Thumb Impression	____________________<br>Date
Study Investigator	____________________<br>___________ Name	____________________<br>_____ Signature	____________________<br>Date
Impartial Witness	____________________<br>___________ Name	____________________<br>_____ Signature	____________________<br>Date

Would you like to receive feedback about the overall results of this process evaluation?

YES ☐ **NO** ☐

If you answered **YES**, please indicate your preferred form of feedback and address:

☐ Postal: ____

____

☐ Email: ____

Participant to complete:

Name: ____

Signature: ______________________________ Date: ____________________

Interviewer's name: ____

I have explained the nature and purpose of the process evaluation to the above participant, and have answered their questions.

Signature: ______________________________ Date: ____________________

10.6 ANNEXURE 6 - PROTOCOL SIGNATURE PAGE

Executive and Steering Committee Signature

I have read and approve this protocol for the study titled **Lifestyle InterVention IN Gestational Diabetes (LIVING): A lifestyle intervention program for the prevention of type 2 diabetes mellitus among South Asian women with gestational diabetes mellitus.**
I confirm that this study will be conducted according to all stipulations of the protocol, including all statements regarding confidentiality.

Name of Steering committee member	Signature	Date
Professor Nikhil Tandon<br>(PI and co-Chair)<br>All India Institute of Medical Sciences<br>New Delhi, India		
Professor Anushka Patel<br>(PI and co-Chair)<br>The George Institute for Global Health<br>Sydney, Australia		
Dr Dewan Alam<br>Centre for Global Health Research,<br>Toronto, Canada		
Professor Neerja Bhatla<br>All India Institute of Medical Sciences, New Delhi, India		
Associate Professor Laurent Billot<br>The George Institute for Global Health,<br>Sydney, Australia		
Dr Ankush Desai<br>Goa Medical College & Hospital<br>Goa, India		
Professor Asita de Silva<br>University of Kelaniya<br>Sri Lanka		
Dr Hema Divakar<br>Federation of Obstetrics and Gynaecology Societies of India		
Dr Sunil Fernando<br>University of Kelaniya<br>Colombo, Sri Lanka		
Professor Stephen Jan<br>The George Institute for Global Health<br>Sydney, Australia		
Associate Professor Rohina Joshi		

The George Institute for Global Health,<br>Sydney, Australia		
Dr Aliya Naheed<br>Icddr,b<br>Dhaka, Bangladesh		
Professor Arunasalam Pathmeswaran<br>University of Kelaniya, Sri Lanka		
Professor D Prabhakaran<br>Centre for Chronic Disease Control<br>New Delhi, India		
Dr D. Praveen<br>George Institute of Global Health<br>Hyderabad, India		
Professor Helena Teede<br>Monash University<br>Melbourne, Australia		
Professor Sophia Zoungas<br>Monash University,<br>Melbourne, Australia		

Participating Centre Investigator Signature

I have read this protocol, and agree that it contains all necessary details for carrying out the study as described. I will conduct this protocol as outlined therein, including all statements regarding confidentiality. I will make all reasonable efforts to complete the study within the time designated. I will provide copies of the protocol and access to all information furnished by the Steering Committee to study personnel under my supervision. I will discuss this material with them to ensure that they are fully informed about the study. I understand that the study may be terminated or enrollment suspended at any time by the Steering Committee, with or without cause, or by me if it becomes necessary to protect the best interests of the study participants.
I agree to conduct this study in full accordance with all applicable regulations

Investigator’s name (print):	
Address:	
Investigator’s signature:	
Date of signature:	

Lifestyle **I**nter**V**ention **IN** **G**estational Diabetes

Statistical Analysis Plan

Version: 1.0 (Final)

Date: 19 March 2021

Authors:

Amritendu Bhattacharya, The George Institute

Laurent Billot, The George Institute, UNSW Sydney

Anushka Patel, The George Institute, UNSW Sydney

Nikhil Tandon, All India Institute of Medical Sciences

Study identifiers:

Clinical Trials Registry of India No: CTRI/2017/06/008744;

Sri Lanka Clinical Trials Registry No: SLCTR/2017/001

ClinicalTrials.gov registry, Identifier: NCT03305939

Contents

1 Administrative information 4
1.1 Study identifiers 4
1.2 Revision history 4
1.3 Contributors to the statistical analysis plan 4
1.3.1 Roles and responsibilities 4
1.3.2 Approvals 5
2 Introduction 6
2.1 Purpose of this document 6
2.2 Study synopsis 6
2.3 Study population 6
2.3.1 Inclusion criteria 6
2.3.2 Exclusion criteria 6
2.4 Outcomes 7
2.4.1 Primary outcome 7
2.4.2 Secondary outcomes 7
2.5 Randomisation and blinding 7
2.6 Sample Size 7
3 Data handling and reporting rules 8
3.1 Randomization Errors 8
3.2 Analysis Sets 8
3.3 Baseline values 8
3.4 Handling of last (EOS) visit 8
3.5 Early study discontinuation/Lost to Follow-up 8
3.6 Calculation of durations 9
3.7 Definition of physical activity outcome 9
3.8 Definition of diet outcome 9
3.9 Coding of adverse events and concomitant medications 9
3.10 Handling of Missing data 9
3.11 Presentation 9
3.12 Quality assurance 10
4 Analysis details 11
4.1 General Principles 11
4.2 Interim analyses 11

4.3 Subject disposition 11
4.4 Baseline characteristics 11
4.5 Exposure to the intervention 12
4.6 Concomitant Medications 12
4.7 Diet and physical activity questionnaires 13
4.8 Healthcare utilisation 13
4.9 Analysis of the primary outcome 13
4.9.1 Descriptive analyses 13
4.9.2 Survival analysis 13
4.9.3 Poisson regression 14
4.9.4 Adjusted analyses 14
4.9.5 Subgroup analyses 14
4.9.6 Sensitivity analyses using HbA1c and FBG results 14
4.10 Secondary outcomes 15
4.10.1 Descriptive analyses 15
4.10.2 Linear models 15
4.10.3 Missing data handling 16
4.11 Analysis of other outcomes 16
4.11.1 Other continuous measurements 16
4.11.2 Serious Adverse Events 16
5 References 17
6 Appendix 18
6.1 Tables 18
6.2 Figures 31
6.3 Listings 34

1 Administrative information

1.1 Study identifiers

- Protocol: Version 6.0, Dated 13 May 2020
- Clinical Trials Registry of India No: CTRI/2017/06/008744;
- Sri Lanka Clinical Trials Registry No: SLCTR/2017/001
- ClinicalTrials.gov register Identifier: NCT03305939.

1.2 Revision history

Version	Date	Details
0.1 (draft)	05 February 2021	First version
0.2 (draft)	17 February 2021	
0.3 (draft)	18 February 2021	
0.4 (draft)	21 February 2021	
0.5 (draft)	26 February 2021	
0.6 (draft)	03 March 2021	
1.0 (final)	19 March 2021	Final version

1.3 Contributors to the statistical analysis plan

1.3.1 Roles and responsibilities

Name and ORCID	Affiliation	Role on study	SAP contribution
Amritendu Bhattacharya	The George Institute for Global Health	Statistician	Prepared initial draft
Laurent Billot	The George Institute for Global Health, UNSW Sydney	Study statistician	Reviewed every draft
Anushka Patel	The George Institute for Global Health, UNSW Sydney	Chief investigator	Reviewed every draft
Nikhil Tandon	All India Institute of Medical Sciences	Chief investigator	Reviewed final draft

1.3.2 *Approvals*

The undersigned have reviewed this plan and approve it as final. They find it to be consistent with the requirements of the protocol as it applies to their respective areas. They also find it to be compliant with ICH-E9 principles and, in particular, confirm that this analysis plan was developed in a completely blinded manner, i.e. without knowledge of the effect of the intervention(s) being assessed.

Mr Amritendu Bhattacharya

Amritendu 23/03/2021

Associate Professor Laurent Billot

Laurent Billot Digitally signed by Laurent Billot Date: 2021.03.19 16:58:04 +01'00'

Professor Anushka Patel

Anushka Patel Digitally signed by Anushka Patel Date: 2021.03.23 09:18:42 +11'00'

Professor Nikhil Tandon [illegible] 24/03/2021

2 Introduction

2.1 Purpose of this document

This statistical analysis plan (SAP) describes the statistical methods and data presentations to be used in the summary and analyses of data from Lifestyle Intervention in Gestational Diabetes (LIVING) study.

This document is based on the Protocol for LIVING, A lifestyle intervention program for the prevention of type 2 diabetes mellitus among South Asian women with gestational diabetes mellitus, Version 6.0 dated May 13, 2020. It describes the final analyses.

The document should be finalised before database lock and unblinding.

2.2 Study synopsis

The LIVING study aims to determine whether a resource- and culturally appropriate lifestyle intervention program in South Asian countries, provided to women with gestational diabetes (GDM) after childbirth, will reduce the incidence of worsening of glycemic status in a manner that is affordable, acceptable and scalable.

The study is an open-label parallel group pragmatic individual randomised controlled trial with blinded primary endpoint adjudication.

Eligible women were randomised to receive either usual care or the intervention in a 1:1 ratio. Following randomization, study visits captured data at months 6, 12,18, 24, 30, 36, 42 and 48 or up to the end-of-study (EOS) visit. All participants were required to have a minimum of 12 months follow-up. All EOS visits were scheduled between July 1st 2020 and January 31st 2021

2.3 Study population

The study is conducted in 20 sites in India, Bangladesh and Sri Lanka.

2.3.1 Inclusion criteria

- Absence of Type 2 diabetes mellitus (T2DM) at 3 to 18 months post-partum oral glucose tolerance test (OGTT).
- Met criteria for trial registration:
  - GDM diagnosed 24-34 weeks gestation through OGTT and using International Association of Diabetes and Pregnancy Study Groups (IADPSG) criteria *or* by other criteria outlined in the study protocol if OGTT was performed before 24 weeks of gestation or only a fasting plasma glucose level was obtained prior to 24 weeks gestation.

2.3.2 Exclusion criteria

- Confirmed case of T2DM
- Travel time to hospital more than 2 hours (unless individual circumstances will not impede hospital attendance for study visits)
- Lack of access to a mobile telephone
- Use of steroids during pregnancy other than for lung maturation of the baby

- Likelihood of moving residence in the next 3 years

2.4 Outcomes

2.4.1 Primary outcome

Change of glycaemic category, at or prior to final visit, defined as:

- A change from normal glucose tolerance to impaired fasting glucose (IFG) or impaired glucose tolerance (IGT)
- A change from normal glucose tolerance to T2DM;
- A change from IFG or IGT to T2DM

See detailed definition in Section 4.9.

2.4.2 Secondary outcomes

- Development of T2DM
- Fasting blood glucose (mg/dL)
- Body weight (kg)
- Waist circumference (cm)
- Systolic blood pressure (mmHg)
- Physical activity level (MET) – See definition in Section 3.7
- Diet (kcal) – See definition in Section 3.8

2.5 Randomisation and blinding

Randomisation occurred through a central, computer-based randomisation service, and was stratified by country, centre and use of insulin during pregnancy. By necessity, neither site investigators nor participants was blinded to allocation (intervention vs. usual care). However, all central study staff, statisticians, and outcome adjudicators were and will remain blinded until final database lock.

2.6 Sample Size

A sample size of 1414 women with GDM will provide 90% power ($2\alpha=0.05$) to detect a relative risk of 0.65, assuming that the control group cumulative incidence for change in glycaemic category will be 20% at 24 months and allowing for 20% of missing outcome data.

The initial power calculation was based on a chi-square test comparing two proportions at 24 months; however, to account for various study durations, the primary analysis will consist in a survival analysis of time to change in glycaemic category which is expected to have at least the same power as a comparison of proportions.

3 Data handling and reporting rules

3.1 Randomization Errors

The following 11 subjects were mis-randomised and will be excluded from the ITT analyses: S103023, S103137, S103139, S104012, S106008, S207007, S207012, S207021, S207026, S207031 and S416335.

3.2 Analysis Sets

All analyses will be based on the principle of intention-to-treat, i.e. by analysing subjects according to the group they were randomised to and regardless of compliance. The ITT will include all enrolled subjects who are randomised and have a baseline with respect to the efficacy evaluation of this study. Mis-randomised subjects will not be part of the ITT analysis.

Analyses of the primary and secondary outcomes will be repeated after including the 11 mis-randomised subjects. This will be indicated in the relevant sections of this SAP.

3.3 Baseline values

The last data available recorded at or prior to the randomisation visit will be used as baseline values unless otherwise specified.

3.4 Handling of last (EOS) visit

Given study participants entered the study at different times and were followed for various durations, the End of Study (EOS) visit will occur at different times for different participants.

For longitudinal analyses and descriptive analyses by visit, EOS visits will be re-allocated according to the planned visit schedule (i.e. Month 1, 6, 12, 18, 24 or 30). The allocation, based on the number of days between the date of randomisation and the EOS visit, will be done as follows:

- Month 6: number of days between 93 and 273 (inclusive)
- Month 12: number of days between 274 and 456 (inclusive)
- Month 18: number of days between 457 and 638 (inclusive)
- Month 24: number of days between 639 and 821 (inclusive)
- Month 30: number of days between 822 and 1003 (inclusive)
- Month 36: number of days between 1004 and 1186 (inclusive)

3.5 Early study discontinuation/Lost to Follow-up

Early termination is captured on the CRF and may be due to one of the following reasons:

- Withdrawal of Informed consent for follow-up visits
- Development of T2DM
- Occurrence of pregnancy
- Unable to contact or unable to follow-up

In case of early termination, a patient was expected to have an EOS visit completed at the time of termination. For those who did not terminate early, EOS visits with at least partial data collection were to be conducted after 1 July 2020.

3.6 Calculation of durations

All durations (e.g. time to event) will be calculated relative to the date of randomisation, if not specified otherwise.

Age will be calculated from the Date of birth to date of randomisation. For analyses of age involving patients who were not randomised, age will be calculated using the date of informed consent instead of the randomisation date.

3.7 Definition of physical activity outcome

Physical activity will be measured in terms of Metabolic Equivalent for Task (MET) derived from the Global Physical Activity Questionnaire (GPAQ).

The total number of METs in a typical week will be used as secondary outcome. Other measures of physical activity, not considered as secondary outcomes, include:

- Hours of moderate physical activity (total hours per week)
- Sedentary hours (time spend sitting or reclining) (average hours per day)
- Hours of sleep (average hours per night)

3.8 Definition of diet outcome

Diet will be measured as the total calorie intake derived from the INTAKE 24 questionnaire. It is calculated as follows:

- Calories from carbohydrate = 4 x grams of carbohydrate
- Calories from protein = 4 x grams of protein
- Calories from fat = 9 x grams of fat
- Total calorie intake = calories from carbohydrate + calories from protein + calories from fat

The calculated total calorie intake will be used as secondary outcome. Other measures of diet, not considered as secondary outcomes, will include:

- Protein intake (grams)
- Carbohydrate intake (grams)
- Fibre intake (grams)
- Fat intake (grams)
- Sodium intake (grams)

3.9 Coding of adverse events and concomitant medications

Serious Adverse Events (SAE) and Concomitant Medications (CM) will be categorised by medical experts who will provide corresponding standard terms for free texts used for both SAE and CM. Coding will be performed while blinded to the treatment allocation.

3.10 Handling of Missing data

The number of patients with available data will be reported. Missing or incomplete data will not be substituted/imputed, except where explicitly stated throughout the SAP.

3.11 Presentation

Listings will be sorted by unique subject identifier and analysis time point and/or date if applicable. Dates will be formatted as DDMMMYYYY. Partial dates will be presented on data listings as recorded on CRFs. Rounding for all variables will occur only as the last step, immediately prior to presentation in listings and tables.

No intermediate rounding will be performed on derived variables. The standard rounding practice of rounding numbers ending in 0-4 down and numbers ending 5-9 up will be employed. Every table, listing and figure will be produced with an electronic date stamp to document when it was produced.

3.12 Quality assurance

Double programming by 2 independent Statisticians/Statistical programmers will be done on all key analyses. Double programming may be replaced by code review if need arises. Discrepancies and/or data handling decisions will be adjudicated by a third statistician without access to the impacted results.

4 Analysis details

4.1 General Principles

Analyses will be performed using SAS Enterprise Guide version 8.3 or higher (SAS Institute Inc., Cary, NC, USA).

All variables will be analysed by descriptive statistical methods. The number of data available and missing data, mean, standard deviation, minimum, quartiles, median, and maximum will be calculated for continuous data. Frequency tables will be generated for categorical data. For categorical variables, the number and percentage of patients with a specific level of the variable will be presented.

All tests will be two-sided with a type-I error rate of 5%. Analyses of the primary outcome (change in category of glycaemia from normoglycaemia to IGT, IFG or T2DM or from IFG or IGT to T2DM) will be unadjusted for multiplicity; however, we will control the family-wise error rate across secondary outcomes using a Holm-Bonferroni correction [2]. No other multiplicity adjustment will be applied.

4.2 Interim analyses

No interim analysis was conducted.

4.3 Subject disposition

The flow of subjects through the trial will be described using a Consort diagram (see Figure 1).

Screening failures will be listed together with the reason for their exclusion from study and all available data (Date of informed consent, Reason for screen failure, Date of last visit).

At each visit, we will tabulate the number and proportions of patients according to the following categories:

- Whether the visit took place (yes vs no)
- Reason for no assessment (e.g. early discontinuation, LTFU) – see Section 3.5
- Visit type (in hospital, at home, by telephone)
- Oral glucose tolerance test (OGTT) data available when expected (yes or no)
- Haemoglobin A1c (HbA1c) data available when expected (yes or no)
- Fasting blood glucose (FBG) data available when expected (yes or no)

4.4 Baseline characteristics

The following variables will be summarized at baseline:

- Socio-demographic characteristics
  - Age
  - Primary language
  - Country
  - Religion
  - Highest educational qualification (3 categories will be created: up to primary including none, secondary and other higher degree (diploma/undergraduate and graduate)
  - Years of schooling

  - Current employment status
  - Current employment classification
  - Average household size of participant
- Risk Factors:
  - Reproductive history (Gravida, livebirths, time since delivery)
  - Family history
  - Obstetric history
  - Medical history (including history of hypertension and usage of hypertension medication)
  - Time since detection of GDM
  - Alcohol consumption
  - Tobacco consumption
- Vital Signs and anthropometric measures at baseline:
  - Height
  - Weight
  - Waist circumference
  - Waist circumference > 80 cm will be used to create a categorical variable separately
  - Hip circumference
  - Waist/Hip ratio
  - BMI
  - Blood pressure
  - Hypertension at baseline defined by SPB>=140 and DBP>=90
  - Heart rate
- Other Factors:
  - Use of insulin and pharmacotherapy during index pregnancy
  - Diet
  - Metabolic equivalent of task (MET) information at baseline(minutes/week)

4.5 Exposure to the intervention

The intervention will be summarised in terms of number of visits, type of visit (face-to-face, online or telephonic or other), if session was in group or individually, and other relevant details.

Given the complexity of the intervention and the fact that it only applies to participants randomised to the intervention group, compliance will be defined and summarised after we have analysed the details of the intervention.

To assess the potential effect of compliance, we will consider performing a complier average causal effect (CACE) analysis using both a propensity-weighted approach and a joint modelling approach [2].

4.6 Concomitant Medications

Categories of medications (e.g. blood pressure lowering, nutritional supplements) will be defined during the blind review. The number and proportion of patients taking a medication from each category at least once during the study will be reported. Differences in proportions between groups will be tested using Fisher's exact test.

4.7 Diet and physical activity questionnaires

Descriptive statistics will be presented for all diet and physical activity variables described in Sections 3.7 and 3.8 by visit and randomized group.

In addition longitudinal mean plots and repeated-measure generalised linear mixed models will be used to assess differences between groups over time as described in Sections 4.10.1 and 4.10.2.

More detailed analyses will be considered in secondary papers and are not described in this SAP.

4.8 Healthcare utilisation

The number of events as well as the number and proportion of participants accessing each service (e.g. hospital, pharmacy, etc.) at least once during the study will be reported overall and by treatment group. Differences in proportions will be tested using Fisher's exact test.

More detailed analyses of healthcare utilisation including costs will be conducted later and are not described in this SAP.

4.9 Analysis of the primary outcome

The primary outcome is a change of glycaemic category, at or prior to final visit defined as:

- A change from Normal glucose tolerance (NGT) to impaired fasting glucose (IFG) or impaired glucose tolerance (IGT), or T2DM
- A change from IFG or IGT to T2DM

This will be diagnosed using ADA criteria as given below:

- Normal glucose tolerance (<100 mg/dL fasting and <140 mg/dL post-glucose load)
- Impaired glucose tolerance (<100 mg/dL fasting and 140-199 mg/dL post-load)
- Impaired fasting glucose (100-125 mg/dL fasting and <140mg/dL post-load)
- Type 2 diabetes (≥126 mg/dL fasting or ≥200 mg/dL post-load)

4.9.1 Descriptive analyses

The number and proportion of subjects in each glycaemic category will be reported by group and by visit according to the six following categories:

1) Normoglycaemia
2) IFG
3) IGT
4) IFG + IGT
5) T2DM
6) Unknown glycaemic status

This will be done both using a table and a stacked bar chart.

4.9.2 Survival analysis

Change in glycaemic category for primary outcome as defined in section 2.4.1 will be analysed as a time to event calculated as the number of days between randomisation and the day of the event. For subjects with no event, data will be censored on the day of the last OGTT test results.

Survival by treatment arm will be described using a Kaplan-Meier plot with 95% confidence interval will be reported by treatment arm. If available, median time to event as well as quartiles will be derived from the Kaplan-Meier estimates. Censoring will be done at time of last OGTT test.

A Cox model will be used to estimate the Hazard ratio of the two arms (Intervention compared to Control). The Cox model will include the randomised group as well as prior use of insulin as fixed effects and study site as a random effect [4]. We will visually assess the proportional hazard assumption. SAS Proc PHREG will be used.

4.9.3 Poisson regression

We will conduct a sensitivity analysis of the primary endpoint using Poisson regression i.e. using a generalised linear mixed model assuming a Poisson distribution and a Log link. The dependent variable will be a binary indicator (yes vs no) indicating whether the primary outcome occurred for each subject. Covariates will include the randomised group and prior use of insulin as fixed effects and a random effect of study site. The model will adjust for exposure (in years) defined as time to event or censoring, using the same definition as the one used for the survival analysis. The Poisson model will be used to estimate the yearly event rate and 95% confidence interval in each group. The effect of the intervention will be estimated as the rate ratio and its 95% confidence interval.
SAS GLIMMIX procedure will be used.

4.9.4 Adjusted analyses

Both the Cox and Poisson models will be repeated after adding the following covariates to the model: prediabetes (yes/on), BMI (underweight, normal, overweight, obese), time since GDM-affected pregnancy (as a continuous variable, measured in months) and age (as a continuous variable, measured in years).

4.9.5 Subgroup analyses

The Cox and Poisson models described in sections 4.9.2 and 4.9.3 will be conducted according to the following baseline subgroups:

- Baseline glycaemic status: prediabetes vs normoglycaemia
- Gravida: 1 vs >1
- Country: Bangladesh, India, Sri Lanka
- Body mass index (BMI): underweight (<18), normal (18-25), overweight (>25-30), obese (>30)
- Insulin use in index pregnancy: yes vs no
- Time since GDM-affected pregnancy: above vs below or equal to median
- Age: above vs below or equal to median

This will be done by adding the corresponding subgroup variable to the main Cox and Poisson models as a fixed effect together with its interaction with the randomised group. Heterogeneity by subgroup will be quantified by the p-value associated with the interaction term. Results will be presented on forest plots.

4.9.6 Sensitivity analyses using HbA1c and FBG results

Analyses described in Sections 4.9.1, 4.9.2 and 4.9.3 will be repeated while using FBG and HbA1c values where an OGTT was not performed as scheduled during a follow-up visit. Censoring will occur

at the time of the last relevant measure of glycaemia. For visits where an OGTT was performed, glycaemic categories will be defined according to the definition of Section 4.9. Otherwise, the following hierarchical approach will be used:

1) For those with both HbA1c and FBG data available:
    a. T2DM: **HbA1c >=6.5% or FBG >=126 mg/dL**
    b. Normal glucose tolerance: **HbA1c <5.7% and FBG <100 mg/dL**
    c. Pre-diabetes: everyone else

2) For those with only HbA1c data available:
    a. T2DM: **HbA1c >=6.5%**
    b. Normal glucose tolerance: **HbA1c <5.7%**
    c. Pre-diabetes: everyone else

3) For those with only FBG data available:
    a. T2DM: **FBG >=126 mg/dL**
    b. Normal glucose tolerance: **FBG <100 mg/dL**
    c. Pre-diabetes: everyone else

4.10 Secondary outcomes

Secondary outcomes include the following variables:

- Development of T2DM
- Fasting blood glucose
- Body weight
- Waist circumference
- Systolic blood pressure
- Physical activity level defined as the average daily MET (see definition in Section 3.7)
- Diet defined as the total 24-hour calorie intake (see definition in Section 3.8)

Development of T2DM will be analysed in the same way as the primary outcome, i.e. using survival analysis and Poisson regression as described in sections 4.9.2 and 4.9.3. No adjusted or subgroup analyses will be applied to this outcome except for a subgroup analysis according to baseline glycaemic status (normoglycaemia vs pre-diabetes).

4.10.1 Descriptive analyses

Each continuous secondary outcome (i.e. all but development of T2DM) will be summarised by visit for each group and overall using mean, standard deviation, median and quartiles. Longitudinal plots will be used to display raw means and 95% confidence intervals by randomised group and by visit.

4.10.2 Linear models

Repeated-measure generalised linear mixed models will be used to assess differences between groups over time for all continuous secondary outcomes. Models will include the randomised group, the visit and the group by visit interaction, use of insulin during pregnancy and the baseline value of the outcome as fixed effects. Random intercepts by subject and by study centre will be added to the model. Within-subject correlations as well as within-site correlations will be assumed to follow an exchangeable structure.

The effect of the intervention will be estimated as the adjusted mean difference at each visit and overall (all visits combined) together with its 95% confidence interval. A p-value from the likelihood ratio test will only be calculated for the overall difference based on all visits combined.

4.10.3 Missing data handling

Missing data will not be imputed; however, any subject with at least one post-baseline value will be included in the longitudinal models.

4.11 Analysis of other outcomes

4.11.1 Other continuous measurements

Continuous measurements not already included as primary or secondary outcomes include HbA1c, heart rate and hip circumference. All will be analysed using the same approach as the one applied to secondary outcomes (see Sections 4.10.1 and 4.10.2).

4.11.2 Serious Adverse Events

Serious Adverse Events (SAE) will be categorised during a blind review (see Section 3.9). Events will be summarised as the number of events as well as the number and percentage of subjects experiencing a given event at least once by category and by treatment arm. Differences in the proportion of subjects experiencing a category of event at least once will be tested using Fisher's exact test.

5 References

1. Gupta Y, Kapoor D, Josyula LK, et al. A lifestyle intervention programme for the prevention of Type 2 diabetes mellitus among South Asian women with gestational diabetes mellitus [LIVING study]: protocol for a randomized trial. Diabet Med. 2019 Feb;36(2):243-251. doi: 10.1111/dme.13850. Epub 2018 Nov 29. PMID: 30368898.
2. Stuart EA, Jo B. Assessing the sensitivity of methods for estimating principal causal effects. Stat Methods Med Res. 2015;24(6):657-674. doi:10.1177/0962280211421840
3. Holm S. A simple sequentially rejective multiple test procedure. Scand J Stat 1979; 6: 65-70
4. Kahan BC. Accounting for centre-effects in multicentre trials with a binary outcome — when, why, and how? BMC Med Res Methodol 2014; 14: 20. doi: 10.1186/1471-2288-14-20.

6 Appendix

6.1 Tables

Table 1. Screen failures by reason

	Total (N count)
**Reason 1**	
**Reason 2**	
**Etc.**	

Table 2. Overall participant disposition

	All Subjects	Intervention	Control
**Screened**	xx	xx	xx
**Registered**	xx	xx	xx
**Randomised**	xx	xx	xx
**Randomised and eligible**	xx (xx.x)	xx (xx.x)	xx (xx.x)
**EOS completed**	xx (xx.x)	xx (xx.x)	xx (xx.x)
**Stopped the study early**	xx (xx.x)	xx (xx.x)	xx (xx.x)
**Due to pregnancy**	xx (xx.x)	xx (xx.x)	xx (xx.x)
**Due to development of T2DM**	xx (xx.x)	xx (xx.x)	xx (xx.x)
**Lost to follow-up**	xx (xx.x)	xx (xx.x)	xx (xx.x)

Note: Percentages are calculated based on the number of participants randomised. The category 'EOS completed' can include participants who stopped early due to pregnancy and development of T2DM.

Table 3. Baseline characteristics

Characteristics	Intervention	Control	Total
**Age at randomization (years)**<br>N<br>mean (SD)<br>Q1 Q2 Q3<br>Min, Max			
**Primary language**<br>Tamil<br>Hindi<br>Telugu<br>Bengali<br>Sinhalese<br>Marathi<br>Konkani<br>..<br>..			
**Country**<br>Bangladesh<br>India<br>Srilanka			
**Religion**<br>Hindu<br>Muslim<br>Christian<br>Buddhist<br>Other			
**Highest Educational Qualification**<br>Primary (up to Primary including none)<br>Secondary<br>Other Higher degrees(Diploma/UG/Graduate)			
**Number of Years of Schooling**<br>N<br>mean (SD)<br>Q1 Q2 Q3<br>Min, Max			
**Current Employment Status**<br>Employed<br>Unemployed			
**Current employment classification**<br>xx			

**Average Household Size**<br>N<br>mean (SD)<br>Q1 Q2 Q3<br>Min, Max			
**POG at diagnosis of GDM**<br>N<br>mean (SD)<br>Q1 Q2 Q3<br>Min, Max			
**Early GDM (<=24 weeks)**<br>Yes<br>No			
**Gravida>1**<br>Yes<br>No			
**Livebirth>=1**<br>Yes<br>No			
**Time Since Delivery**<br>N<br>mean (SD)<br>Q1 Q2 Q3<br>Min, Max			
**Family History of Diabetes**<br>Yes<br>No			
**Family History of Hypertension**<br>Yes<br>No			
**Past History of Gestational Diabetes**<br>Yes<br>No			
**Use of Insulin in Index Pregnancy**<br>Yes<br>No			
**Past Use of Insulin**<br>Yes<br>No			
**Past Use of Medication**<br>Yes<br>No			
**Alcohol Consumption**<br>Yes<br>No			
**Tobacco Use**<br>Yes<br>No			
**Height**<br>N			

mean (SD)<br>Q1 Q2 Q3<br>Min, Max			
**Weight**<br>N<br>mean (SD)<br>Q1 Q2 Q3<br>Min, Max			
**BMI**<br>Underweight<br>Normal<br>Overweight<br>Obese			
**HbA1c (%)**<br>N<br>mean (SD)<br>Q1 Q2 Q3<br>Min, Max			
**Glucose 0 min (mmol/L) at baseline postpartum (mg/dl)**<br>N<br>mean (SD)<br>Q1 Q2 Q3<br>Min, Max			
**Glucose 120 min (mmol/L) at baseline postpartum (mg/dl)**<br>N<br>mean (SD)<br>Q1 Q2 Q3<br>Min, Max			
**Waist Circumference**<br>N<br>mean (SD)<br>Q1 Q2 Q3<br>Min, Max			
**WC(>80cm)**<br>Yes<br>No			
**Hip Circumference**<br>N<br>mean (SD)<br>Q1 Q2 Q3<br>Min, Max			
**Waist/Hip Ratio**<br>N<br>mean (SD)<br>Q1 Q2 Q3<br>Min, Max			
**Hypertension**<br>Yes<br>No			

**Hypertension Concomitant Medication**<br>Yes<br>No			
**HTN%**<br>Yes<br>No			
**Systolic Blood Pressure (mmHg)**<br>N<br>mean (SD)<br>Q1 Q2 Q3<br>Min, Max			
**SBP GE 140**<br>Yes<br>No			
**Diastolic Blood Pressure (mmHg)**<br>N<br>mean (SD)<br>Q1 Q2 Q3<br>Min, Max			
**DBP GE 90**<br>Yes<br>No			
**Heart Rate**<br>N<br>mean (SD)<br>Q1 Q2 Q3<br>Min, Max			
**Calorie Intake (Kcal/day)**<br>N<br>mean (SD)<br>Q1 Q2 Q3<br>Min, Max			
**Carbohydrates (grams/day)**<br>N<br>mean (SD)<br>Q1 Q2 Q3<br>Min, Max			
**Proteins (grams/day)**<br>N<br>mean (SD)<br>Q1 Q2 Q3<br>Min, Max			
**Total Fat (grams/day)**<br>N<br>mean (SD)<br>Q1 Q2 Q3<br>Min, Max			
**Total Fibre (grams/day)**<br>N<br>mean (SD)			

Q1 Q2 Q3<br>Min, Max			
**Sodium (grams/day)**<br>N<br>mean (SD)<br>Q1 Q2 Q3<br>Min, Max			
**MET (weekly total)**<br>N<br>mean (SD)<br>Q1 Q2 Q3<br>Min, Max			
**Moderate activity (hours/week)**			
**Sedentary Behaviour (hours/day)**<br>N<br>mean (SD)<br>Q1 Q2 Q3<br>Min, Max			
**Sleep Hours (hours/day)**<br>N<br>mean (SD)<br>Q1 Q2 Q3<br>Min, Max			
**Alcohol**<br>Yes<br>No			
**Tobacco Consumption**<br>Yes<br>No			

Table 4. Participant disposition by visit

	Intervention N=()	Control N=()	Total N=()
**Randomisation**			
**Assessed**	**xxx (xx.x%)**	**xxx (xx.x%)**	**xxx (xx.x%)**
With OGTT data	xxx (xx.x%)	xxx (xx.x%)	xxx (xx.x%)
No OGTT but HbA1c and FBG data	xxx (xx.x%)	xxx (xx.x%)	xxx (xx.x%)
With HbA1c data only	xxx (xx.x%)	xxx (xx.x%)	xxx (xx.x%)
With FBG data only	xxx (xx.x%)	xxx (xx.x%)	xxx (xx.x%)
**Not assessed**	**xxx (xx.x%)**	**xxx (xx.x%)**	**xxx (xx.x%)**
Completed expected FU	xxx (xx.x%)	xxx (xx.x%)	xxx (xx.x%)
Discontinued due to pregnancy	xxx (xx.x%)	xxx (xx.x%)	xxx (xx.x%)
Discontinued due to T2DM	xxx (xx.x%)	xxx (xx.x%)	xxx (xx.x%)
LTFU / unknown reason	xxx (xx.x%)	xxx (xx.x%)	xxx (xx.x%)
**Month 6**			
**Assessed**	**xxx (xx.x%)**	**xxx (xx.x%)**	**xxx (xx.x%)**
With OGTT data	xxx (xx.x%)	xxx (xx.x%)	xxx (xx.x%)
No OGTT but HbA1c and FBG data	xxx (xx.x%)	xxx (xx.x%)	xxx (xx.x%)
With HbA1c data only	xxx (xx.x%)	xxx (xx.x%)	xxx (xx.x%)
With FBG data only	xxx (xx.x%)	xxx (xx.x%)	xxx (xx.x%)
**Not assessed**	**xxx (xx.x%)**	**xxx (xx.x%)**	**xxx (xx.x%)**
Completed expected FU	xxx (xx.x%)	xxx (xx.x%)	xxx (xx.x%)
Discontinued due to pregnancy	xxx (xx.x%)	xxx (xx.x%)	xxx (xx.x%)
Discontinued due to T2DM	xxx (xx.x%)	xxx (xx.x%)	xxx (xx.x%)
LTFU / unknown reason	xxx (xx.x%)	xxx (xx.x%)	xxx (xx.x%)
**EOS**			

Programming note: include all scheduled visits at Randomisation, Month 6, 12, 18, 24, 30, 36, 42, 48 and EOS.

Table 5. Description of vital Signs and Anthropometric measurements by visit

	Intervention N mean (SD)	Control N mean (SD)	Total N mean (SD)
**SBP (mmHg)**			
Randomisation	xxx xxx.x (xxx.xx)	xxx xxx.x (xxx.xx)	xxx xxx.x (xxx.xx)
Month 6	xxx xxx.x (xxx.xx)	xxx xxx.x (xxx.xx)	xxx xxx.x (xxx.xx)
Month 12	xxx xxx.x (xxx.xx)	xxx xxx.x (xxx.xx)	xxx xxx.x (xxx.xx)
Etc.			
**Heart Rate (bpm)**			
**Weight (Kg)**			
**Waist Circumference (cm)**			
**Hip Circumference (cm)**			
**HbA1c (cm)**			
**FBG (mg/dL)**			

Programming note: Data to be summarised by visit after reallocation of the EOS visit

Table 6. Description of diet and physical activity measures

	Intervention N mean (SD)	Control N mean (SD)	Total N mean (SD)
**Total calories**			
Randomisation	xxx xxx.x (xxx.xx)	xxx xxx.x (xxx.xx)	xxx xxx.x (xxx.xx)
Month 6	xxx xxx.x (xxx.xx)	xxx xxx.x (xxx.xx)	xxx xxx.x (xxx.xx)
Month 12	xxx xxx.x (xxx.xx)	xxx xxx.x (xxx.xx)	xxx xxx.x (xxx.xx)
Etc.			
**Protein (grams)**			
**Etc.**			
**Sleep (hours)**			

Programming note: See Sections 3.7 and 3.8 for list of variables. Data to be summarised by visit after reallocation of the EOS visit

Table 7. Glycaemic category distribution by Visit

Visit / Glycaemic category	Intervention	Control	Total
**Randomisation**	xx (xx.x%)	xx (xx.x%)	xx (xx.x%)
Normoglycaemia	xx (xx.x%)	xx (xx.x%)	xx (xx.x%)
Pre-diabetes	xx (xx.x%)	xx (xx.x%)	xx (xx.x%)
IFG alone	xx (xx.x%)	xx (xx.x%)	xx (xx.x%)
IGT alone	xx (xx.x%)	xx (xx.x%)	xx (xx.x%)
IFG + IGT	xx (xx.x%)	xx (xx.x%)	xx (xx.x%)
T2DM	xx (xx.x%)	xx (xx.x%)	xx (xx.x%)
Unknown	xx	xx	xx
**Month 6**	xx (xx.x%)	xx (xx.x%)	xx (xx.x%)
Normoglycaemia	xx (xx.x%)	xx (xx.x%)	xx (xx.x%)
Pre-diabetes	xx (xx.x%)	xx (xx.x%)	xx (xx.x%)
IFG alone	xx (xx.x%)	xx (xx.x%)	xx (xx.x%)
IGT alone	xx (xx.x%)	xx (xx.x%)	xx (xx.x%)
IFG + IGT	xx (xx.x%)	xx (xx.x%)	xx (xx.x%)
T2DM	xx (xx.x%)	xx (xx.x%)	xx (xx.x%)
Unknown	xx	xx	xx
**Month 12**	xx (xx.x%)	xx (xx.x%)	xx (xx.x%)
Normoglycaemia	xx (xx.x%)	xx (xx.x%)	xx (xx.x%)
Pre-diabetes	xx (xx.x%)	xx (xx.x%)	xx (xx.x%)
IFG alone	xx (xx.x%)	xx (xx.x%)	xx (xx.x%)
IGT alone	xx (xx.x%)	xx (xx.x%)	xx (xx.x%)
IFG + IGT	xx (xx.x%)	xx (xx.x%)	xx (xx.x%)
T2DM	xx (xx.x%)	xx (xx.x%)	xx (xx.x%)
Unknown	xx	xx	xx
**Etc.**			

Programming note: include all scheduled visits after reallocation of EOS. Corresponding bar chart in Figure section. Do not include those with unknown/missing status in the denominator when calculating percentages.

Table 8. Change in glycaemic status

Outcome / analysis	Intervention N=xxx	Control N=xxx	Hazard/rate Ratio (95% CI)	p-value
**Change in glycaemic category**				
Using OGTT only	***xx events***	***xx events***		
Survival analysis [1]	x.xx (x.xx to x.xx)	x.xx (x.xx to x.xx)	x.xx (x.xx to x.xx)	0.xxx
Adjusted			x.xx (x.xx to x.xx)	0.xxx
Poisson regression	x.xx (x.xx to x.xx)	x.xx (x.xx to x.xx)	x.xx (x.xx to x.xx)	0.xxx
Adjusted	x.xx (x.xx to x.xx)	x.xx (x.xx to x.xx)	x.xx (x.xx to x.xx)	0.xxx
Using HbA1c and FBG	***xx events***	***xx events***		
Survival analysis	x.xx (x.xx to x.xx)	x.xx (x.xx to x.xx)	x.xx (x.xx to x.xx)	0.xxx
Adjusted				0.xxx
Poisson regression	x.xx (x.xx to x.xx)	x.xx (x.xx to x.xx)	x.xx (x.xx to x.xx)	0.xxx
Adjusted	x.xx (x.xx to x.xx)	x.xx (x.xx to x.xx)	x.xx (x.xx to x.xx)	0.xxx
**Development of T2DM**				
Using OGTT only	***xx events***	***xx events***		
Survival analysis	x.xx (x.xx to x.xx)	x.xx (x.xx to x.xx)	x.xx (x.xx to x.xx)	0.xxx
Poisson regression	x.xx (x.xx to x.xx)	x.xx (x.xx to x.xx)	x.xx (x.xx to x.xx)	0.xxx
Using HbA1c and FBG	***xx events***	***xx events***		
Survival analysis	x.xx (x.xx to x.xx)	x.xx (x.xx to x.xx)	x.xx (x.xx to x.xx)	0.xxx
Poisson regression	x.xx (x.xx to x.xx)	x.xx (x.xx to x.xx)	x.xx (x.xx to x.xx)	0.xxx

1. Primary analysis

Programming Note: refer to sections 4.9.2, 4.9.3, 4.9.4 and 4.9.6 for analysis details

Table 9.Mixed model results for continuous secondary outcomes

Outcome / visit	Intervention Mean (SE)	Control Mean (SE)	Mean difference (95% CI)	p-value
Systolic blood pressure				
Month 6	xxx.x (xxx.xx)	xxx.x (xxx.xx)	x.xx (x.xx to x.xx)	
Month 12	xxx.x (xxx.xx)	xxx.x (xxx.xx)	x.xx (x.xx to x.xx)	
Month 18	xxx.x (xxx.xx)	xxx.x (xxx.xx)	x.xx (x.xx to x.xx)	
Month 24	xxx.x (xxx.xx)	xxx.x (xxx.xx)	x.xx (x.xx to x.xx)	
Month 30	xxx.x (xxx.xx)	xxx.x (xxx.xx)	x.xx (x.xx to x.xx)	
Month 36	xxx.x (xxx.xx)	xxx.x (xxx.xx)	x.xx (x.xx to x.xx)	
Overall	xxx.x (xxx.xx)	xxx.x (xxx.xx)	x.xx (x.xx to x.xx)	0.xxx
Body weight				
Month 6	xxx.x (xxx.xx)	xxx.x (xxx.xx)	x.xx (x.xx to x.xx)	
Month 12	xxx.x (xxx.xx)	xxx.x (xxx.xx)	x.xx (x.xx to x.xx)	
Month 18	xxx.x (xxx.xx)	xxx.x (xxx.xx)	x.xx (x.xx to x.xx)	
Month 24	xxx.x (xxx.xx)	xxx.x (xxx.xx)	x.xx (x.xx to x.xx)	
Month 30	xxx.x (xxx.xx)	xxx.x (xxx.xx)	x.xx (x.xx to x.xx)	
Month 36	xxx.x (xxx.xx)	xxx.x (xxx.xx)	x.xx (x.xx to x.xx)	
Overall	xxx.x (xxx.xx)	xxx.x (xxx.xx)	x.xx (x.xx to x.xx)	0.xxx

Notes: Estimates obtained from a repeated-measure linear mixed model including the randomised group, the visit and the group by visit interaction, use of insulin during pregnancy and the baseline value of the outcome as fixed effects. Random intercepts by subject and by study centre will be added to the model. Within-subject correlations as well as within-site correlations will be assumed to follow an exchangeable structure

Programming note: do for the following outcomes:

- *Fasting blood glucose*
- *Body weight*
- *Waist circumference*
- *Systolic blood pressure*
- *METs (see definition in Section 3.7)*
- *Total 24-hour calorie intake (see definition in Section 3.8)*

Table 10.Mixed model results for other continuous measures

Outcome / visit	Intervention Mean (SE)	Control Mean (SE)	Mean difference (95% CI)	p-value
HbA1c				
Month 6	xxx.x (xxx.xx)	xxx.x (xxx.xx)	x.xx (x.xx to x.xx)	
Month 12	xxx.x (xxx.xx)	xxx.x (xxx.xx)	x.xx (x.xx to x.xx)	
Month 18	xxx.x (xxx.xx)	xxx.x (xxx.xx)	x.xx (x.xx to x.xx)	
Month 24	xxx.x (xxx.xx)	xxx.x (xxx.xx)	x.xx (x.xx to x.xx)	
Month 30	xxx.x (xxx.xx)	xxx.x (xxx.xx)	x.xx (x.xx to x.xx)	
Month 36	xxx.x (xxx.xx)	xxx.x (xxx.xx)	x.xx (x.xx to x.xx)	
Overall	xxx.x (xxx.xx)	xxx.x (xxx.xx)	x.xx (x.xx to x.xx)	0.xxx
Heart Rate				
Month 6	xxx.x (xxx.xx)	xxx.x (xxx.xx)	x.xx (x.xx to x.xx)	
Month 12	xxx.x (xxx.xx)	xxx.x (xxx.xx)	x.xx (x.xx to x.xx)	
Month 18	xxx.x (xxx.xx)	xxx.x (xxx.xx)	x.xx (x.xx to x.xx)	
Month 24	xxx.x (xxx.xx)	xxx.x (xxx.xx)	x.xx (x.xx to x.xx)	
Month 30	xxx.x (xxx.xx)	xxx.x (xxx.xx)	x.xx (x.xx to x.xx)	
Month 36	xxx.x (xxx.xx)	xxx.x (xxx.xx)	x.xx (x.xx to x.xx)	
Overall	xxx.x (xxx.xx)	xxx.x (xxx.xx)	x.xx (x.xx to x.xx)	0.xxx
**Etc.**				

Notes: Estimates obtained from a repeated-measure linear mixed model including the randomised group, the visit and the group by visit interaction, use of insulin during pregnancy and the baseline value of the outcome as fixed effects. Random intercepts by subject and by study centre will be added to the model. Within-subject correlations as well as within-site correlations will be assumed to follow an exchangeable structure

Programming note: do for the following outcomes:

- *HbA1c*
- *Heart rate*
- *Hip circumference*
- *Moderate activity hours*
- *Sedentary hours*
- *Sleep hours*
- *Protein intake*
- *Fibre intake*
- *Fat intake*
- *Carbohydrate intake*
- *Sodium intake.*

Table 11. Concomitant Medications

	Intervention	Control	Total	P-value
**Category 1**	xx (xx%)	xx (xx%)	xx (xx%)	0.xxx
**Category 2**	xx (xx%)	xx (xx%)	xx (xx%)	0.xxx
**Category 3**	xx (xx%)	xx (xx%)	xx (xx%)	0.xxx
**Etc.**	xx (xx%)	xx (xx%)	xx (xx%)	0.xxx

Programming notes: proportion of subjects experiencing a category of event at least once will be tested using Fisher's exact test

Table 12. Healthcare utilization

	Intervention # events / # participants (%)	*Control # events / # participants (%)*	*Total # events / # participants (%)*	*p-value**
**Government/public hospital**	xxx / xx (xx.x%)	xxx / xx (xx.x%)	xxx / xx (xx.x%)	0.xxx
**Community health centre**	xxx / xx (xx.x%)	xxx / xx (xx.x%)	xxx / xx (xx.x%)	0.xxx
**Private hospital/clinic**	xxx / xx (xx.x%)	xxx / xx (xx.x%)	xxx / xx (xx.x%)	0.xxx
**Primary health centre**	xxx / xx (xx.x%)	xxx / xx (xx.x%)	xxx / xx (xx.x%)	0.xxx
**Subcentre**	xxx / xx (xx.x%)	xxx / xx (xx.x%)	xxx / xx (xx.x%)	0.xxx
**Mobile clinic**	xxx / xx (xx.x%)	xxx / xx (xx.x%)	xxx / xx (xx.x%)	0.xxx
**Government dispensary**	xxx / xx (xx.x%)	xxx / xx (xx.x%)	xxx / xx (xx.x%)	0.xxx
**NGO/Trust hospital/clinic**	xxx / xx (xx.x%)	xxx / xx (xx.x%)	xxx / xx (xx.x%)	0.xxx
**Pharmacy/Shop**	xxx / xx (xx.x%)	xxx / xx (xx.x%)	xxx / xx (xx.x%)	0.xxx

Programming notes: differences in the proportion of subjects experiencing a category of event at least once will be tested using Fisher's exact test.

6.2 Figures

Figure 1: Cumulative monthly enrolment of Screening, Registration and Randomisation

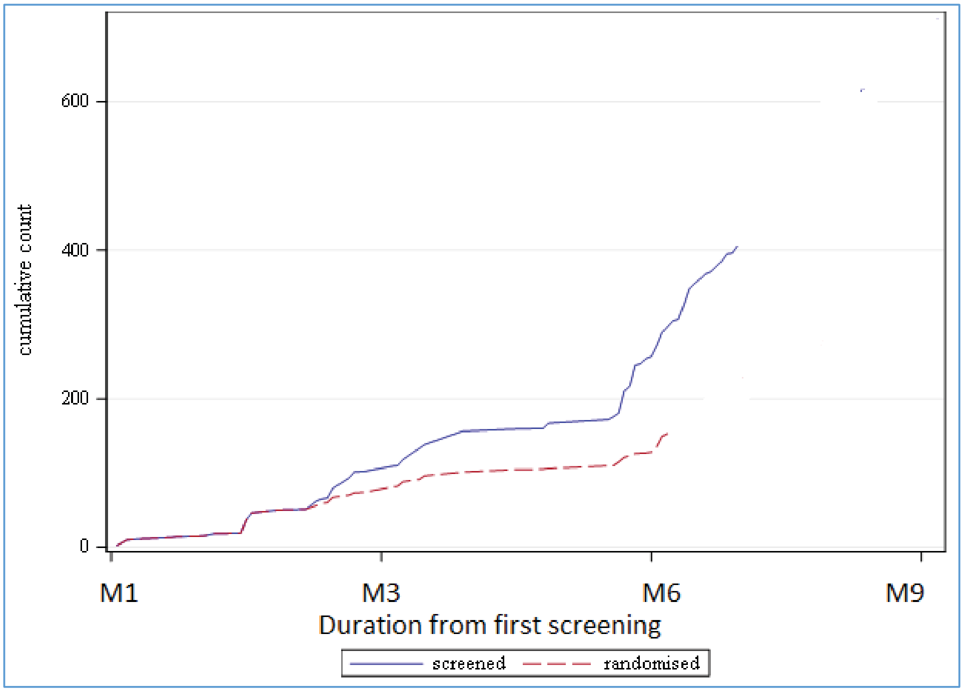

Figure 2: Consort diagram

A consort diagram showing the number of subjects screened, registered and randomised together with key data (e.g. OGTT, HbA1C and FBG) at each visit will be shown as in the Figure below extracted from the UMPIRE paper.

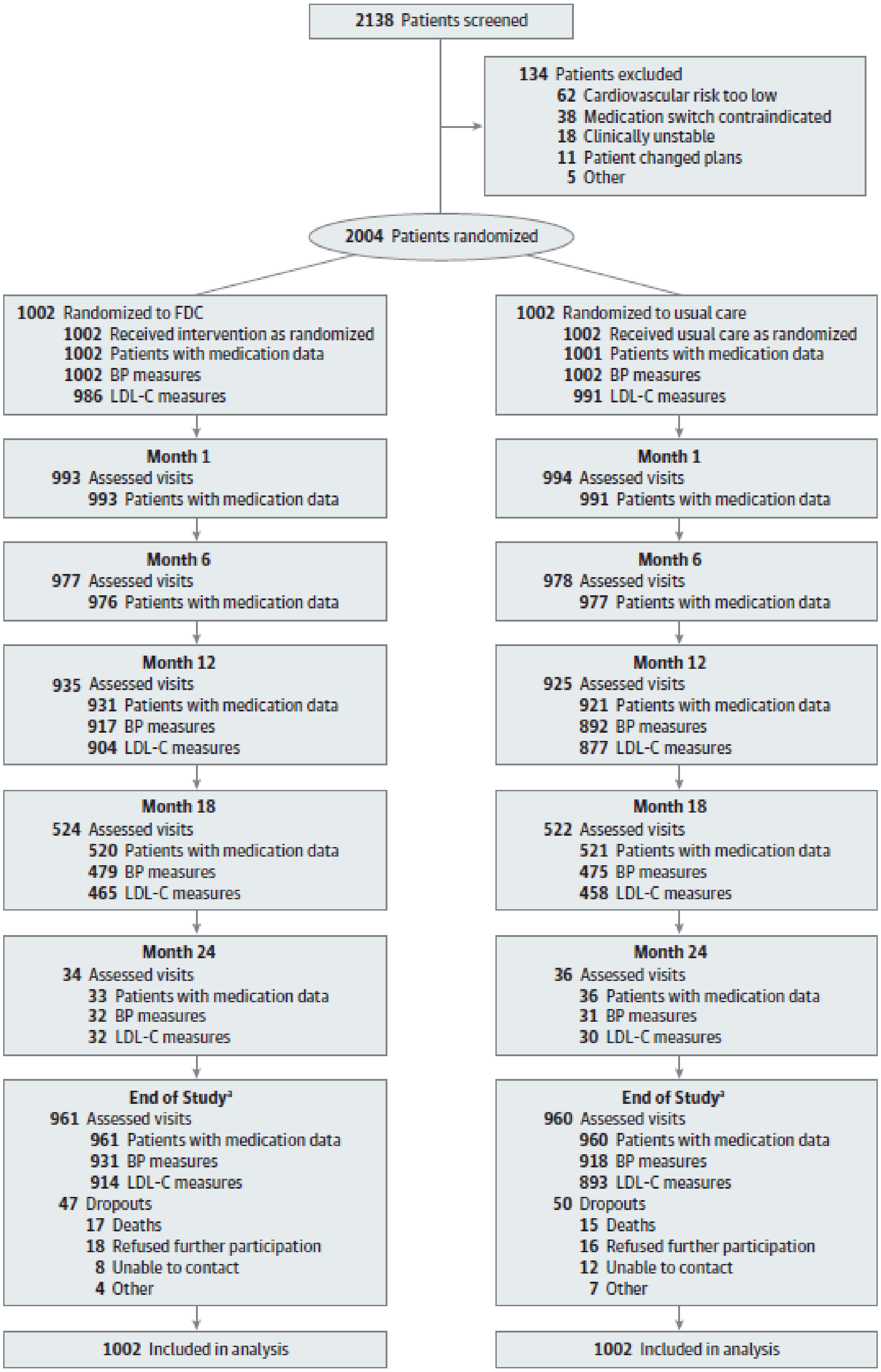

Figure 3: Result of OGTT among randomised subjects by country and overall and visit

Note: This figure will consist of a stacked bar chart with one bar per visit and the following four categories (summing to 100% within each bar): normal glycaemic status, pre-diabetes, T2DM or unknown status. Each group will be on a separate panel with both panels presented side by site. The figure will be repeated using 6 categories i.e. by splitting the 'pre-diabetes' category into the 3 following: (i) IFG only (ii) IGT only (iii) IFG + IGT. Show n and % in each category on the plot.

Figure 4: Survival plot of time to event (change in glycaemic status from normal to prediabetes / T2DM or prediabetes to T2DM) by treatment

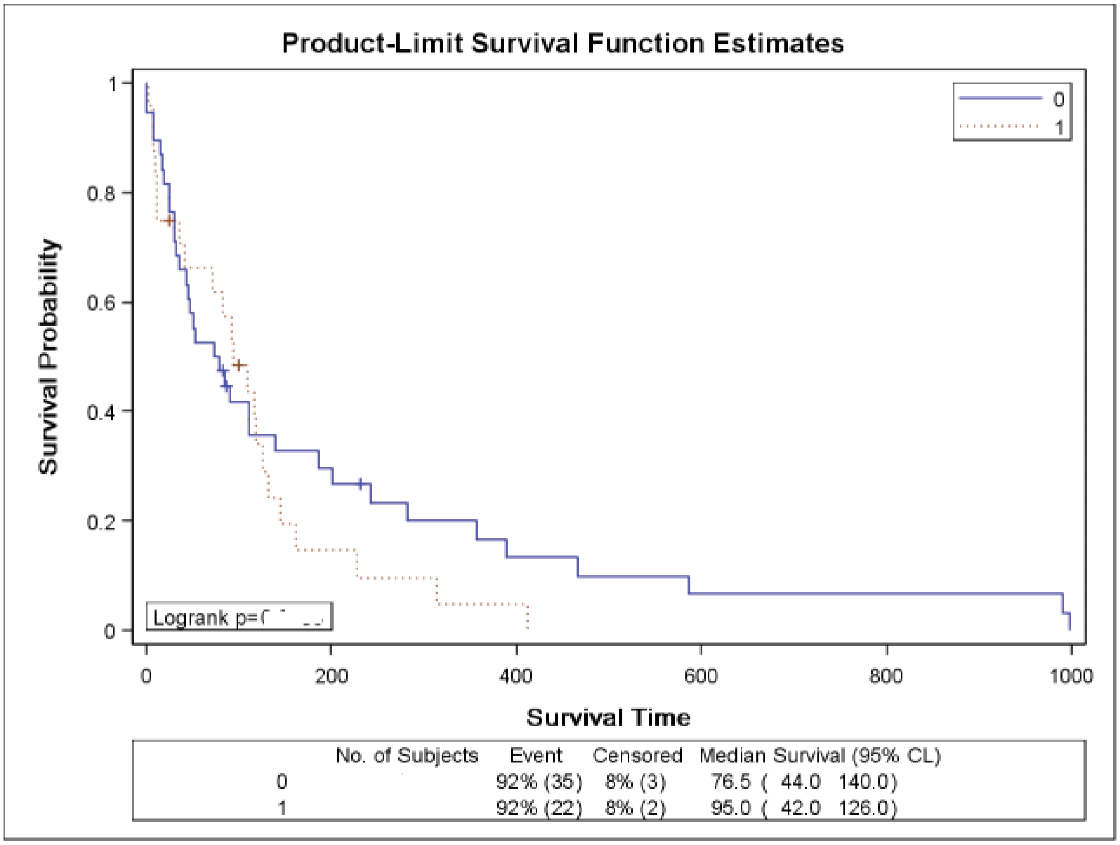

Note: Show number of participants at risk at every six-monthly visit. Repeat for sensitivity analyses using HbA1c and FBG measurements as well as for time to T2DM outcome (4 figures in total).

Figure 5: Longitudinal plot of xxx per visit

Note: repeat for all secondary continuous outcomes (see Section 4.10) and other continuous measures (see Section 4.11.1) collected at each visit

Figure 6: Forest plot of subgroup analyses

6.3 Listings

Listing 1: Serious Adverse Events

Subject ID	SAE	Start Date	End Date	Severity	Determination of Severity	Treatment	Outcome	Relationship
