# Supplementary material for: Effects of a Lifestyle Intervention to Prevent Deterioration in Glycemic Status Among South Asian Women With Recent Gestational Diabetes: A Randomized Clinical Trial
Source: JAMA Netw Open. 2022 Mar 2;5(3):e220773. doi: 10.1001/jamanetworkopen.2022.0773 (PMC8892226; doi:10.1001/jamanetworkopen.2022.0773)
Supplement: Supplement 2. — eMethods 1. Methods for Diagnosis of Gestational Diabetes eMethods 2. Detailed Description of Planned Lifestyle Intervention Program eTable 1. Baseline Characteristics by Country eTable 2. Participant Disposition by Visit eTable 3. Sensitivity Analyses for Primary Outcome eTable 4. Effects of Intervention on Additional Outcomes eFigure 1. Primary Outcome Determination for Sensitivity Analysis eFigure 2. Postdelivery Glycemic Status of Registered Participants eFigure 3. Intervention Fidelity eFigure 4. Changes In Continuous Secondary Outcomes Over Time eFigure 5. Kaplan-Meier Plot for Development of Type 2 Diabetes eFigure 6. Prespecified Subgroup Analyses for Primary Outcome [file jamanetwopen-e220773-s002.pdf]

Supplemental Online Content

Tandon N, Gupta Y, Kapoor D, et al; LIVING Collaborative Group. Effects of a lifestyle intervention to prevent deterioration in glycemic status among South Asian women with recent gestational diabetes: a randomized clinical trial. *JAMA Netw Open*. 2022;5(3):e220773. doi:10.1001/jamanetworkopen.2022.0773

eMethods 1. Methods for Diagnosis of Gestational Diabetes

eMethods 2. Detailed Description of Planned Lifestyle Intervention Program

eTable 1. Baseline Characteristics by Country

eTable 2. Participant Disposition by Visit

eTable 3. Sensitivity Analyses for Primary Outcome

eTable 4. Effects of Intervention on Additional Outcomes

eFigure 1. Primary Outcome Determination for Sensitivity Analysis

eFigure 2. Postdelivery Glycemic Status of Registered Participants

eFigure 3. Intervention Fidelity

eFigure 4. Changes In Continuous Secondary Outcomes Over Time

eFigure 5. Kaplan-Meier Plot for Development of Type 2 Diabetes

eFigure 6. Prespecified Subgroup Analyses for Primary Outcome

This supplemental material has been provided by the authors to give readers additional information about their work.

© 2022 Tandon N et al. *JAMA Network Open.*

eMethods 1. Methods for Diagnosis of Gestational Diabetes

	**Criteria for diagnosis of GDM**	**N (%) randomized participants**
Scenario 1: OGTT at 24-34 weeks of gestation (IADPSG criteria)	Fasting plasma glucose 92 to 125 mg/dl; and/or 1hr glucose ≥ 180 mg/dl; and/or 2hr glucose 153 to 199 mg/dl.	1421 (87.6%)*
Scenario 2: OGTT at <24 weeks gestation	Fasting plasma glucose 92 to 125 mg/dl; and/or 1hr glucose ≥ 180 mg/dl; and/or 2hr glucose 153 to 199 mg/dl.	
	On pharmacological therapy	132 (8.2%)
	Repeat OGTT at 24-34 weeks gestation, with fasting plasma glucose 92 to 125 mg/dl; and/or 1hr glucose ≥ 180 mg/dl; and/or 2hr glucose 153 to 199 mg/dl.	1 (0.06%)
	Fasting plasma glucose at 24-34 weeks gestation 92 to 125 mg/dL.	3 (0.18%)
Scenario 3: only fasting plasma glucose available at <24 weeks gestation	Fasting plasma glucose 92 to 125 mg/dL.	
	On pharmacological therapy	44 (2.8%)
	Repeat OGTT at 24-34 weeks gestation, with fasting plasma glucose 92 to 125 mg/dl; and/or 1hr glucose ≥ 180 mg/dl; and/or 2hr glucose 153 to 199 mg/dl.	0
	Fasting plasma glucose at 24-34 weeks gestation 92 to 125 mg/dL	0

*1 participant was recruited based on fasting plasma glucose alone as she had been given a 100g glucose load.

© 2022 Tandon N et al. *JAMA Network Open.*

eMethods 2. Detailed Description of Planned Lifestyle Intervention Program

A. Facilitator training
B. Group sessions - 4 sessions
C. Phone calls - monthly
D. Mobile phone text or voice messages - twice a week
E. Intensification individual counseling sessions - 2

A. Facilitator training

Program facilitators (auxiliary nurse midwives (or equivalent)) underwent two days of program specific training before they started interacting with the study participants. This included both theory and practical components, and motivational interviewing techniques. The training module included developing interpersonal skills, sound knowledge of evidence-based practice, an understanding of health, health behaviour, nutrition, and physical activity, and knowledge and experience in communicating with a variety of participants. Following the initial training, facilitators attended monthly meetings for ongoing support and opportunities to discuss barriers and develop strategies to optimise the delivery of the program. Facilitators were provided with implementation protocols, data-collection templates, and delivery and implementation resources such as scripts, photographs, and a selection of strategies to assist with behaviour change.

B. The LIVING intervention group sessions are an amalgamation of:

i. Core messages focusing on the delivery of basic knowledge about key factors contributing to increased blood glucose levels and associations with diet and physical activity.
ii. Live demonstrations that allow translation of information discussed during group sessions in real life scenarios e.g.: reducing fat and sugar content in common foods, home based physical activities
iii. Goal setting activities aimed at motivating participants to set short term goals considering their current threshold limits and relating to the following 5 areas – limiting fat intake, particularly saturated fat; limiting sugar and salt intake; increasing fibre (fruits/vegetables) intake; increasing physical activity; and weight reduction and maintenance.
iv. Baby- and family-centred messaging to provide social and mental health support.

© 2022 Tandon N et al. *JAMA Network Open.*

Session	Themes	Focus	Activities
1	Introduction and general knowledge of GDM and T2DM	• GDM and consequences for mother and baby<br>• T2DM and pre- diabetes<br>• Glucose and insulin interaction<br>• Signs and symptoms of T2DM<br>• Complications of T2DM<br>• Risk factors for T2DM	**String up:** An activity to build rapport where participants are asked to identify their problems and others in the group who face similar issues are grouped. Possible solutions are discussed within these smaller groups.<br>**I am here!:** Anthropometric measurements and blood glucose values are recorded in participant manuals. Based on the body mass index, personal weight maintenance or reduction goals for are defined to check future progress.
	Managing risk factors for T2DM	• Decreasing T2DM<br>• Understanding energy balance<br>• Causes of weight gain<br>• Misconceptions about healthy diet and physical activity<br>• Practical recommendations	
	Overcoming challenges and meeting goals	• Outline of the programme	
	Focus on the baby	• Timing and frequency of complementary feeding<br>• Importance and examples of complementary food<br>• Preparation of feeds	
2	Weight Management	• Weight loss<br>• Weight gain	**Demonstration of commonly consumed foods for hidden fats and sugars:** Participants are sensitised about the amount of fat and sugars in common foods by reading and interpreting food labels. Chips may be
	Balanced diet	• The three-food group concept<br>• Analysing your plate	

© 2022 Tandon N et al. *JAMA Network Open.*

		• Macronutrients<br>• Micronutrients	burned to demonstrate hidden fat in a small piece of food.<br>**Modifying common day-to-day food practices:**<br>**Analyse your plate:** Plate models with ½ fruits and vegetables, ¼ pulses/meats and ¼ portion cereals are demonstrated.<br>**Recipe modification:** Ways to make recipes healthier by addition/substitution of certain ingredients are discussed**.**<br>**Making healthy food choices:** Different eating scenarios - at home, in a market, at a restaurant, at parties/weddings – and how healthier choices can be made, are discussed.<br>**Reading food labels:** Reading and interpretation of food labels is done to raise awareness of how much sugar, saturated fat and salt can be consumed unknowingly.<br>**Setting dietary goals:** Recording of current dietary practices and how these can be modified, in what settings and how, is facilitated. Practical examples short-term behaviour change goals are provided.
	Changing your diet	• Adopting healthy cooking practices<br>• Recipe modification<br>• Mindful eating	
	Goal setting	• Dietary goals	
	Baby nutrition	• Important nutrients	
3	Defining physical activity	• Benefits of physical activity<br>• Types of physical activity<br>• Intensity of physical activity	**Understanding barriers and facilitators to dietary goals set in session 2:** Participants list their barriers and facilitators to achieving their personal dietary goals. Participants are encouraged to discuss and find potential solutions with the group.<br>**Analyse your activity:** All activities done in a typical day are listed and arranged in a pyramid. Sedentary activities are placed at the top and discouraged, while
	Physical inactivity	• Excuses	
	Opportunities for physical activity	• Work, travel, home<br>• Leisure time	

© 2022 Tandon N et al. *JAMA Network Open.*

	Examples of home-based exercises	• Simple exercise for the home	active lifestyle (climbing stairs, brisk walking, playing with children) are encouraged and placed at the base.<br>**Simple home-based exercises:** Exercises are demonstrated and practiced with participants encouraged to continue at home and involve friends and family.<br>**Setting activity goals:** Similar physical activity goal setting exercise as done in session 2. This focuses on setting goals for being active to achieve personal weight reduction/maintenance goals. Inactive participants are encouraged to begin with 10-15 minutes of activity and then slowly increase levels to achieve the goals.
	Engaging with family	• Being active with the baby<br>• Family support for being active	
	Goal setting	• Physical activity goals	
	Stress management	• Understanding stress<br>• Postnatal depression<br>• Coping with stress<br>• Getting help with stress<br>• Relationships	
4	Recap of previous sessions	• Glucose and insulin<br>• Preventing T2DM	**Two truths and a lie:** Participants are encouraged to identify continuing barriers in achieving their goals and consider potential solutions.<br>**What is your score?:** Progress against goals is recorded.<br>**Understanding barriers and facilitators to physical activity goals set in session 3:** Similar approach to the dietary goals exercise.<br>**How healthy you choose when eating out?** Participants are given different eating scenarios and tested on making healthy choices.<br>**How much do I weigh today?**
	Recap of diet session	• Nutrients<br>• Healthy eating out<br>• Changing diet in practice	
	Recap of physical activity session	• Benefits of physical activity<br>• Increasing incidental physical activity	
	Maintaining behaviour change	• Practical tips	
	Health as a family priority	• Infant health and hygiene<br>• Self-hygiene<br>• Other general health advice	

© 2022 Tandon N et al. *JAMA Network Open.*

C. **Phone calls:** The facilitator initiates the call but the content is participant driven. Probing questions are used include asking how she is doing, and whether she has been able to achieve individual goals. As needed, further coaching and re-emphasis of core messages is done.
D. **Mobile phone text or voice messages:** Each participant receives automated text or voice messages (based on their preference) focused on the themes covered in the group sessions.
E. **Intensification sessions:** These are individual counseling sessions that further probe barriers to making healthy changes in diet and physical activity and explore individual level solutions to overcome such barriers.

© 2022 Tandon N et al. *JAMA Network Open.*

eTable 1. Baseline Characteristics by Country

	Bangladesh (N=371)	India (N=776)	Sri Lanka (450)
Age, mean (SD), y	29.4 (5.0)	31.0 (4.5)	31.9 (5.1)
Formal education, mean (SD), y	11.6 (4.2)	13.4 (3.5)	12.6 (2.6)
Currently employed, No. (%)	49 (13.2%)	179 (22.9%)	75 (16.7%)
Gravida, median (IQR)	2 (2, 3)	2 (1, 3)	2 (1, 3)
Time since delivery, mean (SD), months	5.1 (3.6, 6.7)	6.9 (5.3, 9.3)	6.6 (5.5, 7.7)
Prior history of non-index pregnancy gestational diabetes mellitus, No. (%)	25 (6.7%)	60 (7.7%)	36 (8.0%)
Insulin use during index pregnancy, No. (%)	63 (17.0%)	121 (15.5%)	50 (11.1%)
Family history of diabetes in first degree relatives, No. (%)	201 (54.2%)	341 (43.7%)	233 (51.8%)
Family history of hypertension in first degree relatives, No. (%)	210 (56.6%)	210 (26.9%)	190 (42.2%)
Self-reported tobacco use, No. (%)	0 (0%)	0 (0%)	0 (0%)
Self-reported alcohol use, No. (%)	0 (0%)	18 (0.3%)	56 (12.4%)
Body weight, mean (SD), y	63.6 (10.6)	63.5 (12.8)	63.3 (11.6)
Body mass index, mean (SD), kg/m^2	27.5 (4.4)	26.2 (4.9)	26.5 (4.5)
Waist circumference, mean (SD), cm	92.5 (11.8)	89.2 (12.2)	87.9 (11.0)
Haemoglobin A_{1c}, median (IQR)	5.5 (5.2, 5.8)	5.4 (5.1, 5.7)	5.5 (5.2, 5.8)
Fasting blood glucose, mean (SD), mg/dL	95.7 (10.7)	91.0 (10.9)	95.5 (10.3)
Glycemic status, No. (%)			
Normoglycemia	208 (56.1%)	536 (68.7%)	257 (57.1%)
IFG only	65 (17.5%)	94 (12.1%)	81 (18.0%)
IGT only	43 (11.6%)	100 (12.8%)	45 (10.0%)
IFG and IFT	55 (14.8%)	50 (6.4%)	67 (14.9%)
Systolic blood pressure, mean (SD), mmHg	115 (13)	113 (9)	111 (13)
Diastolic blood pressure, mean (SD), mmHg	75 (11)	74 (8)	74 (9)
Total Calorie intake, mean (SD), Kcal/day	1866 (561)	1670 (565)	1588 (520)
Total Carbohydrate intake, mean (SD), g/day	304 (94)	257 (91)	260 (85)
Total Protein intake, mean (SD), g/day	72 (41)	57 (26)	50 (21)
Total Fat intake, mean (SD), g/day	40 (19)	46 (25)	39 (23)
Total Fibre intake, mean (SD), g/day	12 (10)	18 (10)	9 (6)
Total Sodium intake, mean (SD), g/day	7.8 (4.1)	6.4 (3.0)	7.5 (4.5)
Moderate physical activity, mean (SD), minutes/day	264.1 (122.6)	258.8 (155.5)	236.7 (108.4)
Sedentary activity, mean (SD), minutes /day	169.3 (99.7)	300.5 (280.7)	119.0 (117.9)
Sleep duration, mean (SD), minutes/day	440.8 (73.9)	400.5 (81.6)	370.7 (72.6)

© 2022 Tandon N et al. *JAMA Network Open.*

eTable 2. Participant Disposition by Visit

	Intervention			Usual Care	
**Randomisation**	**Number at risk = 800**			**Number at risk = 801**	
	# Events	1		# Events	3
	T2DM	1		T2DM	2
	Worsening (excl. T2DM)	0		Worsening (excl. T2DM)	1
	# Censored	152		# Censored	118
	EOS (no event)	10		EOS (no event)	11
	Pregnancy	59		Pregnancy	39
	LTFU	60		LTFU	41
	Missing/other	23		Missing/other	27
	Total	153		Total	121
**Month 6**	**Number at risk = 647**			**Number at risk = 680**	
	# Events	45		# Events	55
	T2DM	16		T2DM	20
	Worsening (excl. T2DM)	29		Worsening (excl. T2DM)	35
	# Censored	56		# Censored	69
	EOS (no event)	44		EOS (no event)	58
	Pregnancy	6		Pregnancy	5
	LTFU	0		LTFU	4
	Missing/other	6		Missing/other	2
	Total	101		Total	124
**Month 12**	**Number at risk = 546**			**Number at risk = 556**	
	# Events	101		# Events	101
	T2DM	29		T2DM	29
	Worsening (excl. T2DM)	72		Worsening (excl. T2DM)	72
	# Censored	212		# Censored	210
	EOS (no event)	198		EOS (no event)	191
	Pregnancy	12		Pregnancy	10
	LTFU	1		LTFU	5
	Missing/other	1		Missing/other	4
	Total	313		Total	311
**Month 18**	**Number at risk = 233**			**Number at risk = 245**	
	# Events	34		# Events	41
	T2DM	13		T2DM	22
	Worsening (excl. T2DM)	21		Worsening (excl. T2DM)	19
	# Censored	106		# Censored	111
	EOS (no event)	106		EOS (no event)	111
	Pregnancy	0		Pregnancy	0
	LTFU	0		LTFU	0
	Missing/other	0		Missing/other	0
	Total	140		Total	152

© 2022 Tandon N et al. *JAMA Network Open.*

**Month 24**	**Number at risk = 93**			**Number at risk = 93**	
	# Events	19		# Events	15
	T2DM	13		T2DM	6
	Worsening (excl. T2DM)	6		Worsening (excl. T2DM)	9
	# Censored	59		# Censored	63
	EOS (no event)	59		EOS (no event)	63
	Pregnancy	0		Pregnancy	0
	LTFU	0		LTFU	0
	Missing/other	0		Missing/other	0
	Total	78		Total	78
**Month 30**	**Number at risk = 15**			**Number at risk = 15**	
	# Events	3		# Events	2
	T2DM	2		T2DM	1
	Worsening (excl. T2DM)	1		Worsening (excl. T2DM)	1
	# Censored	10		# Censored	13
	EOS (no event)	10		EOS (no event)	13
	Pregnancy	0		Pregnancy	0
	LTFU	0		LTFU	0
	Missing/other	0		Missing/other	0
	Total	13		Total	15
**Month 36**	**Number at risk = 2**			**Number at risk = 0**	
	# Events	1		# Events	0
	T2DM	0		T2DM	0
	Worsening (excl. T2DM)	1		Worsening (excl. T2DM)	0
	# Censored	1		# Censored	0
	EOS (no event)	1		EOS (no event)	0
	Pregnancy	0		Pregnancy	0
	LTFU	0		LTFU	0
	Missing/other	0		Missing/other	0
	Total	2		Total	0

© 2022 Tandon N et al. *JAMA Network Open.*

eTable 3. Sensitivity Analyses for Primary Outcome

		**HR (95% CI) or RR (95% CI)**	**p-value**
Primary survival analysis		0.92 (0.76 to 1.12)	0.42
Adjusted survival analysis		0.91 (0.75 to 1.10)	0.34
Poisson analysis		0.95 (0.78 to 1.15)	0.58
Adjusted Poisson analysis		0.92 (0.76 to 1.12)	0.40
Analyses using additional measures of glycaemia*			
	Survival analysis	1.06 (0.89 to 1.25)	0.53
	Adjusted survival analysis	1.02 (0.86 to 1.21)	0.80
	Poisson analysis	1.04 (0.88 to 1.24)	0.62
	Adjusted Poisson analysis	0.99 (0.83 to 1.17)	0.86
Analyses including 11 ineligible randomised participants			
	Survival analysis	0.92 (0.76 to 1.12)	0.40
	Adjusted survival analysis	0.91 (0.75 to 1.10)	0.32
	Poisson analysis	0.95 (0.78 to 1.15)	0.57
	Adjusted Poisson analysis	0.92 (0.76 to 1.12)	0.40

Adjusted models included the following variables: age, pre-diabetes (yes/no), body mass index category, and time since GDM-affected pregnancy.

*HbA_{1c} and/or fasting plasma glucose

© 2022 Tandon N et al. *JAMA Network Open.*

eTable 4. Effects of Intervention on Additional Outcomes

	N	Intervention Mean (SE)	Usual care Mean (SE)	Mean difference (95% CI)	p-value
Change in heart rate (bpm)	1401	0.5 (0.7)	-0.4	0.9 (0.0 to 1.9)	0.07
Change in diastolic blood pressure (mmHg)	1401	1.7 (0.6)	2.1 (0.6)	-0.4 (-1.3 to 0.5)	0.40
Change in HbA_1c (%)	1420	0.1 (0.03)	0.1 (0.03)	0.0 (-0.1 to 0.1)	0.94
Change in hip circumference (cm)	1395	-0.4 (0.5)	-0.9 (0.5)	0.5 (-0.3 to 1.4)	0.21
Change in daily moderate physical activity (minutes/day)	1406	-23.5 (20.6)	-25.0 (20.6)	1.5 (-10.2 to 13.2)	0.80
Change in daily sedentary activity (minutes/day)	1495	-6.4 (33.0)	-3.8 (32.9)	-2.6 (-15.2 to 9.9)	0.68
Change in sleep duration (minutes/day)	1495	18.3 (8.3)	16.3 (8.2)	2.0 (-4.7 to 8.7)	0.57
Change in total carbohydrate intake (grams/day)	1419	-51 (10)	-47 (10)	-4.0 (-12.9 to 4.8)	0.37
Change in total protein intake (grams/day)	1419	-10 (3)	-8 (3)	-2.4 (-4.6 to -0.3)	0.03
Change in total fat intake (grams/day)	1419	-3 (3)	-2 (3)	-1.4 (-3.8 to 1.0)	0.25
Change in total fibre intake (grams/day)	1419	-0.9 (0.9)	-0.7 (0.9)	-0.2 (-0.9 to 0.5)	0.62
Change in total sodium intake (grams/day)	1419	-1.0 (0.1)	-0.8 (0.1)	-0.1 (-0.4 to 0.1)	0.20

Note: A Cox model was used to analyse time to development of T2DM. All other models consist in longitudinal linear mixed models including all data available collected during follow-up. The N column indicates the number of subjects contributing to each analysis

© 2022 Tandon N et al. *JAMA Network Open.*

eFigure 1. Primary Outcome Determination for Sensitivity Analysis

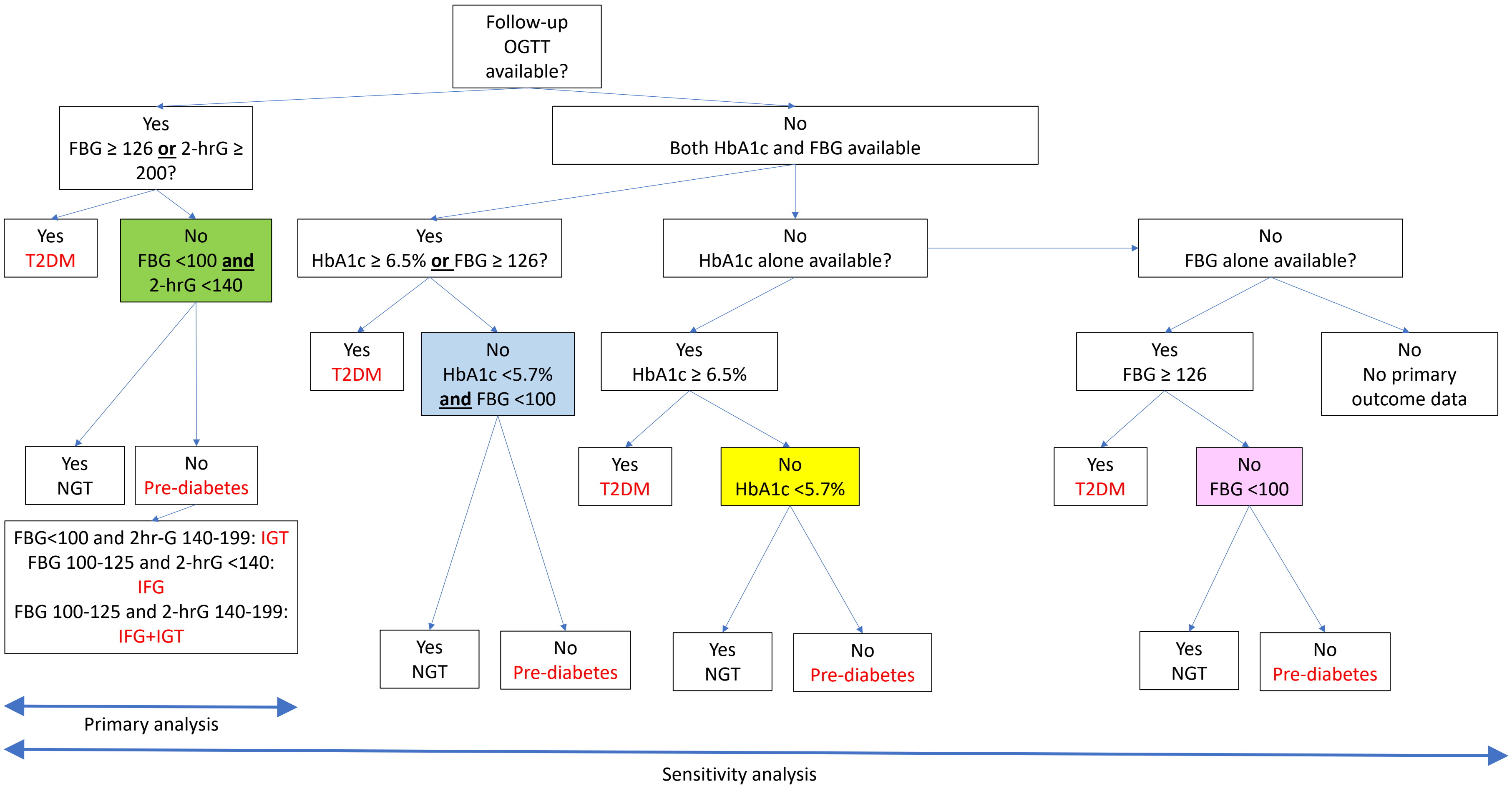

© 2022 Tandon N et al. *JAMA Network Open.*

eFigure 2. Postdelivery Glycemic Status of Registered Participants

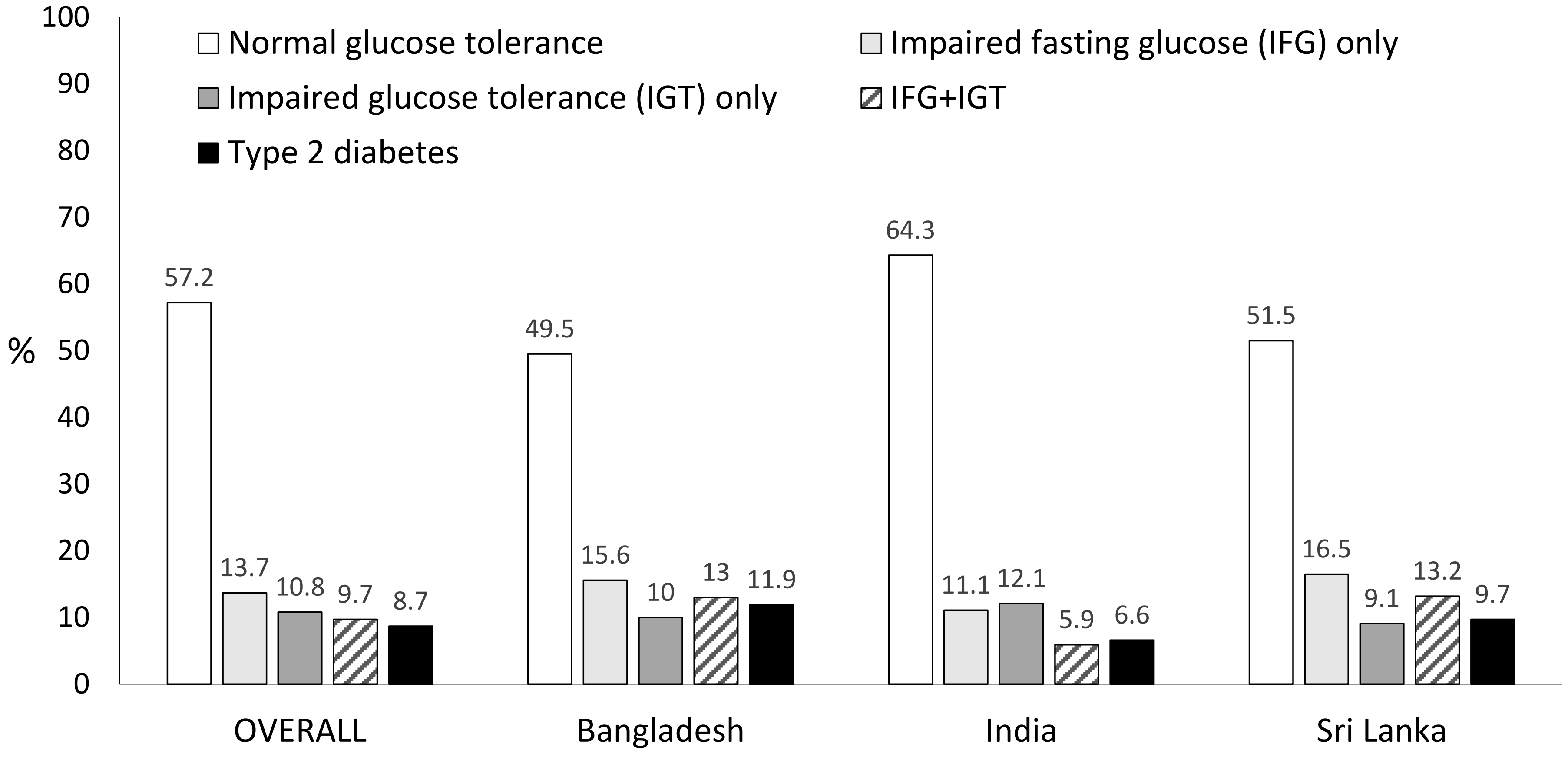

OGTT data missing for 2 registered participants

© 2022 Tandon N et al. *JAMA Network Open.*

eFigure 3. Intervention Fidelity

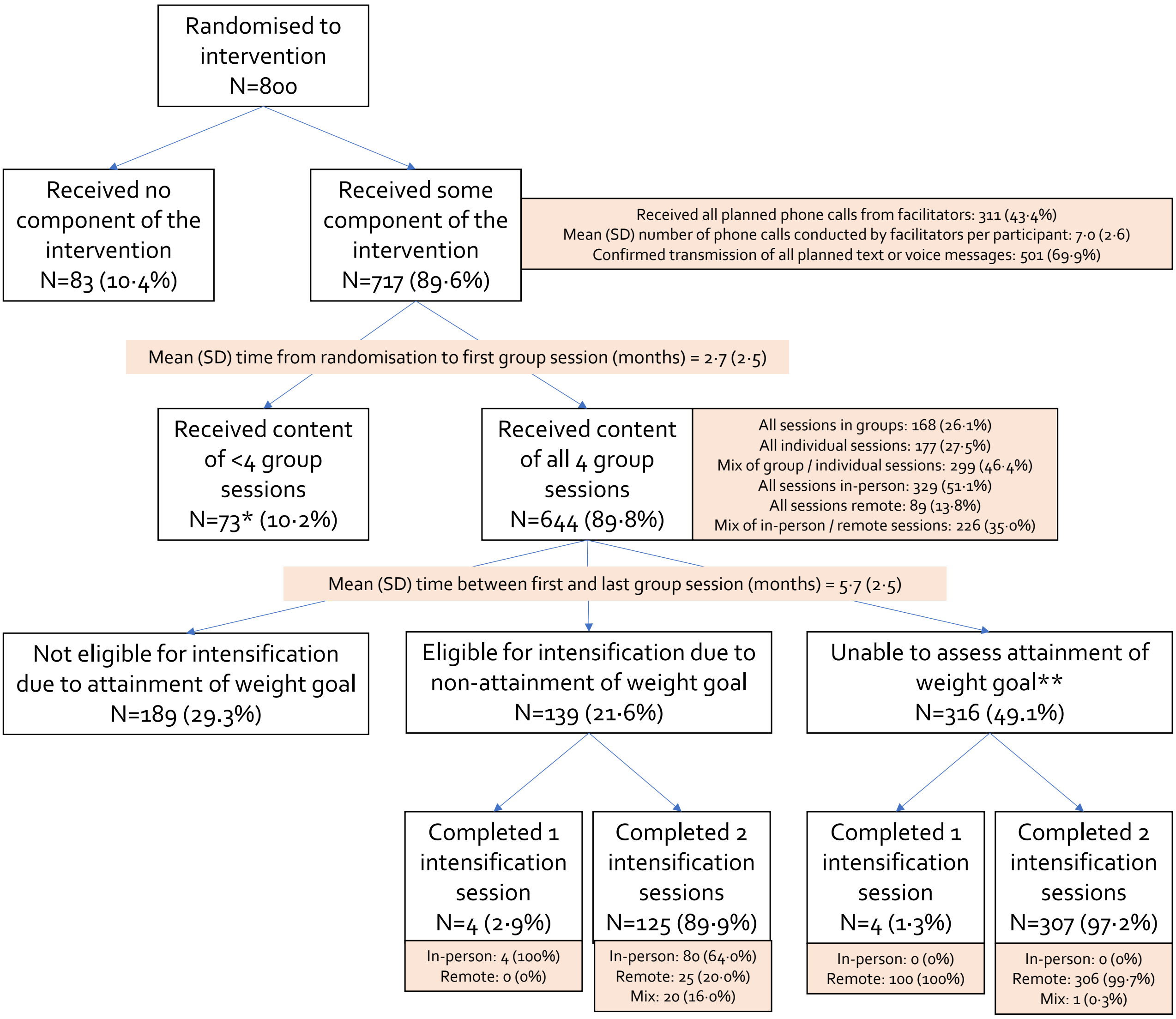

* 1 underwent 1 intensification session and 3 underwent 2 intensification sessions; **weight could not be reliably measured due to COVID-19 related lockdowns

© 2022 Tandon N et al. *JAMA Network Open.*

eFigure 4. Changes In Continuous Secondary Outcomes Over Time

A. Fasting plasma glucose

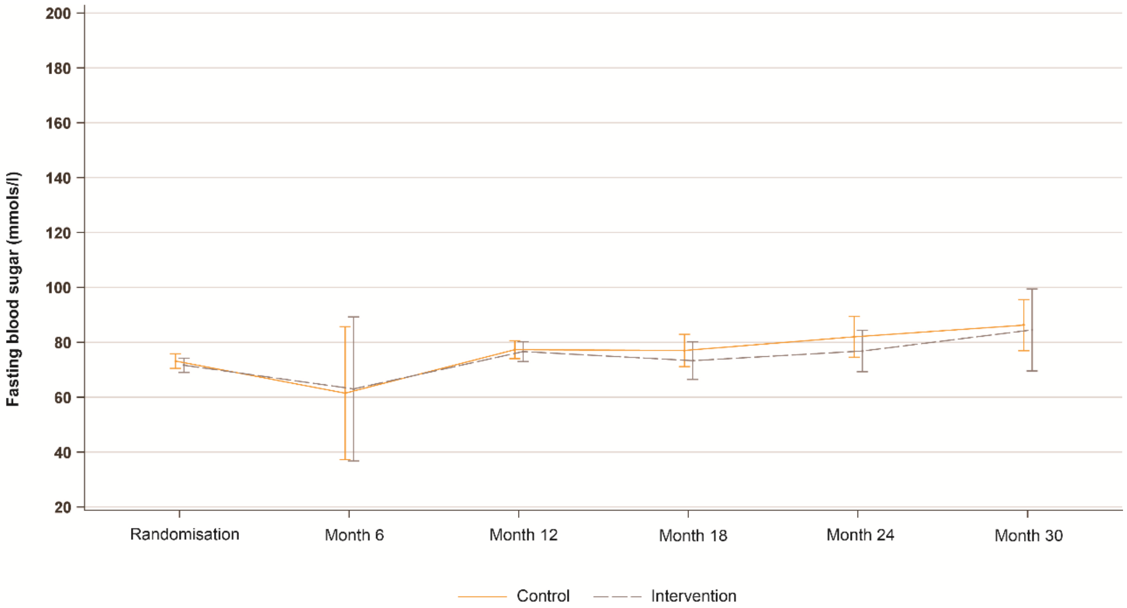

Number of patients						
Control	801	22	539	189	132	46
Intervention	800	23	497	181	134	35

B. Body weight

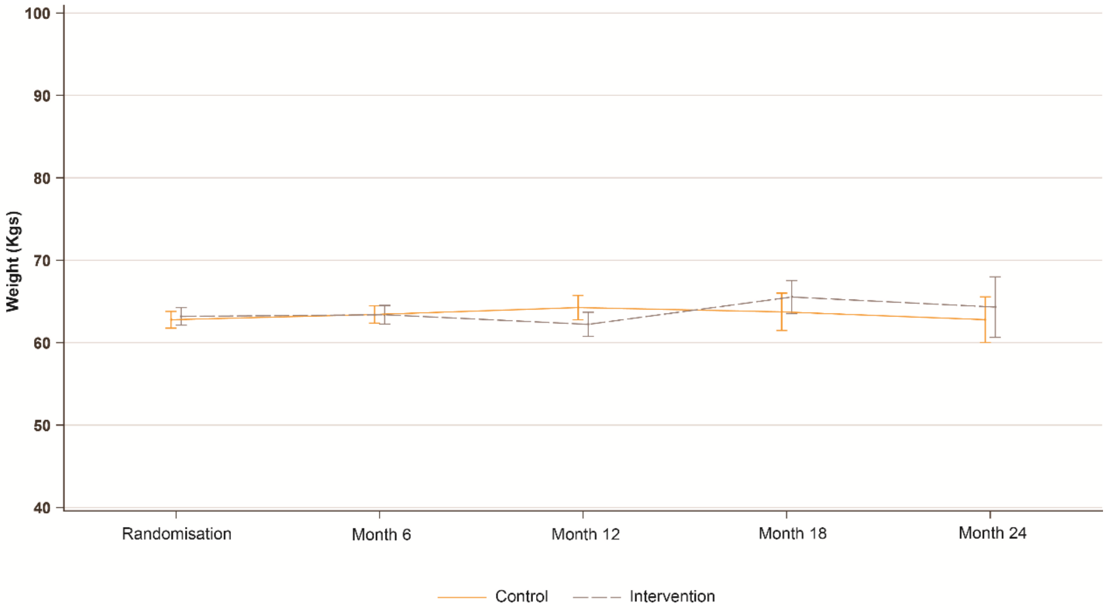

Number of patients					
Control	521	525	260	124	48
Intervention	503	484	250	131	33

© 2022 Tandon N et al. *JAMA Network Open.*

C. Systolic blood pressure

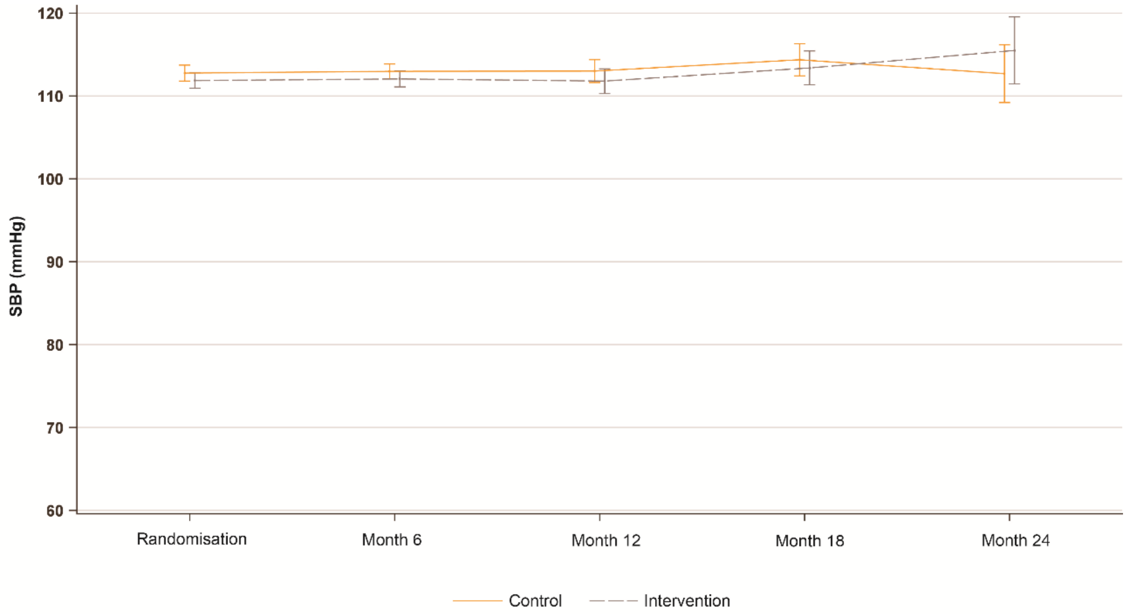

Number of patients					
Control	521	524	259	122	48
Intervention	503	483	248	130	34

D. Diastolic blood pressure

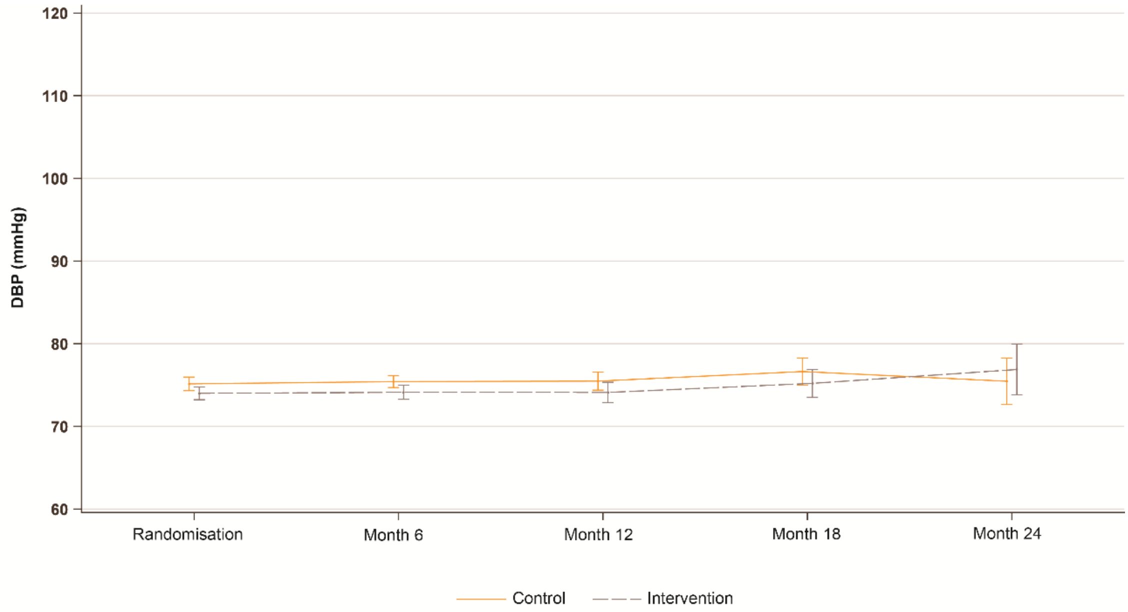

Number of patients					
Control	522	531	260	122	48
Intervention	505	487	249	130	34

© 2022 Tandon N et al. *JAMA Network Open.*

E. Caloric intake

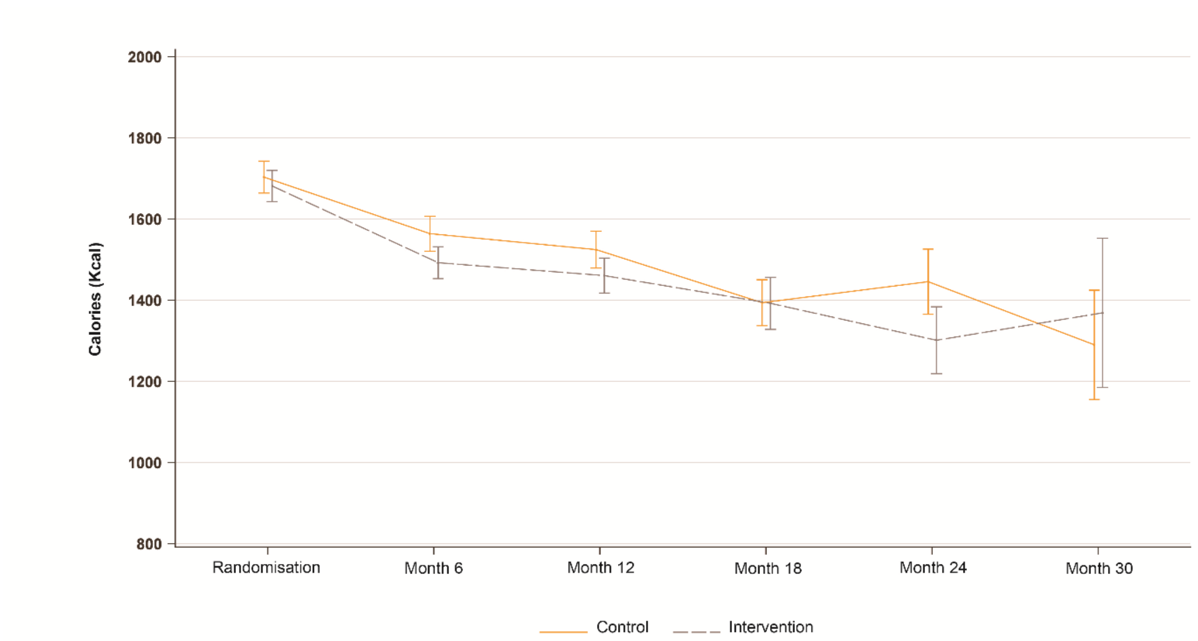

Number of patients	Randomisation	Month 6	Month 12	Month 18	Month 24	Month 30
Control	799	542	555	248	129	40
Intervention	793	527	514	236	120	31

© 2022 Tandon N et al. *JAMA Network Open.*

eFigure 5. Kaplan-Meier Plot for Development of Type 2 Diabetes

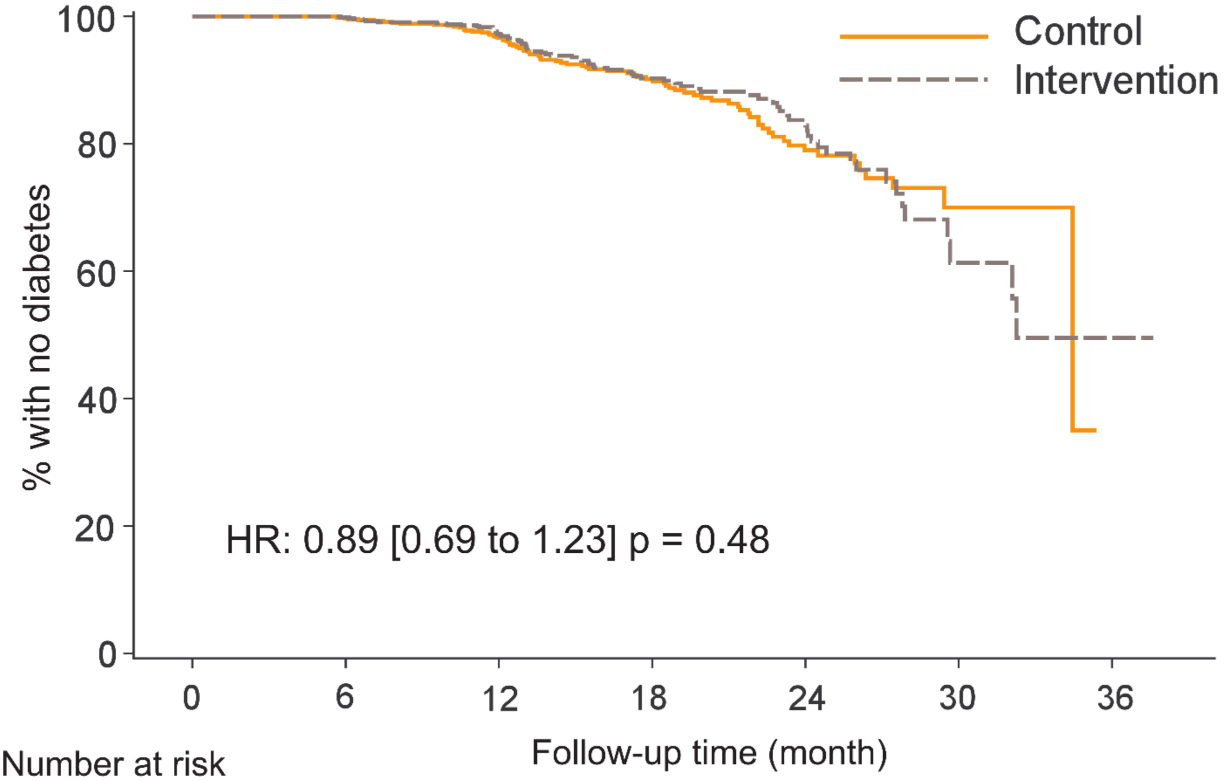

Number at risk

Control	801	681	570	274	103	16	0
Intervention	800	647	556	251	102	18	2

© 2022 Tandon N et al. *JAMA Network Open.*

eFigure 6. Prespecified Subgroup Analyses for Primary Outcome

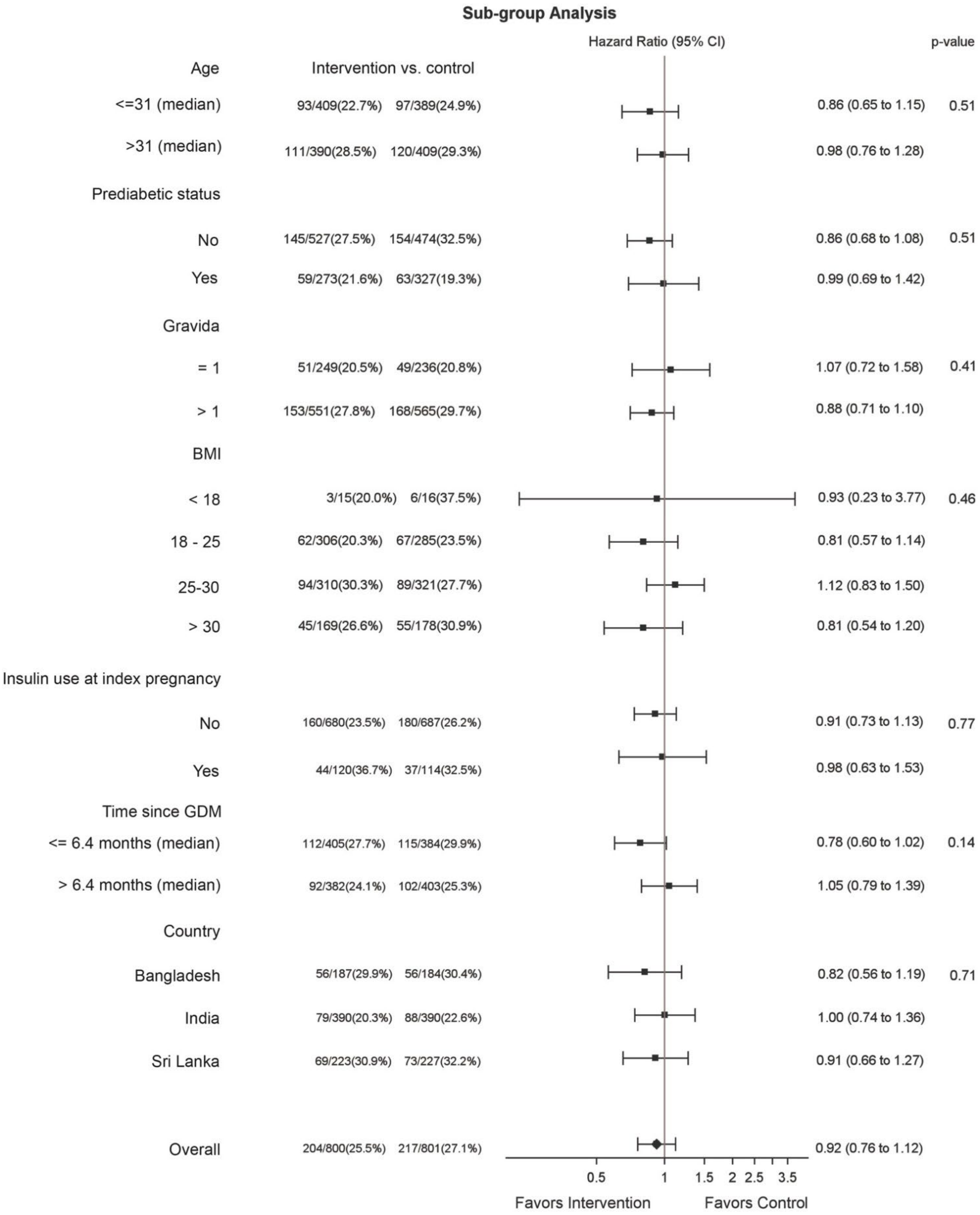

© 2022 Tandon N et al. *JAMA Network Open.*
