# Supplementary material for: Effects of a Lifestyle Intervention to Prevent Deterioration in Glycemic Status Among South Asian Women With Recent Gestational Diabetes: A Randomized Clinical Trial
Source: JAMA Netw Open. 2022 Mar 2;5(3):e220773. doi: 10.1001/jamanetworkopen.2022.0773 (PMC8892226; doi:10.1001/jamanetworkopen.2022.0773)
Supplement: Supplement 3. — Group Authors [file jamanetwopen-e220773-s003.pdf]

*Group Name(s): LIVING Collaborative Group							
***First Name and Middle Initial(s)**	***Last Name**	***Suffix (eg, Jr, III)**	Academic Degrees	Institution	Location (city, state/province, country)	Role or Contribution, eg, chair, principal investigator	Group (if more than 1 Group listed in the byline) and/or Subgroup (eg, Steering Committee)
Md. Muniruzzaman	Siddiqui			Mohammadpur Fertility Services & Training Centre [MFSTC]	Dhaka, Bangladesh		
Ishrat	Jahan			Maternal and Child Health Training Institute, Azimpur [N	Dhaka, Bangladesh		
Mohammad Hussain	Chowdhury			Marie Stopes Clinic	Dhaka, Bangladesh		
Md.Faruque	Pathan			Bangladesh Institute of Research and Rehabilitation for	Dhaka, Bangladesh		
Bishwajit	Bhowmik			Bangladesh Institute of Research and Rehabilitation for	Dhaka, Bangladesh		
Prema	Varthakavi			TNM College & BYL Nair Ch. Hospital Mumbai	Mumbai, India		
Nikhil	Bhagwat			TNM College & BYL Nair Ch. Hospital Mumbai	Mumbai, India		
Vaibhavi	Mungekar			TNM College & BYL Nair Ch. Hospital Mumbai	Mumbai, India		
Rekha	Fernandes			TNM College & BYL Nair Ch. Hospital Mumbai	Mumbai, India		
Vrinda	Pednekar			TNM College & BYL Nair Ch. Hospital Mumbai	Mumbai, India		
Nalini	Shah			GSMC and KEM Hospital, Mumbai	Mumbai, India		
Tushar	Bandgar			GSMC and KEM Hospital, Mumbai	Mumbai, India		
Swati	Jhadav			GSMC and KEM Hospital, Mumbai	Mumbai, India		
Arti	Utekar			GSMC and KEM Hospital, Mumbai	Mumbai, India		
Urjita Ramchandra	Sarnobat			GSMC and KEM Hospital, Mumbai	Mumbai, India		
Neelam	Jaguste			GSMC and KEM Hospital, Mumbai	Mumbai, India		
Arti	More			GSMC and KEM Hospital, Mumbai	Mumbai, India		
Kedar Narvenkar	Narvenkar			Goa Medical College	Goa, India		
Guruprasad	Padnekar			Goa Medical College	Goa, India		
Ajit Arvind	Nagarsenkar			Goa Medical College	Goa, India		
Sachina Vithu	Satarkar			Goa Medical College	Goa, India		
Praciya Shyam	Goankar			Goa Medical College	Goa, India		
Sneha	Chari			Goa Medical College	Goa, India		
Retakshi	Ghadi			Goa Medical College	Goa, India		
Nupur	Phadte			Goa Medical College	Goa, India		
Mabel Anne	Alvares			Goa Medical College	Goa, India		
Mrunali	Gaude			Goa Medical College	Goa, India		
Shiwani	Dadwal			Goa Medical College	Goa, India		
Sailee	Prabhu			Goa Medical College	Goa, India		
Sanjay	Bhadada			PGIMER, Chandigarh	Chandigarh, India		
Neelam	Aggarwal			PGIMER, Chandigarh	Chandigarh, India		
Chandana	Datta			PGIMER, Chandigarh	Chandigarh, India		
Seema	Dahiya			PGIMER, Chandigarh	Chandigarh, India		

*First Name and Middle Initial(s)	*Last Name	*Suffix (eg, Jr, III)	Academic Degrees	Institution	Location (city, state/province, country)	Role or Contribution, eg, chair, principal investigator	Group (if more than 1 Group listed in the byline) and/or Subgroup (eg, Steering Committee)
Deepak	Khandelwal			Maharaja Agrasen Hospital, New Delhi	New Delhi, India		
Soniya	Chahal			Maharaja Agrasen Hospital, New Delhi	New Delhi, India		
Renu	Mann			Maharaja Agrasen Hospital, New Delhi	New Delhi, India		
Rajiv	Singla			Kalpavriksh, New Delhi	New Delhi, India		
Monika	Bhatia			Kalpavriksh, New Delhi	New Delhi, India		
Geetu	Gupta			Kalpavriksh, New Delhi	New Delhi, India		
Bharti	Kharal			Kalpavriksh, New Delhi	New Delhi, India		
Sadishkumar	Kamalanathan			Jawaharlal Institute of Postgraduate Medical Education and Research, Puducherry	Puducherry, India		
Rajan	Palui			Jawaharlal Institute of Postgraduate Medical Education and Research, Puducherry	Puducherry, India		
Jaya Prakash	Sahoo			Jawaharlal Institute of Postgraduate Medical Education and Research, Puducherry	Puducherry, India		
Papa	Dasari			Jawaharlal Institute of Postgraduate Medical Education and Research, Puducherry	Puducherry, India		
Niya	Narayan			Jawaharlal Institute of Postgraduate Medical Education and Research, Puducherry	Puducherry, India		
Varun	Suryadevara			Jawaharlal Institute of Postgraduate Medical Education and Research, Puducherry	Puducherry, India		
Kavi	Priya			Jawaharlal Institute of Postgraduate Medical Education and Research, Puducherry	Puducherry, India		
R	Priya			Jawaharlal Institute of Postgraduate Medical Education and Research, Puducherry	Puducherry, India		
V	Mohan			Madras Diabetes Research Foundation, Chennai	Chennai, India		
Uma	Ram			Madras Diabetes Research Foundation, Chennai	Chennai, India		
Guha	Pradeepa			Madras Diabetes Research Foundation, Chennai	Chennai, India		
Rajasree	Gopinath			Madras Diabetes Research Foundation, Chennai	Chennai, India		
R	Krishnaveni			Madras Diabetes Research Foundation, Chennai	Chennai, India		
U	Ashwini			Madras Diabetes Research Foundation, Chennai	Chennai, India		
E	Chandralekha			Madras Diabetes Research Foundation, Chennai	Chennai, India		
P	Nandhini			Madras Diabetes Research Foundation, Chennai	Chennai, India		
Mala	Dharmalingam			Ramaiah Medical College, Bengaluru	Karnataka, India		
Chitra	Selvan			Ramaiah Medical College, Bengaluru	Karnataka, India		
Pramila	Kalra			Ramaiah Medical College, Bengaluru	Karnataka, India		
Mamta	Sanjeeva			Ramaiah Medical College, Bengaluru	Karnataka, India		
Dev	Sreenivasa			Ramaiah Medical College, Bengaluru	Karnataka, India		
Sowmya	G S			Ramaiah Medical College, Bengaluru	Karnataka, India		
Bhagyamma				Ramaiah Medical College, Bengaluru	Karnataka, India		

*First Name and Middle Initial(s)	*Last Name	*Suffix (eg, Jr, III)	Academic Degrees	Institution	Location (city, state/province, country)	Role or Contribution, eg, chair, principal investigator	Group (if more than 1 Group listed in the byline) and/or Subgroup (eg, Steering Committee)
Nihal	Thomas			Christian Medical College, Vellore	Tamil Naidu, India		
Sahana	Shetty			Christian Medical College, Vellore	Tamil Naidu, India		
Felix	Jebasingh			Christian Medical College, Vellore	Tamil Naidu, India		
Riddhi	Dasgupta			Christian Medical College, Vellore	Tamil Naidu, India		
Jiji	Mathew			Christian Medical College, Vellore	Tamil Naidu, India		
Kavitha	Sankar			Christian Medical College, Vellore	Tamil Naidu, India		
Jansi Vimala	Rani			Christian Medical College, Vellore	Tamil Naidu, India		
Nithya	Devanithi			Christian Medical College, Vellore	Tamil Naidu, India		
Flory	Christina			Christian Medical College, Vellore	Tamil Naidu, India		
Shirley	Newton			Christian Medical College, Vellore	Tamil Naidu, India		
Anisha	Gala			Fernandez Hospital, Hyderabad	Telagana, India		
S	Tarakeswari			Fernandez Hospital, Hyderabad	Telagana, India		
Vidyavati	Patil			Fernandez Hospital, Hyderabad	Telagana, India		
M Bhavana	Reddy			Fernandez Hospital, Hyderabad	Telagana, India		
K	Vijayalakshmi			Fernandez Hospital, Hyderabad	Telagana, India		
G Vasantha	Rani			Fernandez Hospital, Hyderabad	Telagana, India		
Sunil	Fernando			Colombo North Teaching Hospital, Colombo	Srilanka		
Carmaline	Motha			Colombo North Teaching Hospital, Colombo	Srilanka		
Sanjeeda	Baduge			Colombo North Teaching Hospital, Colombo	Srilanka		
Sachini Rangana Withanage	Withanage			Colombo North Teaching Hospital, Colombo	Srilanka		
Dilumi	Jayawickrama			Colombo North Teaching Hospital, Colombo	Srilanka		
Saumya	Hapuarachchi			Colombo North Teaching Hospital, Colombo	Srilanka		
Sripali	Amarasinghe			Colombo North Teaching Hospital, Colombo	Srilanka		
Athula	Kaluarachchi			De Soysa Hospital, Colombo	Srilanka		
MNM	Rishad			De Soysa Hospital, Colombo	Srilanka		
Sachini	Ranasinghe			De Soysa Hospital, Colombo	Srilanka		
Madara	Jayanetti			De Soysa Hospital, Colombo	Srilanka		
Aaisha	Azam			De Soysa Hospital, Colombo	Srilanka		
Ravija	Ramasinghe			De Soysa Hospital, Colombo	Srilanka		
H.V.L	Rangika			De Soysa Hospital, Colombo	Srilanka		
Rukshan	Fernandopulle			Colombo South teaching hospital, Colombo	Srilanka		
Madura	Jayawardena			Colombo South teaching hospital, Colombo	Srilanka		
Sheran	Siyambalapitiya			Colombo South teaching hospital, Colombo	Srilanka		
Gayan	Liyanage			Colombo South teaching hospital, Colombo	Srilanka		
Piyumalie	Hettiarachchi			Colombo South teaching hospital, Colombo	Srilanka		
Dimuthu	Kaluarachchi			Colombo South teaching hospital, Colombo	Srilanka		

***First Name and Middle Initial(s)**	***Last Name**	***Suffix (eg, Jr, III)**	Academic Degrees	Institution	Location (city, state/province, country)	Role or Contribution, eg, chair, principal investigator	Group (if more than 1 Group listed in the byline) and/or Subgroup (eg, Steering Committee)
Uthpala	Chandradeva			Colombo South teaching hospital, Colombo	Srilanka		
Kalawila	Withanage			Colombo South teaching hospital, Colombo	Srilanka		
Rangana	Amarasinghe			Colombo South teaching hospital, Colombo	Srilanka		
Deepa	Nandani			Colombo South teaching hospital, Colombo	Srilanka		
Jeyakumar	Sabaretnam			Negombo District General hospital, Colombo	Srilanka		
Malaka	Amarasena			Negombo District General hospital, Colombo	Srilanka		
M. D. Radhika	Sriyakanthi			Negombo District General hospital, Colombo	Srilanka		
Kanchana	Peiris			Negombo District General hospital, Colombo	Srilanka		
Thiyagarajah	Kadotgajan			Castle Street Hospital, Colombo	Srilanka		
Milinda	Gamlath			Castle Street Hospital, Colombo	Srilanka		
Asha	Weerasinghe			Castle Street Hospital, Colombo	Srilanka		
A.H.	Samanthi			Castle Street Hospital, Colombo	Srilanka		
Anu	Kaushik			All India Institute of Medical Sciences, New Delhi, India	New Delhi, India		
Neha	Sethi			All India Institute of Medical Sciences, New Delhi, India	New Delhi, India		
Ishita	Agarwal			Centre for Chronic Disease Control, New Delhi, India	New Delhi, India		
Vandana	Garg			Centre for Chronic Disease Control, New Delhi, India	New Delhi, India		
Kanika	Chopra			Centre for Chronic Disease Control, New Delhi, India	New Delhi, India		
Divya	Soni			Centre for Chronic Disease Control, New Delhi, India	New Delhi, India		
Purnima Rao	Jevaji			George Institute, Hyderabad, India	Telagana, India		
Pavitra	Madhira			George Institute, Hyderabad, India	Telagana, India		
Thanushanthan	Jeevaraja			Remedium One, Colombo, Sri Lanka	Srilanka		
Shehan	Gnanapragasam			Remedium One, Colombo, Sri Lanka	Srilanka		
Shabnam	Sheuly			International Centre for Diarrhoeal Disease Research, D	Dhaka, Bangladesh		
Nazia	Ferdowsh			International Centre for Diarrhoeal Disease Research, D	Dhaka, Bangladesh		
Tarana	Mustari			International Centre for Diarrhoeal Disease Research, D	Dhaka, Bangladesh		
Shahnaz Parvin	Munni			International Centre for Diarrhoeal Disease Research, D	Dhaka, Bangladesh		
Azmira	Khatun			International Centre for Diarrhoeal Disease Research, D	Dhaka, Bangladesh		
Marzia	Sultana			International Centre for Diarrhoeal Disease Research, D	Dhaka, Bangladesh		
Rifat Hasan	Shammi			International Centre for Diarrhoeal Disease Research, D	Dhaka, Bangladesh		
Sabrina	Ahmed			International Centre for Diarrhoeal Disease Research, D	Dhaka, Bangladesh		
Nantu	Chakma			International Centre for Diarrhoeal Disease Research, D	Dhaka, Bangladesh		
Helen	Monaghan			The George Institute for Global Health, Australia	Australia		
Sindhu	Prasad			The George Institute for Global Health, Australia	Australia		
Amrutha	Nagarajaiah			The George Institute for Global Health, Australia	Australia		
Prakash	Velappan			George Clinical, Bangalore	Karnataka, India		
Koushik	Gade			George Clinical, Bangalore	Karnataka, India		

***First Name and Middle Initial(s)**	***Last Name**	***Suffix (eg, Jr, III)**	Academic Degrees	Institution	Location (city, state/province, country)	Role or Contribution, eg, chair, principal investigator	Group (if more than 1 Group listed in the byline) and/or Subgroup (eg, Steering Committee)
Swathi	Pagadala			George Clinical, Bangalore	Karnataka, India		
Prithvishree B	Radhakrishna			George Clinical, Bangalore	Karnataka, India		
Ambika	Yoganathan			George Clinical, Bangalore	Karnataka, India		
Sumathi	Senthil			George Clinical, Bangalore	Karnataka, India		
Ravikumar	Tummapudi			George Clinical, Bangalore	Karnataka, India		
Ullas	Arabhavi			George Clinical, Bangalore	Karnataka, India		
Catherine	Lombard			Monash University, Australia	Australia		
Dewan	Alam			Centre for Global Health Research, Canada	Canada		
