# Supplementary material for: Effects of a Lifestyle Intervention to Prevent Deterioration in Glycemic Status Among South Asian Women With Recent Gestational Diabetes: A Randomized Clinical Trial
Source: JAMA Netw Open. 2022 Mar 2;5(3):e220773. doi: 10.1001/jamanetworkopen.2022.0773 (PMC8892226; doi:10.1001/jamanetworkopen.2022.0773)
Supplement: Supplement 4. — Data Sharing Statement [file jamanetwopen-e220773-s004.pdf]

Data Sharing Statement

Tandon N, Gupta Y, Kapoor D, et al. Effects of a lifestyle intervention to prevent deterioration in glycemic status among South Asian women with recent gestational diabetes: a randomized clinical trial. JAMA Netw Open. 2022;5(3):e220773. doi:10.1001/jamanetworkopen.2022.0773

Data
Data available: No

Additional Information
Explanation for why data not available: Data, protocols, and all documentation around the analyses presented here will be made available 12 months after publication to academic and other researchers after approval of a Data Access Request. A request form can be obtained by email to the corresponding author or to DSC@georgeinstitute.org.
